# Increase in hepatic and decrease in peripheral insulin clearance characterize abnormal temporal patterns of serum insulin in diabetic subjects

**DOI:** 10.1038/s41540-018-0051-6

**Published:** 2018-03-14

**Authors:** Kaoru Ohashi, Masashi Fujii, Shinsuke Uda, Hiroyuki Kubota, Hisako Komada, Kazuhiko Sakaguchi, Wataru Ogawa, Shinya Kuroda

**Affiliations:** 1 Department of Biological Sciences, Graduate School of Science, University of Tokyo, 7-3-1 Hongo, Bunkyo-ku, Tokyo 113-0033 Japan; 2 Molecular Genetics Research Laboratory, Graduate School of Science, University of Tokyo, 7-3-1 Hongo, Bunkyo-ku, Tokyo 113-0033 Japan; 3 Division of Integrated Omics, Research Center for Transomics Medicine, Medical Institute of Bioregulation, Kyushu University, 3-1-1 Maidashi, Higashi-ku, Fukuoka, Fukuoka 812-8582 Japan; 4 Division of Diabetes and Endocrinology, Department of Internal Medicine, Kobe University Graduate School of Medicine, 7-5-1 Kusunoki-cho, Chuo-ku, Kobe, Hyogo 650-0017 Japan; 5 CREST, Japan Science and Technology Corporation, 7-3-1 Hongo, Bunkyo-ku, Tokyo 113-0033 Japan

## Abstract

Insulin plays a central role in glucose homeostasis, and impairment of insulin action causes glucose intolerance and leads to type 2 diabetes mellitus (T2DM). A decrease in the transient peak and sustained increase of circulating insulin following an infusion of glucose accompany T2DM pathogenesis. However, the mechanism underlying this abnormal temporal pattern of circulating insulin concentration remains unknown. Here we show that changes in opposite direction of hepatic and peripheral insulin clearance characterize this abnormal temporal pattern of circulating insulin concentration observed in T2DM. We developed a mathematical model using a hyperglycemic and hyperinsulinemic-euglycemic clamp in 111 subjects, including healthy normoglycemic and diabetic subjects. The hepatic and peripheral insulin clearance significantly increase and decrease, respectively, from healthy to borderline type and T2DM. The increased hepatic insulin clearance reduces the amplitude of circulating insulin concentration, whereas the decreased peripheral insulin clearance changes the temporal patterns of circulating insulin concentration from transient to sustained. These results provide further insight into the pathogenesis of T2DM, and thus may contribute to develop better treatment of this condition.

## Glucose homeostasis: roles of insulin clearance

Type 2 diabetes mellitus (T2DM) is one of the fastest growing public health problems, characterized by chronic hyperglycemia with the failure of glucose homeostasis. Evaluating alteration in biological functions regulating circulating glucose concentration is complicated due to the mutual relation between circulating glucose and insulin. A team led by Wataru Ogawa at Kobe University designed clinical experiments for breaking such feedback relations, and a team led by Shinya Kuroda at University of Tokyo developed mathematical models for specifically quantifying the functions from the clinical data. The estimated model parameters revealed the significant increase in hepatic and decrease in peripheral insulin clearance, which occur before and after insulin delivery into systemic circulation, respectively, from healthy to T2DM subjects. Model analysis suggested these insulin clearances centrally regulate the dynamics of circulating insulin concentration in the glucose-insulin regulatory system.

## Introduction

Insulin is the major anabolic hormone regulating the glucose homeostasis. The impaired action of insulin is a characteristic of type 2 diabetes mellitus (T2DM),^1^ accompanied by abnormality in the temporal patterns of circulating insulin concentration.^2–4^ The circulating insulin concentration changes over the course of 24 h, including a persistently low level during fasting and a surge in response to food ingestion, consisting of basal and additional secretions from the pancreas, respectively.^5,6^

Ability of additional insulin secretion is assessed by the oral glucose tolerance test (OGTT),^7^ in which a subject’s ability to tolerate the glucose load (glucose tolerance) is evaluated by measuring the circulating glucose concentration after an overnight fast (fasting plasma glucose concentration; FPG) and again 2 h after a 75-g oral glucose load (2-h post-load glucose concentration; 2-h PG).^8^ During this test, the circulating insulin concentration transiently increases and then continuously increases or decreases, known as the early and late phases of insulin secretion, respectively.^9,10^ The direct contribution of circulating glucose concentration to circulating insulin concentration is assessed by the use of an intravenous glucose tolerance test (IVGTT).^11^ This test excludes the effects of intestinal absorption of glucose and incretins secretion that trigger insulin secretion, thus permitting quantitative estimates of the ability of circulating glucose to initiate insulin secretion. During this test, the circulating insulin concentration transiently increases during the first 10 min and then continuously increases during the following 120 min, which are known as the first and second phase of insulin secretion, respectively.^12^

These temporal patterns of circulating insulin concentration differ between normal glucose tolerance (NGT), borderline type, and T2DM. Based on an OGTT, a subject with FPG <110 mg/dL (6.1 mM) and 2-h PG <140 mg/dL (7.8 mM) is categorized as NGT. A subject with FPG of 110–125 mg/dL (6.1–6.9 mM) or 2-h PG of 140–199 mg/dL (7.8–11.0 mM) is categorized as borderline type, and those with FPG ≥126 mg/dL (7.0 mM) or 2-h PG ≥200 mg/dL (11.1 mM) as T2DM.^8^ In general, plasma insulin concentration during the late-phase secretion of an OGTT in borderline type subjects is higher than in NGT subjects, whereas the concentration during the early-phase secretion is similar in NGT and borderline type subjects.^9,10^ Plasma insulin concentration during the first-phase secretion of an IVGTT decreases as glucose intolerance progresses, whereas that during the second-phase secretion is relatively maintained.^2–4^ Such changes of the temporal patterns of circulating insulin concentration during the progression of glucose intolerance from NGT to T2DM suggest that these temporal patterns are involved in the maintenance and impairment of glucose homeostasis. Together with the measurement of circulating glucose concentration, the time course of circulating insulin concentration is used to assess the insulin secretion from the pancreas and insulin sensitivity.

However, it is difficult to assess the insulin secretion and sensitivity of body tissues directly from the circulating insulin concentration because of the negative feedback between circulating insulin and glucose. A rise in circulating glucose concentration stimulates insulin secretion, and the resultant rise in circulating insulin concentration stimulates glucose uptake, causing circulating glucose concentration to fall. This feedback means there is mutual dependence between glucose and insulin, making it difficult to distinguish the effect of insulin secretion and sensitivity directly from the circulating insulin concentration.^13^

To directly assess insulin secretion without the effect of the feedback from insulin to glucose, DeFronzo et al.^13^ developed the hyperglycemic clamp technique, in which insulin secretion is measured while circulating glucose concentration is at a fixed hyperglycemic plateau maintained by exogenous continuous glucose infusion. The measurements of circulating insulin concentration during the first 10 min and after 10 min are used to assess the insulin secretion ability and are known as the first- and second-phase insulin secretions, respectively.^13,14^

Conversely, to directly assess insulin sensitivity without the effect of the feedback from glucose to insulin, the hyperinsulinemic-euglycemic clamp was developed.^13^ In this method, circulating insulin concentration is maintained at a fixed hyperinsulinemic plateau and circulating glucose at a fixed normal plateau by continuous infusion of both insulin and glucose. Tissue insulin sensitivity is defined as the ratio of the glucose infusion rate to the circulating insulin concentration when they reach plateaus.^13,14^

The body controls the circulating insulin concentration by balancing insulin secretion and insulin clearance. The major organs responsible for insulin clearance are the liver, which removes portal insulin during first-pass transit,^15,16^ and insulin-sensitive tissues such as muscle, which remove insulin from the systemic circulation.^17^ The insulin clearance from portal vein in the liver and from peripheral plasma in other organs is called hepatic and peripheral insulin clearance, respectively. Although the relationship between changes of insulin clearance and the progression of glucose intolerance have been reported, the effects of insulin clearance are controversial. Some studies found that during the progression of glucose intolerance, insulin clearance decreased,^18–21^ whereas hepatic insulin clearance increased^22^ or decreased.^18,23^ Thus, the hepatic and peripheral insulin clearances were not explicitly distinguished, making it difficult to interpret the effect of both types.

Hepatic insulin clearance cannot be assessed directly from circulating insulin concentration because insulin is extracted from the liver before secreted insulin is delivered into the systemic circulation. However, insulin is secreted at an equimolar ratio with C-peptide, a peptide cleaved from proinsulin to produce insulin, which is not extracted in the liver. Thus, by measuring circulating C-peptide concentration simultaneously with circulating insulin concentration, the pre-hepatic insulin concentration can be accurately assessed. The C-peptide index, which is the ratio of circulating glucose to C-peptide concentration, is an index of insulin secretion with clinical utility.^24^ Hepatic insulin clearance is clinically quantified as the ratio of circulating insulin to C-peptide concentration during the first 10 min under the hyperglycemic clamp condition.^25^

The clinical indices of insulin secretion and clearance are indirect measures because they are obtained from temporal patterns of circulating concentrations, which are simultaneously affected by insulin secretion and clearance. Therefore, the clinical index of insulin secretion implicitly involves the effect of insulin clearance and vice versa. Mathematical models have been developed for specifically quantifying insulin secretion, sensitivity, and clearance abilities from temporal patterns of circulating concentration by accounting for this mutual dependence.^26–28^ The model known as the minimal model is used to estimate insulin sensitivity and insulin secretion abilities for each individual based on the time courses of circulating glucose and insulin concentrations during IVGTT.^29^ Furthermore, from the parameters of the model, Bergman et al.^29^ identified a relationship between the subject’s glucose intolerance and the product of insulin secretion and sensitivity.

We previously developed a mathematical model based on time courses of plasma glucose and serum insulin during consecutive hyperglycemic and hyperinsulinemic-euglycemic clamp conditions, and estimated the parameters of insulin secretion, sensitivity, and peripheral insulin clearance for each subject. We found that peripheral insulin clearance significantly decreased from NGT to borderline type to T2DM.^30^ However, the hepatic and peripheral insulin clearance could not be distinguished because C-peptide was not incorporated in the model.^30^

Hepatic insulin clearance is calculated as the difference between pre-hepatic and post-hepatic insulin concentrations assessed by comparing circulating C-peptide and insulin concentrations, because C-peptide, unlike insulin, is not removed by the liver. Since the circulating C-peptide concentration is also controlled by its secretion and clearance, a mathematical model for C-peptide kinetics was developed.^31^ The models for circulating insulin and C-peptide have been used to estimate the secretion and kinetics of insulin and C-peptide, as well as hepatic insulin clearance.^32–39^ However, peripheral insulin clearance was not assessed in the models, because exogenous insulin infusion, which is required for accurate estimation of peripheral insulin clearance, was not performed.

Recently, Polidori et al.^40^ reported that both hepatic and extrahepatic insulin clearance, corresponding to peripheral insulin clearance, can be estimated by modeling analysis using plasma insulin and C-peptide concentrations obtained from the insulin-modified frequently sampled IVGTT. The parameters of hepatic and peripheral insulin clearance in the model were not highly correlated, suggesting that the two types of insulin clearance are regulated differently. In addition, hepatic insulin clearance was negatively correlated with insulin secretion, and peripheral insulin clearance was positively correlated with insulin sensitivity. However, hepatic and peripheral insulin clearance in T2DM subjects and the roles of both types of clearance in the changes in temporal pattern of circulating insulin concentration during the progression of glucose intolerance have yet to be examined.

In this study, we developed a mathematical model based on the time course of the serum insulin and C-peptide concentrations during consecutive hyperglycemic and hyperinsulinemic-euglycemic clamp conditions, and estimated the hepatic and peripheral insulin clearance for each subject. The parameters from 111 subjects (47 NGT, 17 borderline type, and 47 T2DM) showed a significant increase in hepatic insulin clearance and significant decrease in peripheral insulin clearance from NGT to borderline type and T2DM, respectively. We also found that hepatic and peripheral insulin clearance play distinct roles in the abnormal temporal patterns of serum insulin concentration from NGT to borderline type and T2DM, namely an increase in hepatic insulin clearance reduces the amplitude of serum insulin concentration, whereas a decrease in peripheral insulin clearance changes the temporal patterns of serum insulin concentration from transient to sustained.

## Results

### Consecutive hyperglycemic and hyperinsulinemic-euglycemic clamp data

**Fig. 1:**
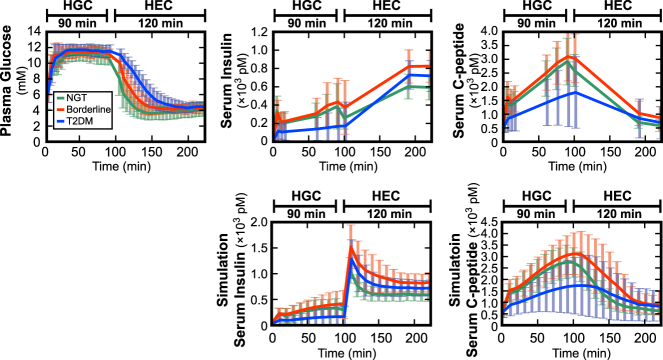
Concentrations of plasma glucose, serum insulin, and C-peptide during consecutive hyperglycemic and hyperinsulinemic-euglycemic clamps. The mean ± SD among the subjects for NGT (green, *n* = 50), borderline type (red, *n* = 18), and T2DM (blue, *n* = 53) of experimental (upper 3 panels) and simulation with *Model VI* (lower 2 panels) time courses are shown. Hyperglycemic clamp (HGC) was performed for 90 min and hyperinsulinemic-euglycemic clamp (HEC) for 120 min with a 10-min interval. The plasma glucose level is the average value calculated every 5 min of the measurements made every 1 min, and the serum insulin and C-peptide levels are measured values at sampling time (Methods). Simulation time courses are plotted every 10 min. Supplementary Figure S1 and Supplementary Table S1 illustrate the significant difference of concentrations at each time point among the three groups

We calculated the averaged time courses of concentrations of plasma glucose, serum insulin, and C-peptide during consecutive hyperglycemic and hyperinsulinemic-euglycemic clamp conditions of NGT (*n* = 50), borderline type (*n* = 18), and T2DM (*n* = 53) (Fig. 1, Supplementary Figure S1).^14,30^ During the hyperglycemic clamp, plasma glucose concentrations at the hyperglycemic plateau were similar among the NGT, borderline type, and T2DM groups.

Both the first (0–15 min) and second phase of insulin secretion (15–90 min) were clearly observed in the NGT and borderline type subjects, whereas the two phases of insulin secretion were significantly reduced in the T2DM subjects. Serum C-peptide concentration showed a similar increase during the first and second phase of insulin secretion in the NGT and borderline type subjects, whereas serum C-peptide concentration was significantly lower in the T2DM subjects during both phases. Although insulin and C-peptide should be secreted in an equimolar manner, the serum C-peptide concentration was higher than the serum insulin concentration because insulin—but not C-peptide—was removed by the liver and C-peptide clearance in the periphery was slower than insulin clearance.

During the hyperinsulinemic-euglycemic clamp at 100–220 min, serum insulin concentration was at a steady-state plateau of hyperinsulinemia, but serum insulin concentration differed significantly from the NGT to borderline type and T2DM subjects. The average serum insulin concentration of the NGT subjects was lowest and that of the borderline type subjects was highest. These differences indicate that the ability to remove infused insulin from serum is different among the three groups and suggest that the difference lies in the peripheral insulin clearance. The plasma glucose concentration returned to the basal level from hyperglycemia at a different decay rate among the three groups. The average decay rate was lowest in the T2DM subjects and highest in the NGT subjects, suggesting that insulin sensitivity, which is the ability to promote the hypoglycemic effect in response to serum insulin, decreases from NGT to borderline type to T2DM. The serum C-peptide concentration returned to the fasting level in all groups, and differed significantly between the NGT and borderline type subjects. Only insulin was infused during the hyperinsulinemic-euglycemic clamp, indicating that serum C-peptide was derived only from endogenous secretion.

### Mathematical model for serum insulin and C-peptide concentrations

Many mathematical models that reproduce circulating insulin and C-peptide concentrations have been developed.^29,32–37^ We developed six mathematical models based on these models, and the best model was selected for reproducing measured serum insulin and C-peptide concentrations during consecutive hyperglycemic and hyperinsulinemic-euglycemic clamp^14,30^ (Supplementary Figure S2). These models contain serum insulin and C-peptide concentrations including both insulin and C-peptide secretion and their hepatic and peripheral clearance. Plasma glucose perturbation and insulin infusion were used as inputs (Supplementary Figure S1). For each of the 121 subjects, parameters of the six models were estimated by using measured concentrations of plasma glucose, serum insulin, and C-peptide. The resulting model was selected based on minimizing the Akaike information criterion (AIC),^41^ taking into account model complexity and goodness of fit of serum insulin and C-peptide time courses.

**Fig. 2:**
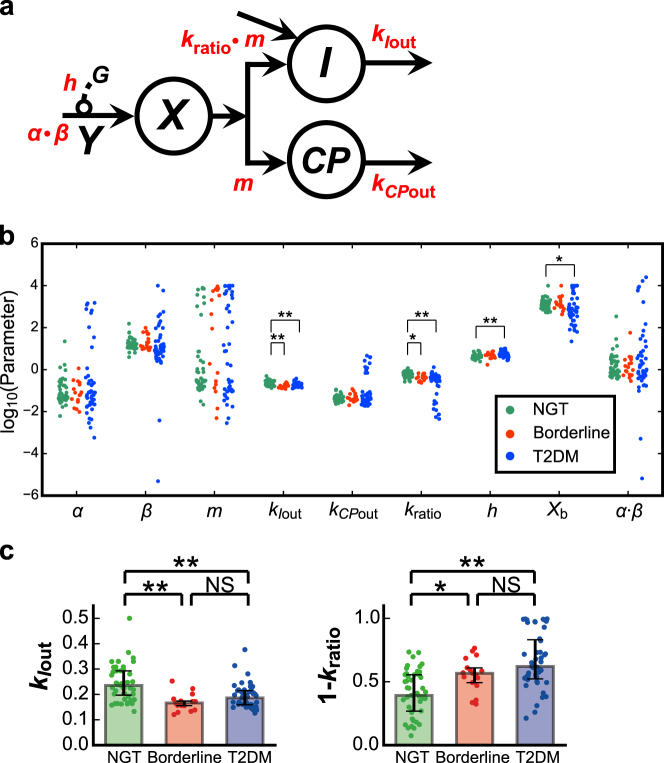
Mathematical model of serum insulin and C-peptide. **a** The structure of the model (see also Eqs. 1–4 and *Model VI* in Supplementary Figure S2). *I* and *CP* are serum insulin and C-peptide concentration, respectively. *X* is the amount of stored insulin and C-peptide, and *Y* is the provision rate controlled by plasma glucose concentration, *G*. Arrows indicate fluxes with corresponding parameters (red). **b** The estimated parameters for the NGT (green), borderline type (red), and T2DM (blue) subjects. Each dot corresponds to the indicated parameter for an individual subject. **c** The parameters of *k*_*I*out_ and (1 − *k*_ratio_), corresponding to peripheral and hepatic insulin clearance, respectively. *P < 0.05, **P < 0.01, NS not significant (two-sided Wilcoxon rank sum test with FDR-correction). Post-hoc statistical power analysis is shown in Supplementary Table S5. The bar and error bar show the median and lower and upper quartiles, respectively. Each dot corresponds to the indicated parameter for an individual subject

The model consisting of four variables (*Model VI* in Supplementary Figure S2) was selected as the best model with the minimum AIC for 76 of 121 subjects (Fig. 2a, Table 1). In this model, the variables *I* and *CP* correspond to serum concentrations of insulin and C-peptide, respectively. The variable *X* corresponds to stored insulin and C-peptide in β-cells or β-cell masses. Because the amounts of stored insulin and C-peptide are equal, a single variable, *X*, is used for both. The variable *Y* is the insulin provision rate depending on plasma glucose concentration. The differential equations of the model are as follows:

1 $$\frac{{{\rm d}Y}}{{{\rm d}t}} = \left\{ {\begin{array}{*{20}{c}} {\alpha \{ \beta (G - h) - Y\} } & {(G > h)} \\ { - \alpha Y} & {(G \le h)} \end{array}} \right.,\quad Y(0) = 0$$

2 $$\frac{{{\rm d}X}}{{{\rm d}t}} = Y - v_{CP{\mathrm{in}}} = \left\{ {\begin{array}{*{20}{c}} {Y - m \cdot X} & {\left( {G > h} \right)} \\ Y & {\left( {G \le h} \right)} \end{array}} \right.,\quad X(0) = X_{\mathrm{b}}$$

3 $$\begin{array}{*{20}{l}}\frac{{{\rm d}I}}{{{\rm d}t}} & = k_{{\mathrm{ratio}}} \cdot v_{CP{\mathrm{in}}} - v_{I{\mathrm{out}}} + influx\\ & = \left\{ {\begin{array}{*{20}{l}} {k_{{\mathrm{ratio}}} \cdot m \cdot X - k_{I{\mathrm{out}}} \cdot \left( {I - I_{\mathrm{b}}} \right) + f(t)} & {\left( {G > h} \right)} \\ { - k_{I{\mathrm{out}}} \cdot \left( {I - I_{\mathrm{b}}} \right) + f(t)} & {\left( {G \le h} \right)} \end{array}} \right.,\quad I(0) = I_{\mathrm{b}}\end{array}$$

4 $$\begin{array}{*{20}{l}}\frac{{{\rm d}CP}}{{{\rm d}t}} & = v_{CP{\mathrm{in}}} - v_{CP{\mathrm{out}}}\\ & = \left\{ {\begin{array}{*{20}{l}} {m \cdot X - k_{CP{\mathrm{out}}} \cdot \left( {CP - CP_{\mathrm{b}}} \right)} & {\left( {G > h} \right)} \\ { - k_{CP{\mathrm{out}}} \cdot \left( {CP - CP_{\mathrm{b}}} \right)} & {\left( {G \le h} \right)} \end{array}} \right.,\quad CP(0) = CP_{\mathrm{b}},\end{array}$$

where *I*_b_ and *CP*_b_ correspond to fasting (basal) serum insulin and C-peptide concentration, respectively, directly given by the measurement, and *X*_b_ is an initial value of *X* to be estimated.

**Table 1:** Comparison of the models based on the Akaike information criterion (AIC)

Model	No. subjects of min AIC	NGT	Borderline	T2DM	AIC mean ± SD
*I*	25	7	4	14	−21.1 ± 22.9*
*II*	5	3	1	1	−19.0 ± 24.2*
*III*	0	0	0	0	−16.2 ± 24.9*
*IV*	7	0	0	7	−17.2 ± 25.3*
*V*	8	4	3	1	−28.4 ± 26.2
*VI*	76	36	10	30	−31.4 ± 25.2
Total	121	50	18	53	

The number of subjects optimal for each model with minimum AIC is shown (see Methods)

*AIC different from *Model VI* (*P* < 0.01, corrected by the number of *t*-tests, multiplied by 5)

Equation 1 describes how insulin provision rate *Y* increases according to *αβ*(*G* − *h*) when *G* > *h*, and decreases with *αY*. This means that provision of *X*, stored amounts of insulin and C-peptide, depends on parameters *α* and *β*, and stimulated only when the plasma glucose concentration exceeds the threshold value, *h*, which corresponds to FPG.

Equation 2 describes how *X* increases according to the provision rate *Y* and decreases according to the insulin and C-peptide secretion *v*_*CP*in_. *v*_*CP*in_ is *X* secreted at the rate *m* when *G* > *h*. Since *X*_b_, which is the initial value of *X*, relates to the insulin and C-peptide secretion when *G* > *h* for the first time during hyperglycemic clamp, *X*_b_ is responsible for the first-phase secretion.^37^

Equation 3 describes how serum insulin concentration *I* increases according to the post-hepatic insulin delivery, *k*_ratio_ · *v*_*CP*in_, and decreases according to peripheral insulin clearance *v*_*I*out_. *I* also increases according to infused insulin, *influx*. *k*_ratio_ · *v*_*CP*in_ is expanded as *k*_ratio_ · *m* · *X*, which corresponds to insulin delivered into peripheral circulation after passage through the liver when *G* > *h*. The parameter *k*_ratio_ is the molar ratio of post-hepatic insulin to C-peptide, which represents the fraction of insulin delivered to the peripheral circulation without being extracted by the liver. Given that C-peptide is not extracted by the liver, *k*_ratio_ can represent the remaining fraction of insulin after the extraction by the liver over the total amount of secreted insulin, and ranges from 0 to 1. Therefore, (1 − *k*_ratio_) represents the fraction of insulin extracted by the liver and not delivered to the peripheral circulation and corresponds to hepatic insulin clearance; *influx* is the insulin infusion rate during hyperinsulinemic-euglycemic clamp. The infusion rate at time *t* is represented by the function *f*(*t*) (Methods). *v*_*I*out_ represents serum insulin degradation with the rate parameter *k*_*I*out_. Therefore, *k*_*I*out_ represents insulin degradation in the periphery and corresponds to peripheral insulin clearance.

Equation 4 describes how serum C-peptide concentration *CP* increases according to the C-peptide secretion *v*_*CP*in_ and decreases according to peripheral C-peptide clearance *v*_*CP*out_. *v*_*CP*in_ is C-peptide secreted and delivered to peripheral serum without hepatic clearance. *v*_*CP*out_ represents serum C-peptide degradation with the rate parameter *k*_*CP*out_.

For the 45 of 121 subjects who were not optimal for this model (Fig. 2a; *Model VI* in Supplementary Figure S2), the distributions of the residual sum of squares (RSS) between modeled and measured concentrations of insulin and C-peptide in this model were not significantly different from the distributions of the RSS of the remaining 76 subjects who were optimal with minimum AIC for this model (Supplementary Figure S3). There also seems to be no bias in the distribution of the NGT, borderline type, and T2DM subjects among the best models (Table 1). *Model VI* was also selected when AIC of each model was calculated using measured time courses of all 121 subjects (Supplementary Table S2). However, in the RSS distributions of all 121 subjects in this model, RSS values of three subjects were relatively high and detected as outliers, and the three subjects were excluded from the analysis in this study (Supplementary Figure S3). In addition, there seems to be no bias of temporal patterns of serum insulin and C-peptide concentrations among the subjects in each model (Supplementary Figure S4, Supplementary Table S3). Therefore, we selected the model (Fig. 2a; *Model VI* in Supplementary Figure S2) for further study because it was able to reproduce time courses of serum insulin and C-peptide concentrations for the remaining 118 subjects. The simulation with *Model VI* (Fig. 1, Supplementary Figure S1) reproduced measured concentrations of insulin and C-peptide, and reflected significant differences among the NGT, borderline type, and T2DM subjects. Seven subjects (one NGT, one borderline type, and five T2DM subjects) were excluded because their model parameters were detected as outlier based on the adjusted outlyingness (Methods), and we analyzed the model for the remaining 111 subjects (47 NGT, 17 borderline type, and 47 T2DM) (Supplementary Table S4).

### Changes in opposite direction of hepatic and peripheral insulin clearance from NGT to borderline type and T2DM

We statistically compared the model parameters among the NGT, borderline type, and T2DM groups (Fig. 2b and Methods). Four of the nine parameters, *k*_*I*out_, *k*_ratio_, *h*, and *X*_b_, were significantly different.

The parameter *k*_*I*out_ is the degradation rate of serum insulin and corresponds to peripheral insulin clearance. The value of *k*_*I*out_ in the NGT subjects was higher than that in the borderline type and T2DM subjects (Fig. 2c), indicating that peripheral clearance decreases in development of glucose intolerance, which is consistent with previous studies.^30,40^

The parameter *k*_ratio_ is the ratio of post-hepatic insulin to C-peptide, and (1 − *k*_ratio_) corresponds to the insulin extracted by the liver, that is, hepatic insulin clearance. The value of (1 − *k*_ratio_) in the NGT subjects was lower than that in the borderline type and T2DM subjects (Fig. 2c), indicating the increase of hepatic insulin clearance in the borderline type and T2DM subjects. This is consistent with an earlier clinical observation.^22^

The parameter *h* is the threshold of plasma glucose concentration for the insulin secretion and corresponds to FPG. This parameter in the T2DM subjects was significantly higher than that in the NGT subjects (Fig. 2b), consistent with the fact that FPG is higher in T2DM.^9,10^

The parameter *X*_b_ is the initial value of *X*, which corresponds to the stored amounts of insulin and C-peptide or β-cell masses before the start of the hyperglycemic clamp. This parameter in the T2DM subjects was significantly lower than that in the NGT subjects (Fig. 2b), consistent with observations that β-cell masses and stored insulin decrease in T2DM patients.^42–44^

Using the same clamp data, we previously showed that insulin secretion decreases from NGT to borderline type to T2DM.^14,30^ In this study, however, the parameters *α* and *β*, related to insulin secretion, did not show any significant differences among the NGT, borderline type, and T2DM subjects, possibly because previously defined insulin secretion^30^ is described by insulin secretion and delivery in this model, which depends on other parameters such as *h*, *m*, *X*_b_, and *k*_ratio_, and the parameters involved in insulin secretion and delivery are too diverse.

The parameters *k*_ratio_, *k*_*I*out_, *k*_*CP*out_, *h*, and *X*_b_ show smaller variations than others (Fig. 2b). This is probably because these parameters are directly related to the measured concentrations of serum insulin and C-peptide and plasma glucose, and therefore can be accurately estimated, whereas other parameters are not, resulting in large variation possibly due to inaccurate estimation.

### Relationship between hepatic and peripheral insulin clearance parameters and clinical indices of serum insulin regulation

**Fig. 3:**
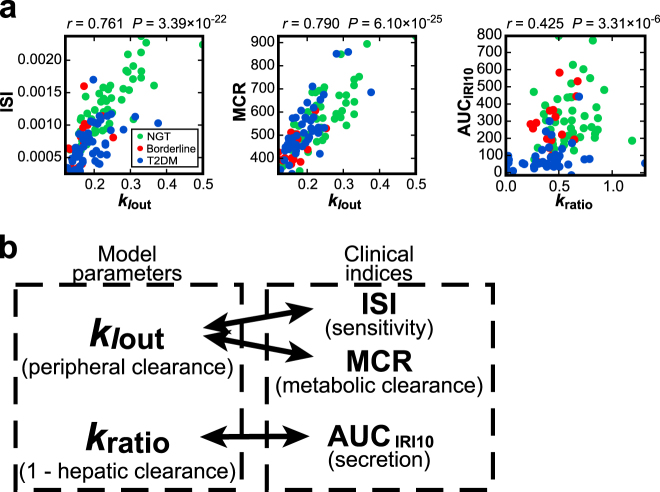
Model parameters showing the highest correlation with clinical indices. **a** Scatter plots for the indicated measured clinical indices versus the highest correlated model parameters (Supplementary Table S6). ISI insulin sensitivity index, MCR metabolic clearance rate, AUC_IRI10_ amount of insulin secretion during the first 10 min of hyperglycemic clamp. Each dot indicates the value of an individual subject. The correlation coefficient, *r*, and the *P* value for testing the hypothesis of no correlation are shown. The partial correlation coefficients among *k*_*I*out_, ISI, and MCR are shown in Supplementary Figure S5. **b** Summary of the model parameters *k*_*I*out_ and *k*_ratio_ showing the highest correlation with the indicated clinical indices

We examined the correlation of the estimated model parameters with clinical indices of circulating insulin regulation among 111 subjects (Fig. 3, Supplementary Table S6). The model parameter showing the highest correlation with insulin sensitivity index (ISI) and with the metabolic clearance rate (MCR), which is the index of insulin clearance (see Methods for details), was peripheral insulin clearance, *k*_*I*out_ (*r* = 0.761 and 0.790, respectively, both *P* < 0.001). This correlation is consistent with our previous finding that peripheral insulin clearance is highly correlated with ISI and MCR.^30^ *k*_*I*out_ is the degradation rate of serum insulin, which depends on the number of insulin receptors on target tissues,^45^ indicating that serum insulin degradation and insulin sensitivity are mutually correlated. Therefore, it is reasonable that *k*_*I*out_ is correlated not only with MCR but also with ISI.

The model parameter showing the highest correlation with insulin secretion during the first phase, AUC_IRI10_ (see Methods), which is the index of insulin secretion, was *k*_ratio_ (*r* = 0.425, *P* < 0.01). Note that (1 − *k*_ratio_) corresponds to hepatic insulin clearance. Because the parameter *k*_ratio_ is the fraction of insulin remaining after the hepatic extraction, its correlation with insulin secretion is reasonable.

In addition, the model parameter showing the highest correlation with both FPG and 2-h PG, the main indices of glucose tolerance, was *h* (*r* = 0.448 and 0.504, respectively, both *P* < 0.001), which is the threshold glucose concentration for insulin secretion. This finding is consistent with *h* corresponding to FPG. The model parameter showing the highest correlation with the clamp disposition index, clamp DI, which is calculated as the product of insulin secretion AUC_IRI10_ and ISI and is the index of glucose tolerance,^14^ was *k*_ratio_ · *k*_*I*out_ (*r* = 0.540, *P* < 0.001). Considering that *k*_ratio_ is related to post-hepatic insulin delivery, and *k*_*I*out_ is related to insulin sensitivity, which depends on the number of insulin receptors on target organs, it is reasonable that the product *k*_ratio_ · *k*_*I*out_ shows the highest correlation with clamp DI, which is also the product of clinically estimated insulin secretion and sensitivity.

### Selective regulation of amplitude and temporal patterns of serum insulin concentration by hepatic and peripheral insulin clearance

**Fig. 4:**
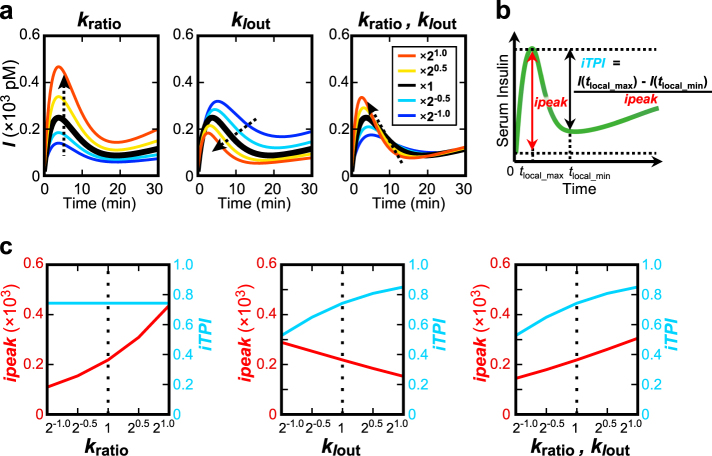
The roles of *k*_ratio_ and *k*_*I*out_ in the amplitude and temporal patterns of serum insulin concentration. **a** Simulated time course of serum insulin concentration *I* during hyperglycemic clamp of subject #3 by changing *k*_ratio_ or *k*_*I*out_ or both by scaling the fitted parameter value with 2^−1.0^, 2^−0.5^, 1, 2^0.5^, and 2^1.0^ (see Methods). Dotted arrows indicate the direction of the change in the temporal pattern as the parameter increases. **b** The definition of *ipeak* (incremental peak) and *iTPI* (incremental transient peak index), reflecting the peak amplitude and the temporal pattern of serum insulin concentration *I*. **c** *ipeak* and *iTPI* of *I* of subject #3 by changing *k*_ratio_ or *k*_*I*out_ or both

Because *k*_*I*out_ and *k*_ratio_ were the parameters showing the highest correlation with clinical indices of insulin sensitivity and secretion, respectively, both of which are related to the progression of glucose intolerance and T2DM, we analyzed the roles of *k*_ratio_ and *k*_*I*out_ in the temporal changes of serum insulin concentration (Fig. 4). We changed the originally estimated values of *k*_ratio_ or *k*_*I*out_ or both by 2^−1^ to 2^1^ times and simulated the time course of *I*, serum insulin concentration, during hyperglycemic clamp for each subject (Supplementary Figure S6). Similar temporal changes of *I* versus changes in the parameters were observed in all 111 subjects, so only the simulation result of subject #3 (NGT) is shown (Fig. 4a).

The time course of *I* with the original parameters in the model of subject #3 showed the transient increase (Fig. 4a, black line). As *k*_ratio_ increased, *I* increased without changing the transient pattern (Fig. 4a, left panel, red line). Indeed, an increase of *k*_ratio_ affects the value of *I* similarly at any time point, because *k*_ratio_ controls the gain of time derivative of *I*. As *k*_*I*out_ increased, *I* decreased and the temporal pattern became more transient with an earlier peak time (Fig. 4a, middle panel, red line). Conversely, as *k*_*I*out_ decreased, *I* increased and the temporal pattern became more sustained with a delayed peak time (Fig. 4a, middle panel, blue line). This result suggests that *k*_*I*out_ controls the shift in the temporal patterns of *I* from transient to sustained. These changes in the temporal pattern of *I* are characterized by a relative decrease in the first-phase secretion and relative increase in the second-phase secretion. Note that the decrease in *k*_*I*out_ also increases the amplitude of *I*.

Because both *k*_ratio_ and *k*_*I*out_ decreased from NGT to borderline type and T2DM (Fig. 2b), we examined the effect of the simultaneous changes of *k*_ratio_ and *k*_*I*out_ on the amplitude and transient/sustained patterns of *I*. When both *k*_ratio_ and *k*_*I*out_ increased with the same ratio, *I* increased during first-phase secretion (0–10 min), whereas *I* decreased during second-phase secretion (10–30 min) (Fig. 4a, right panel, red line). Thus, simultaneous increase of *k*_ratio_ and *k*_*I*out_ results in the increase of peak amplitude of *I* and in changes in the temporal pattern of *I* from sustained to transient.

We quantified the role of *k*_ratio_ and *k*_*I*out_ in the peak amplitude and temporal patterns of *I*. We defined the index *ipeak* (incremental peak) for the peak amplitude of *I*, and the index *iTPI* (incremental transient peak index; modified from Kubota et al.^46^) for the temporal pattern of *I* (Fig. 4b), as follows:

5 $$ipeak = I\left( {t_{{\mathrm{local}}\_{\mathrm{max}}}} \right) - I\left( 0 \right),\quad I\left( {t_{{\mathrm{local}}\_{\mathrm{max}}}} \right) > I\left( {t_{{\mathrm{local}}\_{\mathrm{max}}\_{\mathrm{next}}}} \right),$$

6 $$\begin{array}{*{20}{l}}iTPI = \frac{{I(t_{{\mathrm{local}}\_{\mathrm{max}}}) - I(t_{{\mathrm{local}}\_{\mathrm{min}}})}}{{ipeak}},\\ I(t_{{\mathrm{local}}\_{\mathrm{min}}}) < I(t_{{\mathrm{local}}\_{\mathrm{min}}\_{\mathrm{next}}}),\,t_{{\mathrm{local}}\_{\mathrm{min}}} > t_{{\mathrm{local}}\_{\mathrm{max}}},\end{array}$$

where *I*(*t*) represents *I* at time *t*, *t*_local_max_ is the time at which *I* stops increasing for the first time from 0 min, *t*_local_max_next_ is the next sampling time of *t*_local_max_, *t*_local_min_ is the time at which *I* stops decreasing for the first time after *t*_local_max_, and *t*_local_min_next_ is the next sampling time of *t*_local_min_.

The index *ipeak* is the difference in *I* between the local maximum *I*(*t*_local_max_) and the initial fasting concentration *I*(0) and represents the peak amplitude of *I* during the first-phase secretion. The index *iTPI* is the ratio of the difference of *I* between the local maximum *I*(*t*_local_max_) and the local minimum *I*(*t*_local_min_) of *I* against *ipeak*, which reflects the ratio of *I* during the first- and second-phase secretions. As *iTPI* approaches 1, the difference in *I* between the first- and second-phase secretions becomes larger, meaning that the temporal change of *I* becomes more transient. Conversely, as *iTPI* approaches 0, the difference in *I* between the first- and second-phase secretions becomes smaller, meaning that the temporal change of *I* becomes more sustained.

We calculated *ipeak* and *iTPI* from the simulated time courses of *I* by changing the original estimates of *k*_ratio_ or *k*_*I*out_ or both by 2^−1^ to 2^1^ times. As *k*_ratio_ increased, *ipeak* increased but *iTPI* did not change (Fig. 4c, left panel), indicating that increasing *k*_ratio_ increases the peak amplitude of *I* during the first-phase secretion without changing its temporal pattern. As *k*_*I*out_ increased, *iTPI* increased and *ipeak* decreased (Fig. 4c, middle panel), indicating that increasing *k*_*I*out_ changes the temporal patterns of *I* from sustained to transient and decreases the peak amplitude of *I* during the first-phase secretion.

**Fig. 5:**
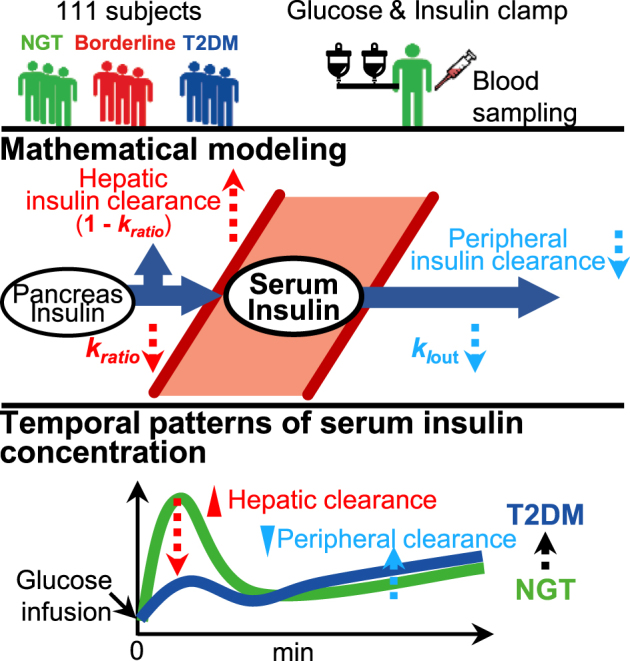
Overview of our study and main results. Mathematical modeling based on hyperglycemic and hyperinsulinemic-euglycemic clamp (glucose and insulin clamp) data in subjects showed changes in opposite direction of hepatic and peripheral insulin clearance from NGT to T2DM. Hepatic insulin clearance (1−*k*_ratio_) increases and peripheral insulin clearance *k*_*I*out_ decreases, characterizing the decrease in peak amplitude and the change in the temporal pattern of serum insulin concentration from transient to sustained, respectively

When both *k*_ratio_ and *k*_*I*out_ increased at the same ratio, both *ipeak* and *iTPI* increased (Fig. 4c, right panel), indicating that increasing both *k*_ratio_ and *k*_*I*out_ increases the peak amplitude of *I* and changes the temporal pattern from sustained to transient. The increase in *ipeak* means that the effect of *k*_ratio_, which increases *ipeak*, is stronger than that of *k*_*I*out_, which decreases *ipeak*. Given that both *k*_ratio_ and *k*_*I*out_ decrease from NGT to borderline type and T2DM, both *ipeak* and *iTPI* decrease (Fig. 5). This finding is consistent with earlier clinical observations that the peak amplitude of circulating insulin concentration during the first-phase secretion decreases and the temporal pattern becomes more sustained during the progression of glucose intolerance.^2–4^

We performed parameter sensitivity analysis on *ipeak* and *iTPI* in the simulation for each model parameter (Table 2, Methods). We compared the median of the parameter for all 111 subjects as the parameter sensitivity index (Table 2). For *ipeak*, the *k*_ratio_ had the significantly highest median, and for *iTPI*,* k*_*I*out_ showed the significantly highest median, indicating that hepatic insulin clearance and peripheral insulin clearance are the most critical parameters controlling the peak amplitude and temporal patterns of serum insulin concentration, respectively.

**Table 2:** Parameter sensitivity analysis for *ipeak* and *iTPI*

Rank	*ipeak*			*iTPI*		
		Median	*P*		Median	*P*
1	*k*_ratio_	1.00		*k*_*I*out_	4.64 × 10^−1^	
2	*m*	3.52 × 10^−1^	6.26 × 10^−38^	*β*	−2.53 × 10^−1^	1.30 × 10^−6^
3	*k*_*I*out_	−2.74 × 10^−1^	1.23 × 10^−36^	*α*	−1.42 × 10^−1^	5.36 × 10^−15^
4	*h*	−3.64 × 10^−4^	4.04 × 10^−34^	*h*	1.38 × 10^−1^	8.70 × 10^−7^
5	*β*	2.59 × 10^−4^	1.23 × 10^−36^	*m*	7.18 × 10^−2^	2.56 × 10^−9^
6	*α*	1.61 × 10^−4^	1.23 × 10^−36^	*k*_ratio_	8.28 × 10^−15^	2.62 × 10^−35^

Medians of the indicated parameters for all 111 subjects were used for parameter sensitivity analysis for *ipeak* and *iTPI* (see Methods). The higher the absolute value of the parameter, the higher the sensitivity. *P* values relative to the median for the top-ranked parameter were determined by two-sided Wilcoxon rank sum test, and the value of <4.17 × 10^–3^ (=0.05/12, corrected by the number of tests, divided by 12) is considered statistically significant

The measured temporal changes of the serum insulin concentration during hyperglycemic clamp (0–90 min) showed no clear difference between the NGT and borderline type subjects (Fig. 1). However, the estimated *k*_ratio_ and *k*_*I*out_ in the NGT subjects were significantly higher than those in the borderline type subjects, suggesting that hepatic and peripheral insulin clearance are not the only parameters responsible for the peak amplitude and temporal patterns of serum insulin concentration, respectively. Other parameters such as insulin secretion, which also affects the temporal changes of serum insulin concentration, may compensate for the temporal changes by insulin clearance between the NGT and borderline type subjects. This also means that changes in the hepatic and peripheral insulin clearance from NGT to borderline type and T2DM cannot be directly assessed from the measured time course of serum insulin concentration, but must be evaluated with a mathematical model.

From Eq. 3, the parameter *k*_ratio_ directly reflects post-hepatic insulin delivery and is involved in the increase in gain of *I*. Therefore, the change of *k*_ratio_ is directly reflected in the peak amplitude, so the sensitivity to *ipeak* becomes 1. This means that hepatic insulin clearance is more responsible for the peak amplitude of serum insulin concentration than the pre-hepatic insulin secretion before extraction by the liver.

The parameter *k*_*I*out_ corresponds to peripheral insulin clearance and is the degradation rate of *I*, meaning that *k*_*I*out_ is the time constant of serum insulin degradation. *k*_*I*out_ is the parameter directly responsible for temporal conversion of input *X*, delivered insulin after the extraction by the liver, into output, *I*. Thus, *k*_*I*out_ is the most sensitive parameter for *iTPI* corresponding to the shift between the transient and sustained temporal pattern of serum insulin concentration.

## Discussion

We developed several alternative mathematical models using concentrations of plasma glucose, serum insulin, and C-peptide during consecutive hyperglycemic and hyperinsulinemic-euglycemic clamps, and selected the model showing the best fit for most subjects. Although *Model VI* was selected for 76 of 121 subjects, 45 subjects were not optimal for *Model VI*. This suggests that some of the parameters of *Model VI* were unnecessary in subjects whose selected model is *Model I*, *II*, *IV*, or *V* by comparing the structure with *Model VI*. However, no parameter of *Model VI* showed significant difference between subjects who selected *Model I*, *II*, *IV*, or *V* and subjects who selected *Model VI* (Supplementary Figure S7), suggesting that there is no biased feature on the structure of the control of circulating insulin concentration in subjects who were not optimal for *Model VI*, and *Model VI* can be applied to all subjects.

During the progression of glucose intolerance, it has been shown that the peak amplitude of circulating insulin concentration during the first-phase secretion decreases and the temporal pattern becomes more sustained.^2–4^ In this study, we found that both *k*_*I*out_, corresponding to peripheral insulin clearance, and *k*_ratio_ decrease from NGT to borderline type and T2DM. Given that (1 − *k*_ratio_), corresponding to hepatic insulin clearance, increases as the *k*_ratio_ decreases, our finding strongly suggests that, from NGT to borderline type and T2DM, the peak amplitude of serum insulin concentration decreases due to the increase in hepatic insulin clearance and the temporal pattern changes from transient to sustained occur because of a decrease in peripheral insulin clearance (Fig. 5). Importantly, the decrease in peripheral insulin clearance alone can explain only the temporal change of serum insulin concentration, not the decrease of peak amplitude. Thus, the increase of hepatic insulin clearance and decrease of peripheral insulin clearance simultaneously cause the decrease in the peak amplitude of serum insulin concentration during the first-phase secretion and change in temporal pattern from transient to sustained. Our result demonstrates that, in addition to the decrease in insulin secretion,^3,4^ the increase in hepatic clearance also contributes to the decrease in peak amplitude of serum insulin concentration in the first-phase secretion from NGT to borderline type and T2DM.

In our model, as *k*_ratio_, the ratio of post-hepatic insulin compared to C-peptide, increased, *ipeak*, the peak amplitude of peripheral insulin concentration, increased (Fig. 4c). According to clinical measurements, *k*_ratio_ was also correlated with *ipeak* calculated directly from the serum insulin concentration measured during the first-phase secretion in hyperglycemic clamp (Supplementary Figure S8a, *r* = 0.423, *P* < 0.001), indicating that hepatic insulin clearance is pathologically correlated with the peak amplitude in the first-phase secretion from NGT to borderline type and T2DM. On the other hand, in our model, as *k*_*I*out_, the peripheral insulin clearance, increased, *iTPI*, representing the temporal pattern of peripheral insulin concentration, increased (Fig. 4c). However, in clinical measurements, *k*_*I*out_ was not highly correlated with *iTPI* calculated directly from the serum insulin concentration measured during hyperglycemic clamp (Supplementary Figure S8a, *r* = 0.297, *P* < 0.01). The reason for this lack of correlation between *k*_*I*out_ and *iTPI* in clinical measurement remains unclear; however, it may be because little insulin was secreted during hyperglycemic clamp in some borderline type and T2DM subjects, and *iTPI* cannot be estimated accurately because of low concentration of serum insulin.

Many studies have shown that insulin clearance decreases in T2DM patients.^18–21,47,48^ However, the change in hepatic insulin clearance in this condition has been controversial, with some studies finding an increase in T2DM subjects^22^ and others a decrease.^18,23^ We previously developed a mathematical model using data gathered during hyperglycemic and hyperinsulinemic-euglycemic clamps, and peripheral insulin clearance significantly decreased from NGT to borderline type to T2DM.^30^ However, hepatic and peripheral insulin clearances were not estimated separately because we did not use C-peptide data. Recently, Polidori et al.^40^ estimated both hepatic and peripheral insulin clearance by modeling analysis using plasma insulin and C-peptide concentrations obtained from the insulin-modified frequently sampled IVGTT. They found that the peripheral insulin clearance significantly decreased in borderline type subjects compared with NGT subjects, whereas hepatic insulin clearance did not significantly differ between the borderline type and NGT subjects^40^; the former finding is consistent with our result that peripheral insulin clearance decreases from NGT to borderline type and T2DM. We demonstrated that hepatic insulin clearance significantly increases, whereas peripheral insulin clearance significantly decreases from NGT to borderline type and T2DM. One difference between the study by Polidori et al. and our study is C-peptide kinetics. They used the reported two-compartment model of C-peptide kinetics for calculating insulin secretion rate by deconvolution, while we selected the structure of C-peptide kinetics that fitted for our data, which may improve the accuracy of the parameter estimation of hepatic insulin clearance, estimated by use of serum insulin and C-peptide concentration. The increase in hepatic insulin clearance may be caused by impaired suppression of endocytosis of insulin receptors on the liver,^22^ and the decrease in peripheral insulin clearance may be caused by a decrease of the number of insulin receptors on target tissues.^45^ Polidori et al.^40^ also found that hepatic and peripheral insulin clearances were not highly correlated. Consistent with their results, in our analysis, the insulin clearance parameters *k*_ratio_ and *k*_*I*out_ were not highly correlated (Supplementary Figure S8b, *r* = 0.296, *P* < 0.01), suggesting that both insulin clearances are independently regulated.

We are aware that some of the results of this analysis only hold if the parameters are identifiable based on our serum insulin and C-peptide data. We performed 20 trials of parameter estimation for each subject (Methods), but most subjects (107 subjects) had only one trial which minimized RSS. The values of estimated parameters and RSS varied among the 20 trials of each subject. For the remaining four subjects, estimated parameters varied among trials that returned the same RSS, especially the parameters *α* and *β* differed to a large extent, while the parameters *k*_*I*out_ and *k*_ratio_ did not largely differ (Supplementary Figure S9). If the number of estimated trials, parents, and generations of evolutionary programming increases, a trial that gives a different parameter solution with smaller RSS than that reported in this study might be obtained. Structural or a priori identifiability of parameters based on the system equations,^49^ which tests if model parameters can be determined from the available data, was not performed in this study. Large variability in the fitted parameters, like for instance in *α* and *β*, could be due to the identifiability of the parameters and not due to biological variance, and interpretation of the results has to take this into account.

Insulin selectively regulates various functions, such as signaling activities, metabolic control, and gene expression, depending on its temporal patterns. For example, we previously reported that pulse stimulation of insulin in rat hepatoma Fao cells, resembling the first-phase secretion, selectively regulated glycogen synthase kinase-3β (GSK3β), which regulates glycogenesis, and S6 kinase, which regulates protein synthesis, whereas ramp stimulation of insulin, resembling the second-phase secretion, selectively regulated GSK3β and glucose-6-phosphatase (G6Pase), which regulates gluconeogenesis.^46^ We also found that insulin-dependent metabolic control and gene expression are selectively regulated by temporal patterns and doses of insulin in FAO cells.^50,51^ Sustained stimulation of insulin suppressed the expression of insulin receptors, leading to reduced insulin sensitivity in FAO cells.^52–54^ Likewise, phosphorylation of the insulin receptor substrate (IRS)-1/2 in rat liver increased when pulsatile (rather than continuous) stimulation of insulin was imposed in the portal circulation.^55^ This may have occurred through the negative feedback within the insulin signaling pathway, the phosphatidylinositide (PI) 3-kinase/Alt pathway, targeting IRS-1/2.^53,56^ In addition, IRS-2, rather than IRS-1, mainly regulates hepatic gluconeogenesis through its rapid downregulation by insulin,^57^ suggesting the selective roles of IRS-1/2 in response to temporal patterns of plasma insulin. These findings indicate that the amplitude and temporal pattern of circulating insulin concentration selectively regulate insulin actions on the target tissues. Given that hepatic and peripheral insulin clearances are responsible for the amplitude and temporal pattern of circulating insulin concentration, these clearances are likely to be involved in selective control of insulin action, glucose homeostasis, and the pathogenesis of T2DM.

We previously developed a mathematical model for concentrations of plasma glucose and serum insulin measured during consecutive hyperglycemic and hyperinsulinemic-euglycemic clamps and found significant decreases in insulin secretion, sensitivity, and peripheral insulin clearance from NGT to borderline type to T2DM.^30^ The differences between our previous study and this study are the model structure and C-peptide data. The previous model consisted of plasma glucose and serum insulin and required only glucose and insulin infusion as inputs. The model in this study does not have plasma glucose concentration but includes serum insulin and C-peptide concentrations, while plasma glucose concentration and insulin infusion are used as inputs (Fig. 2a, Supplementary Figure S2). In the previous study, only peripheral insulin clearance, but not hepatic insulin clearance, was estimated because C-peptide data were not used. The decrease of insulin clearance from NGT to T2DM in the previous study is consistent with the decrease of peripheral insulin clearance from NGT to T2DM in this study. In the previous study, the parameter corresponding to insulin secretion in the NGT and borderline type subjects was significantly higher than that in the T2DM subjects; however, the parameter related to insulin secretion did not show a significant difference between the NGT, borderline type, and T2DM subjects in this study, possibly because previously defined insulin secretion^30^ is described by insulin secretion and delivery in this model, and the parameters related to insulin secretion and delivery (*α*, *β*, *h*, *m*, *X*_b_, and *k*_ratio_) are too diversified. The parameter corresponding to insulin sensitivity was not incorporated in this study.

Many mathematical models to reproduce circulating C-peptide concentration have been developed. A two-compartment model for C-peptide kinetics was originally proposed.^31^ A combined model that included both circulating insulin and C-peptide kinetics described by a single compartment structure was introduced to estimate hepatic insulin clearance.^32^ The C-peptide minimal model describing peripheral insulin and C-peptide appearance and kinetics was also developed to assess hepatic insulin clearance,^33–37^ and several other model structures for circulating C-peptide concentration were reported.^38,39^ One difference between others’ and our studies is the experimental protocol in which data were applied to parameter estimation. IVGTT or hyperglycemic clamp were performed for parameter estimation in models of circulating C-peptide concentration, whereas we used hyperglycemic and hyperinsulinemic-euglycemic clamps, which may improve the accuracy of the parameter estimation of peripheral insulin clearance, *k*_*I*out_. Recently, a model of plasma insulin concentration including hepatic and peripheral insulin clearance and the delivery of insulin from the systemic circulation to the liver during the insulin-modified IVGTT was proposed.^40^ In that model, the parameter of hepatic insulin clearance was negatively correlated with acute insulin secretion in response to glucose, and the parameter of peripheral insulin clearance was correlated with insulin sensitivity,^40^ consistent with the results in this study (Fig. 3). Since the age of subjects in our study differed between groups with NGT, borderline type, and T2DM (Supplementary Table S4), the correlations between the parameters and clinical indices may be affected by age. However, the parameters showing the highest correlation with clinical indices of insulin secretion, AUC_IRI10_, insulin sensitivity, ISI, and insulin clearance, MCR, were not changed with conditioning of age (Supplementary Table S7). The high correlation between the parameter of hepatic insulin clearance, *k*_ratio_, and the clinical index of insulin secretion, AUC_IRI10_, suggests the possibility that hepatic insulin clearance considerably affects the clinical index of insulin secretion measured by peripheral insulin concentration because the clinical index of insulin secretion, AUC_IRI10_, was measured by the post-hepatic insulin delivery, and therefore reflects both insulin secretion and hepatic insulin clearance. This suggests that insulin secretion per se in the clinical index of insulin secretion may be overestimated because of the involvement of hepatic insulin clearance. Further study is necessary to address this issue.

In conclusion, using the mathematical model for serum insulin and C-peptide concentrations during consecutive hyperglycemic and hyperinsulinemic-euglycemic clamps, we determined the quantitative structure of the control of circulating insulin concentration. The estimated model parameters revealed the increase of hepatic insulin clearance and decrease of peripheral insulin clearance from NGT to borderline type and T2DM, and these changes selectively regulate the amplitude and temporal patterns of serum insulin concentration, respectively. The changes in opposite direction of both types of clearance shed light on the pathological mechanism underlying the abnormal temporal patterns of circulating insulin concentration from NGT to borderline type and T2DM.

## Materials and methods

### Subjects and measurements

The plasma and serum measurement data originated from our previous research.^14,30^ This metabolic analysis was approved by the ethics committee of Kobe University Hospital and was registerd with the University hospital Medical Information Network (UMIN000002359), and written informed consent was obtained from all subjects. In brief, 50 NGT, 18 borderline type, and 53 T2DM subjects underwent the consecutive clamp analyses. From 0 to 90 min, a hyperglycemic clamp was applied by intravenous infusion of a bolus of glucose (9622 mg/m^2^) within 15 min followed by that of a variable amount of glucose to maintain the plasma glucose level at 200 mg/dL. Ten minutes after the end of the hyperglycemic clamp, a 120-min hyperinsulinemic-euglycemic clamp was initiated by intravenous infusion of human regular insulin (Humulin R, Eli Lilly Japan K.K.) at a rate of 40 mU/m^2^/min and with a target plasma glucose level of 90 mg/dL. For the NGT and borderline type subjects whose plasma glucose levels were <90 mg/dL, the plasma glucose concentration was clamped at the fasting level. We measured the plasma glucose level every 1 min during the clamp analyses and obtained the 5-min average values. We also measured insulin and C-peptide level in serum samples collected at 5, 10, 15, 60, 75, 90, 100, 190, and 220 min after the onset of the tests. First-phase insulin secretion during the hyperglycemic clamp was defined as the incremental area under the immunoreactive insulin (IRI) concentration curve (μU/mL/min) from 0 to 10 min (AUC_IRI10_). The ISI derived from the hyperinsulinemic-euglycemic clamp was calculated by dividing the mean glucose infusion rate during the final 30 min of the clamp (mg/kg/min) by both the plasma glucose (mg/dL) and serum insulin (μU/mL) levels at the end of the clamp and then multiplying the result by 100. A clamp-based analog of the disposition index, the clamp disposition index (clamp DI), was calculated as the product of AUC_IRI10_ and ISI, as described previously.^14^ The MCR,^13^ an index of insulin clearance, was calculated by dividing the insulin infusion rate at the steady state (1.46 mU/kg/min) by the increase in insulin concentration above the basal level in the hyperinsulinemic-euglycemic clamp^14^: 1.46 (mU/kg/min) × body weight (kg) × body surface area (m^2^) / (end IRI − fasting IRI) (μU/mL), where body surface area is defined as (body weight (kg))^1/2^ × (body height (cm))^1/2^ / 60 (Mosteller formula). Since this study is a retrospective analysis of previously collected data, randomization and blinding of the groups with NGT, borderline type, and T2DM was not performed. The actual data for all 121 subjects are shown in Supplementary Figure S10 and Supplementary Table S8.

### Mathematical models

We developed six mathematical models based on the proposed models in order to choose the best model for reproducing our measurement of serum insulin and C-peptide during consecutive hyperglycemic and hyperinsulinemic-euglycemic clamps (Supplementary Figure S2). In these models, *I* represents serum insulin concentration (pM), and *CP* and *CP*_1_ represent serum C-peptide concentration (pM) including insulin and C-peptide secretion and hepatic and peripheral clearance. We used a conversion factor of insulin (6.00 nmol/U)^58^ and the molecular weights of glucose (180.16 g/mol) and C-peptide (3020.3 g/mol) to convert the unit of serum insulin, plasma glucose, and serum C-peptide, respectively. We used plasma glucose concentration *G* (mM) as input in the models, which was determined by stepwise interpolation of the measured plasma glucose data. Note that plasma glucose data were obtained as the 5-min average values, and each sampling time was reduced by 2 min in the calculation of stepwise interpolation.

The actual insulin infusion rate (IIR, mU/kg/min) was converted to the corresponding serum concentrations (cIIR) as follows:

7 $${\rm cIIR}\,\left( {{\mathrm{pM}}/{\mathrm{min}}} \right) = \frac{{{\rm IIR}\,({\mathrm{mU}}/{\mathrm{kg}}/{\mathrm{min}}) \cdot 6.00 \cdot 10^{ - 3}\,({\mathrm{pmol}}/{\mathrm{mU}})}}{{{\rm BV} \cdot 10^{ - 3}}},$$

where BV denotes blood volume (75 and 65 mL/kg for men and women, respectively^59^).

In the models, insulin infusions are represented by *influx*. This flux follows the nonlinear function *f* that predicts insulin infusion concentrations. Given that insulin infusion was performed only during the hyperinsulinemic-euglycemic (from 100 to 220 min) clamp, the function *f* was given by the following equations:

8 $$f(t) = \left\{ {\begin{array}{*{20}{c}} 0 & {(t \le 100)} \\ {ii_1 \cdot {\mathrm{exp}}(ii_2 \cdot (t - 100)) + ii_3} & {(t > 100)} \end{array}}, \right.$$

where the parameters *ii*_*j*_ (*j* = 1, 2, 3) are estimated to reproduce cIIR for each subject with a nonlinear least squares technique.^60^ Parameters for all subjects are shown in Supplementary Table S9.

### Parameter estimation

The model parameters for each subject were estimated to reproduce the experimentally measured time course by a meta-evolutionary programming method to approach the neighborhood of the local minimum, followed by application of the nonlinear least squares technique to reach the local minimum.^61^ Each parameter was estimated in the range from 10^−6^ to 10^4^. For these methods, the model parameters were estimated to minimize the objective function value, which is defined as the RSS between the actual time course obtained by clamp analyses and the model trajectories. RSS is given by:

9 $${\rm RSS} = \mathop {\sum}\limits_{{\mathrm{points}}} {\left\{ {\frac{{I(t) - I_{{\mathrm{sim}}}(t)}}{{I_{{\mathrm{mean}}}}}} \right\}^2} + \mathop {\sum}\limits_{{\mathrm{points}}} {\left\{ {\frac{{CP(t) - CP_{{\mathrm{sim}}}(t)}}{{CP_{{\mathrm{mean}}}}}} \right\}^2},$$

where

10 $$I_{{\mathrm{mean}}} = \frac{{\mathop {\sum}\limits_{{\mathrm{subjects}}} {\mathop {\sum}\limits_{{\mathrm{points}}} {I(t)} } }}{{\mathop {\sum}\limits_{{\mathrm{subjects}}} {{\mathrm{points}}} }},$$

11 $$CP_{{\mathrm{mean}}} = \frac{{\mathop {\sum}\limits_{{\mathrm{subjects}}} {\mathop {\sum}\limits_{{\mathrm{points}}} {CP(t)} } }}{{\mathop {\sum}\limits_{{\mathrm{subjects}}} {{\mathrm{points}}} }}.$$

*I*(*t*) and *CP*(*t*) are the serum insulin and C-peptide concentration, and *I*_sim_(*t*) and *CP*_sim_(*t*) are simulated serum insulin and C-peptide concentrations at *t* min, respectively. Serum insulin and C-peptide concentrations were normalized by dividing them by the averages of serum concentrations over all time points of all subjects of insulin (*I*_mean_, 302.7 pM) and C-peptide (*CP*_mean_, 1475 pM), respectively. The numbers of parents and generations in the meta-evolutionary programming were 400 and 4000, respectively. Parameter estimation was tried 20 times by changing the initial parameter values for each subject, and the parameter with the smallest RSS among 20 trials was taken as the estimated solution of each subject. Model parameters for all subjects are shown in Supplementary Table S9.

### Model selection

The model was chosen among the six models according to the AIC. For a given model and a single subject, AIC was calculated as follows:

12 $${\rm AIC} = n\,{\mathrm{ln}}\left( {\frac{{2\pi \cdot {\rm RSS}}}{n}} \right) + n + 2K,$$

where *n* is the total number of sampling time points of serum insulin and C-peptide, and *K* is the number of estimated parameters of the model.

### Determination of parameter outliers

The outliers of RSS and model parameters were detected by the adjusted outlyingness (AO).^62^ The cutoff value of AO was *Q*_3_ + 1.5*e*^3MC^ · IQR, where *Q*_3_, MC, and IQR are the third quartile, medcouple, and interquartile range, respectively. The medcouple is a robust measure of skewness.^63^ The number of directions was set at 8000. Subjects found to have outlier parameters (one NGT, one borderline type, and five T2DM subjects) were excluded from further study.

### Parameter sensitivity analysis

We defined the individual model parameter sensitivity for each subject as follows:

13 $$S(f(x),x) = \frac{{\partial \log f(x)}}{{\partial \log x}} = \frac{x}{{f(x)}} \cdot \frac{{\partial f(x)}}{{\partial x}},$$

where *x* is the parameter value and *f*(*x*) is *ipeak* or *iTPI*. The differentiation is numerically approximated by central difference $\frac{{\partial f(x)}}{{\partial x}} \approx \frac{{f(x + {\mathrm{\Delta }}x) - f(x - {\mathrm{\Delta }}x)}}{{{\mathrm{2\Delta }}x}}$, and *x* + Δ*x* and *x* − Δ*x* were set so as to be increased [*x* (1.1*x*)] or decreased [*x* (0.9*x*)] by 10%, respectively. Finally, we defined the parameter sensitivity by the median of the individual parameter sensitivity for all subjects. We examined the parameter sensitivity for six parameters of the rate constant related to serum insulin concentration, except *X*_b_ and *k*_*CP*out_. The higher the absolute value of parameter sensitivity, the larger the effect of the parameter on *ipeak* or *iTPI*.

### Statistical analysis

Unless indicated otherwise, data are expressed as the median with first and third quartiles. Medians of parameter values were compared among the NGT, borderline type, and T2DM subjects with the use of the two-sided Wilcoxon rank sum test with Benjamini Hochberg FDR multiple testing correction.^64^ An FDR-corrected *P* value <0.05 was considered statistically significant.

### Data availability

The SBToolbox2^65^ (http://www.sbtoolbox2.org/main.php) was used together with MATLAB R2015a (http://mathworks.com/) in the mathematical modeling. The code files implementing the 6 analyzed models and used in the simulation are freely available at http://kurodalab.bs.s.u-tokyo.ac.jp/info/.

## Electronic supplementary material

Supplementary Information

Supplementary Figure S6

Supplementary Table S1

Supplementary Table S3

Supplementary Table S6

Supplementary Table S8

Supplementary Table S9

## Electronic supplementary material

**Supplementary information** accompanies the paper on the *npj Systems Biology and Applications* website (10.1038/s41540-018-0051-6).
