# Supplementary material for: Increase in hepatic and decrease in peripheral insulin clearance characterize abnormal temporal patterns of serum insulin in diabetic subjects
Source: NPJ Syst Biol Appl. 2018 Mar 14;4:14. doi: 10.1038/s41540-018-0051-6 (PMC5852153; doi:10.1038/s41540-018-0051-6)
Supplement: Supplementary file 1 — Supplementary Information [file 41540_2018_51_MOESM1_ESM.docx]

**Supplementary Information**

**Figure S1.** Concentrations of plasma glucose, serum insulin and C-peptide, and insulin infusion during consecutive hyperglycemic and hyperinsulinemic-euglycemic clamps. Experimental (upper 4 panels) and simulation with *Model VI* (lower 2 panels) time courses are shown. Simulation time courses are plotted every 10 min. The mean ± SD among the subjects for NGT (green), borderline type (red), and T2DM (blue) with significant differences at each time point among the NGT, borderline type, and T2DM groups using two-sample *t*-test with FDR correction are depicted. **P* < 0.05; †*P* < 0.01, NGT vs. borderline type; ‡*P* < 0.05; §*P* < 0.01, NGT vs. T2DM; ||*P* < 0.05; ¶*P* < 0.01, borderline type vs. T2DM.

| **Model structure** | **Model equation** | **No. parameters** |
| --- | --- | --- |
| *Model I* | $\begin{matrix} v_{CP\text{in}}=CPS^{I}+CPS^{II} \\ CPS^{I}=k_{CP0}\delta(t) \\ CPS^{II}=\left\{\begin{matrix} \gamma(G-h)t \\ 0 \end{matrix}\right. \begin{matrix} (G>h) \\ (G\leq h) \end{matrix} \\ v_{I\text{out}}=k_{I\text{out}}(I-I_{\text{b}}) \\ v_{CP\text{out}}=k_{CP\text{out}}(CP-CP_{\text{b}}) \end{matrix}$ | 6  *k*_ratio_, *k_I_*_out_, *k_CP_*_out_, *k_CP_*_0_, *γ*, *h* |
| *Model II* | $\begin{matrix} v_{CP\text{in}}=CPS^{I}+CPS^{II} \\ CPS^{I}=k_{CP0}\delta(t) \\ CPS^{II}=\left\{\begin{matrix} \gamma(G-h)t \\ 0 \end{matrix}\right. \begin{matrix} (G>h) \\ (G\leq h) \end{matrix} \\ v_{I\text{out}}=k_{I\text{out}}(I-I_{\text{b}}) \\ v_{CP1CP2}=k_{12}(CP_{1}-CP_{\text{b}}) \\ v_{CP2CP1}=k_{21}CP_{2}, CP_{2}(0)=0 \\ v_{CP\text{out}}=k_{CP\text{out}}(CP_{1}-CP_{\text{b}}) \end{matrix}$ | 8  *k*_ratio_, *k_I_*_out_, *k_CP_*_out_, *k*_12_, *k*_21_, *k_CP_*_0_, *γ*, *h* |
| *Model III* | $\begin{matrix} \frac{dY}{dt}=\left\{\begin{matrix} \alpha\left\{\beta(G-h)-Y\right\} \\ -\alpha Y \end{matrix}\right. \begin{matrix} (G>h) \\ (G\leq h) \end{matrix}, Y(0)=0 \\ v_{CP\text{in}}=CPS^{III}+Y \\ CPS^{III}=\left\{\begin{matrix} k_{CP\text{d}}\cdot\frac{dG}{dt} \\ 0 \end{matrix} \right.\begin{matrix} \left(\frac{dG}{dt}>0\right) \\ \left(\frac{dG}{dt}\leq0\right) \end{matrix} \\ v_{I\text{out}}=k_{I\text{out}}(I-I_{\text{b}}) \\ v_{CP1CP2}=k_{12}(CP_{1}-CP_{\text{b}}) \\ v_{CP2CP1}=k_{21}CP_{2}, CP_{2}(0)=0 \\ v_{CP\text{out}}=k_{CP\text{out}}(CP_{1}-CP_{\text{b}}) \end{matrix}$ | 9  *k*_ratio_, *k_I_*_out_, *k_CP_*_out_, *k*_12_, *k*_21_, *k_CP_*_d_, *α*, *β*, *h* |
| *Model IV* | $\begin{matrix} \frac{dY}{dt}=\left\{\begin{matrix} \alpha\left\{\beta(G-h)-Y\right\} \\ -\alpha Y \end{matrix}\right. \begin{matrix} (G>h) \\ (G\leq h) \end{matrix}, Y(0)=0 \\ v_{CP\text{in}}=CPS^{III}+Y \\ CPS^{III}=\left\{\begin{matrix} k_{CP\text{d}}\cdot\frac{dG}{dt} \\ 0 \end{matrix} \right.\begin{matrix} \left(\frac{dG}{dt}>0\right) \\ \left(\frac{dG}{dt}\leq0\right) \end{matrix} \\ v_{I\text{out}}=k_{I\text{out}}(I-I_{\text{b}}) \\ v_{CP\text{out}}=k_{CP\text{out}}(CP-CP_{\text{b}}) \end{matrix}$ | 7  *k*_ratio_, *k_I_*_out_, *k_CP_*_out_, *k_CP_*_d_, *α*, *β*, *h* |
| *Model V* | $\begin{matrix} \frac{dY}{dt}=\left\{\begin{matrix} \alpha\left\{\beta(G-h)-Y\right\} \\ -\alpha Y \end{matrix}\right. \begin{matrix} (G>h) \\ (G\leq h) \end{matrix}, Y(0)=0 \\ v_{CP\text{in}}=\left\{\begin{matrix} m\cdot X \\ 0 \end{matrix} \right.\begin{matrix} (G>h) \\ (G\leq h) \end{matrix}, X(0)=X_{\text{b}} \\ v_{I\text{out}}=k_{I\text{out}}(I-I_{\text{b}}) \\ v_{CP1CP2}=k_{12}(CP_{1}-CP_{\text{b}}) \\ v_{CP2CP1}=k_{21}CP_{2}, CP_{2}(0)=0 \\ v_{CP\text{out}}=k_{CP\text{out}}(CP_{1}-CP_{\text{b}}) \end{matrix}$ | 10  *k*_ratio_, *k_I_*_out_, *k_CP_*_out_, *k*_12_, *k*_21_, *m*, *X*_b_, *α*, *β*, *h* |
| *Model VI* | $\begin{matrix} \frac{dY}{dt}=\left\{\begin{matrix} \alpha\left\{\beta(G-h)-Y\right\} \\ -\alpha Y \end{matrix}\right. \begin{matrix} (G>h) \\ (G\leq h) \end{matrix}, Y(0)=0 \\ v_{CP\text{in}}=\left\{\begin{matrix} m\cdot X \\ 0 \end{matrix} \right.\begin{matrix} (G>h) \\ (G\leq h) \end{matrix}, X(0)=X_{\text{b}} \\ v_{I\text{out}}=k_{I\text{out}}(I-I_{\text{b}}) \\ v_{CP\text{out}}=k_{CP\text{out}}(CP-CP_{\text{b}}) \end{matrix}$ | 8  *k*_ratio_, *k_I_*_out_, *k_CP_*_out_, *m*, *X*_b_, *α*, *β*, *h* |

**Figure S2.** Six mathematical models for serum insulin and C-peptide kinetics.

*Left panel*: The structure of models. *I* (pM) is serum insulin concentration, *CP* and *CP*_1_ (pM) are serum C-peptide concentration, *CP*_2_ (pM) is C-peptide in the extravascular compartment, and *X* (pM) is the amount of stored insulin and C-peptide. Insulin secretion and provision rate *Y* (pM min^–1^) are controlled by plasma glucose concentration *G* (mM). *Middle panel*: Mathematical representation of fluxes. Insulin infusion rate *influx* is shown in Methods. *Right panel*: The number of parameters for estimation, referred to as *K* in the calculation of AIC (Methods).

*Model I*. The model for serum insulin and C-peptide kinetics derived from the combined model ^1^. The model for insulin and C-peptide secretions (*CPS^I^* and *CPS^II^*) are from the insulin minimal model ^2^, instead of a cubic spline function in the combined model ^1^. This model has two variables, three fluxes, and six parameters, which is the simplest structure among the six models. The differential equations of this model are as follows:

| $\begin{matrix} \frac{dI}{dt}=k_{\text{ratio}}\cdot v_{CP\text{in}}-v_{I\text{out}}+influx \\ =\left\{\begin{matrix} k_{\text{ratio}}\cdot\left(k_{CP0}\delta(t)+\gamma(G-h)t\right)-k_{I\text{out}}\cdot\left(I-I_{\text{b}}\right)+f(t) \\ k_{\text{ratio}}\cdot k_{CP0}\delta(t)-k_{I\text{out}}\cdot\left(I-I_{\text{b}}\right)+f(t) \end{matrix} \begin{matrix} \left(G>h\right) \\ \left(G\leq h\right) \end{matrix}\right. \end{matrix}$ | (S1) |
| --- | --- |
| $\begin{matrix} \frac{dCP}{dt}=v_{CP\text{in}}-v_{CP\text{out}} \\ =\left\{\begin{matrix} k_{CP0}\delta(t)+\gamma(G-h)t-k_{CP\text{out}}\cdot\left(CP-CP_{\text{b}}\right) \\ k_{CP0}\delta(t)-k_{CP\text{out}}\cdot\left(CP-CP_{\text{b}}\right) \end{matrix} \begin{matrix} \left(G>h\right) \\ \left(G\leq h\right) \end{matrix}\right. \end{matrix}$ | (S2) |

Eq. S1 describes how serum insulin concentration *I* increases according to the post-hepatic insulin delivery *k*_ratio_ · *v_CP_*_in_, and decreases according to peripheral insulin clearance *v_I_*_out_. *I* also increases according to infused insulin *influx*. *v_CP_*_in_ is expanded as *k_CP_*_0_*δ*(*t*) + *γ*(*G* – *h*)*t* when *G* > *h*, otherwise *k_CP_*_0_*δ*(*t*), which corresponds to the sum of first-phase (*CPS^I^*) and second-phase (*CPS^II^*) secretion of insulin and C-peptide. The parameter *k_CP_*_0_ (pM) is the zero-intercept immediately after the start of hyperglycemic clamp, and *δ*(*t*) is the Dirac delta function approximated as follows:

|  | (S3) |
| --- | --- |

*k*_ratio_ · *v_CP_*_in_ indicates that only a fraction *k*_ratio_ of secreted insulin is delivered into peripheral circulation after passage through the liver when *G* > *h*. The parameter *k*_ratio_ is the molar ratio of post-hepatic insulin to C-peptide, which represents the fraction of insulin delivered to the peripheral circulation without being extracted by the liver. Given that C-peptide is not extracted by the liver, *k*_ratio_ can represent the remaining fraction of insulin after the extraction by the liver over the total amount of secreted insulin, and changes from 0 to 1. Therefore, (1 – *k*_ratio_) represents the fraction of insulin extracted by the liver and not delivered to the peripheral circulation, and corresponds to hepatic insulin clearance. *influx* is the insulin infusion rate during hyperinsulinemic-euglycemic clamp. The serum rate at time *t* is represented by *f*(*t*) (Methods). *v_I_*_out_ represents serum insulin degradation with the rate parameter *k_I_*_out_ (min^–1^). Therefore, *k_I_*_out_ represents insulin degradation in the periphery and corresponds to peripheral insulin clearance.

Eq. S2 describes how serum C-peptide concentration *CP* increases according to the C-peptide secretion *v_CP_*_in_, and decreases according to peripheral C-peptide clearance *v_CP_*_out_. *v_CP_*_in_ is C-peptide secretion and is delivered to peripheral circulation without hepatic clearance. *v_CP_*_out_ represents serum C-peptide degradation with the rate parameter *k_CP_*_out_ (min^–1^).

*Model II*. This model is identical to *Model I*, except the two-compartmental structure for the serum C-peptide kinetics ^3,4^, and has three variables, five fluxes, and eight parameters. The differential equations of *CP*_1_ and *CP*_2_ are as follows:

| $\begin{matrix} \frac{dCP_{1}}{dt}=v_{CP\text{in}}-v_{CP1CP2}+v_{CP2CP1}-v_{CP\text{out}} \\ =\left\{\begin{matrix} k_{CP0}\delta(t)+\gamma(G-h)t-k_{12}\left(CP_{1}-CP_{\text{b}}\right)+k_{21}CP_{2}-k_{CP\text{out}}\cdot\left(CP_{1}-CP_{\text{b}}\right) \\ k_{CP0}\delta(t)-k_{12}\left(CP_{1}-CP_{\text{b}}\right)+k_{21}CP_{2}-k_{CP\text{out}}\cdot\left(CP_{1}-CP_{\text{b}}\right) \end{matrix} \begin{matrix} \left(G>h\right) \\ \left(G\leq h\right) \end{matrix}\right. \end{matrix}$ | (S4) |
| --- | --- |
| $\begin{matrix} \frac{dCP_{2}}{dt}=v_{CP1CP2}-v_{CP2CP1} \\ =k_{12}\left(CP_{1}-CP_{\text{b}}\right)-k_{21}CP_{2} \end{matrix}$ | (S5) |

Eq. S4 describes how serum C-peptide concentration *CP*_1_ increases according to the C-peptide secretion *v_CP_*_in_ and transfer from extravascular compartment *v_CP_*_2_*_CP_*_1_, and decreases according to peripheral C-peptide clearance *v_CP_*_out t_ and transfer to extravascular compartment *v_CP_*_1_*_CP_*_2_. *v_CP_*_2_*_CP_*_1_ and *v_CP_*_1_*_CP_*_2_ represents C-peptide distribution from extravascular compartment to serum and vice versa, with the rate parameter *k*_21_ and *k*_12_ (min^–1^), respectively.

Eq. S5 describes how C-peptide in the extravascular compartment *CP*_2_ increases according to transfer from serum *v_CP_*_1_*_CP_*_2_, and decreases according to transfer to serum *v_CP_*_2_*_CP_*_1_.

*Model III*. The model for serum insulin and C-peptide kinetics is same as *Model II*, and the model for insulin and C-peptide secretions (*CPS^III^* and *Y*) are from another model ^5-7^. This model has four variables, seven fluxes, and nine parameters. The differential equations of *I*, *CP*_1_, and *Y* are as follows:

| $\begin{matrix} \frac{dI}{dt}=k_{\text{ratio}}\cdot v_{CP\text{in}}-v_{I\text{out}}+influx \\ =\left\{\begin{matrix} k_{\text{ratio}}\cdot\left(k_{CP\text{d}}\cdot\frac{dG}{dt}+Y\right)-k_{I\text{out}}\cdot\left(I-I_{\text{b}}\right)+f(t) \\ k_{\text{ratio}}\cdot Y-k_{I\text{out}}\cdot\left(I-I_{\text{b}}\right)+f(t) \end{matrix} \begin{matrix} \left(\frac{dG}{dt}>0\right) \\ \left(\frac{dG}{dt}\leq0\right) \end{matrix}\right. \end{matrix}$ | (S6) |
| --- | --- |
| $\begin{matrix} \frac{dCP_{1}}{dt}=v_{CP\text{in}}-v_{CP1CP2}+v_{CP2CP1}-v_{CP\text{out}} \\ =\left\{\begin{matrix} k_{CP\text{d}}\cdot\frac{dG}{dt}+Y-k_{12}\left(CP_{1}-CP_{\text{b}}\right)+k_{21}CP_{2}-k_{CP\text{out}}\cdot\left(CP_{1}-CP_{\text{b}}\right) \\ Y-k_{12}\left(CP_{1}-CP_{\text{b}}\right)+k_{21}CP_{2}-k_{CP\text{out}}\cdot\left(CP_{1}-CP_{\text{b}}\right) \end{matrix} \begin{matrix} \left(\frac{dG}{dt}>0\right) \\ \left(\frac{dG}{dt}\leq0\right) \end{matrix}\right. \end{matrix}$ | (S7) |
|  | (1) |

Eq. S6 and S7 describe the changes of serum insulin and C-peptide concentration similar to Eq. S1 and S4, respectively, except that *v_CP_*_in_ is expanded as *k_CPd_* · *dG*/*dt* + *Y* when *dG*/*dt* > 0, otherwise *Y*, which corresponds to the sum of insulin and C-peptide secretion controlled by the rate of change of plasma glucose concentration (*CPS^III^*) and by glucose concentration (*Y*). The parameter *k_CPd_* describes the effect of the rate of change of glucose on insulin secretion when glucose concentration is increasing.

Eq. 1 describes how insulin provision rate *Y* increases according to *αβ*(*G – h*) when *G* > *h*, and decreases with *αY*. This means that provision of insulin and C-peptide tends with a time constant 1/*α* (min) toward a steady-state value linearly related via parameter *β* (min^–1^) to plasma glucose concentration *G* (mM) above its basal level *h*. See also Eq. 1 in Results.

*Model IV*. This model is identical to *Model III*, except the one-compartmental structure for the serum C-peptide kinetics is similar to *Model I*, and has three variables, five fluxes, and seven parameters. The differential equations of *CP* are as follows:

| $\begin{matrix} \frac{dCP}{dt}=v_{CP\text{in}}-v_{CP\text{out}} \\ =\left\{\begin{matrix} k_{CP\text{d}}\cdot\frac{dG}{dt}+Y-k_{CP\text{out}}\cdot\left(CP-CP_{\text{b}}\right) \\ Y-k_{CP\text{out}}\cdot\left(CP-CP_{\text{b}}\right) \end{matrix} \begin{matrix} \left(\frac{dG}{dt}>0\right) \\ \left(\frac{dG}{dt}\leq0\right) \end{matrix}\right. \end{matrix}$ | (S8) |
| --- | --- |

Eq. S8 describes the change of serum C-peptide concentration similar to Eq. S2, except that *v_CP_*_in_ is expanded as *k_CPd_* · *dG*/*dt* + *Y* when *dG*/*dt* > 0, otherwise *Y*, similarly described in Eq. S7.

*Model V*. The model for serum insulin and C-peptide kinetics is same as *Model II*, and the model for insulin and C-peptide secretions (*X* and *Y*) are from the C-peptide minimal model ^5,8^. This model has five variables, eight fluxes, and 10 parameters. The differential equations of *I*, *CP*_1_, and *X* are as follows:

| $\begin{matrix} \frac{dI}{dt}=k_{\text{ratio}}\cdot v_{CP\text{in}}-v_{I\text{out}}+influx \\ =\left\{\begin{matrix} k_{\text{ratio}}\cdot m\cdot X-k_{I\text{out}}\cdot\left(I-I_{\text{b}}\right)+f(t) \\ -k_{I\text{out}}\cdot\left(I-I_{\text{b}}\right)+f(t) \end{matrix} \begin{matrix} \left(G>h\right) \\ \left(G\leq h\right) \end{matrix}\right. \end{matrix}$ | (3) |
| --- | --- |
| $\begin{matrix} \frac{dCP_{1}}{dt}=v_{CP\text{in}}-v_{CP1CP2}+v_{CP2CP1}-v_{CP\text{out}} \\ =\left\{\begin{matrix} m\cdot X-k_{12}\left(CP_{1}-CP_{\text{b}}\right)+k_{21}CP_{2}-k_{CP\text{out}}\cdot\left(CP_{1}-CP_{\text{b}}\right) \\ -k_{12}\left(CP_{1}-CP_{\text{b}}\right)+k_{21}CP_{2}-k_{CP\text{out}}\cdot\left(CP_{1}-CP_{\text{b}}\right) \end{matrix} \begin{matrix} \left(G>h\right) \\ \left(G\leq h\right) \end{matrix}\right. \end{matrix}$ | (S9) |
| $\frac{dX}{dt}=Y-v_{CP\text{in}}=\left\{\begin{matrix} Y-m\cdot X \\ Y \end{matrix} \begin{matrix} \left(G>h\right) \\ \left(G\leq h\right) \end{matrix}\right.$ | (2) |

Eq. 3 and S9 describe the change of serum insulin and C-peptide concentration similar to Eq. S1 and S4, except that *v_CP_*_in_ is expanded as *m* · *X*, which corresponds to insulin and C-peptide secretion when *G* > *h*.

Eq. 2 describes that *X* (pM) increases according to the provision rate *Y* (pM min^–1^) and decreases according to the insulin and C-peptide secretion *v_CP_*_in_. *v_CP_*_in_ is *X* secreted at the rate *m* (min^–1^) when *G* > *h*. The initial value of *X*, *X*_b_ (pM), is the parameter and is responsible for the first-phase secretion, whereas the slower second-phase secretion derives from provision *Y*. See also Eq. 2 and 3 in Results.

*Model VI*. This model is identical to *Model V*, except the one-compartmental structure for the serum C-peptide kinetics is similar to *Model I*, and has four variables, six fluxes, and eight parameters. The differential equations of *CP* are as follows:

| $\begin{matrix} \frac{dCP}{dt}=v_{CP\text{in}}-v_{CP\text{out}} \\ =\left\{\begin{matrix} m\cdot X-k_{CP\text{out}}\cdot\left(CP-CP_{\text{b}}\right) \\ -k_{CP\text{out}}\cdot\left(CP-CP_{\text{b}}\right) \end{matrix} \begin{matrix} \left(G>h\right) \\ \left(G\leq h\right) \end{matrix}\right. \end{matrix}$ | (4) |
| --- | --- |

Eq. 4 describes the change of serum C-peptide concentration similar to Eq. S2, except that *v_CP_*_in_ is expanded as *m* · *X*, which corresponds to insulin and C-peptide secretion when *G* > *h*, similarly described in Eq. S9.

In this study, we performed analyses using *Model VI* (Fig. 2a, Table 1).

**Figure S3.** The distribution of residual sum of squares (RSS) between time courses of insulin and C-peptide reproduced by *Model VI* and serum measurement. Relative frequency histograms of 76 subjects who were optimal for *Model VI* (gray) and of 45 subjects who were not optimal for *Model VI* (yellow, pink, blue, and green indicate subjects who were optimal for *Models I*, *II*, *IV*, and *V*, respectively) are shown. The bin size of each histogram is 0.25. These two RSS distributions were not significantly different (Kolmogorov-Smirnov test, *P* = 0.118). The subjects with upper three RSS values (#104: T2DM, RSS = 4.42, #6: NGT, RSS = 3.58, #26: NGT, RSS = 1.96) were excluded as outliers of the RSS distribution of all 121 subjects (adjusted outlyingness, see Methods).

**a**

**b**

**Figure S4.** The relationship between the clusters of time courses of serum insulin and C-peptide concentration and selected models.

(**a**) Hierarchical clustering of time courses of serum insulin and C-peptide concentration for the remaining 118 subjects resulted in six clusters. Normalized serum insulin and C-peptide concentration (see Methods) measured over 10 time points (0–220 min) for each subject were shown in the heat map. The analysis was performed using the Ward hierarchical clustering technique with Euclidean distance, and the hierarchy was cut at 0.25 times the maximum height. Two color bars on the right side of the heat map indicate the categorized stage of each subject (NGT, borderline type, or T2DM) and the optimal model for the subject. Pie charts show the proportion of the number of subjects optimal for each model among the subjects classified in each cluster.

*Cluster 1*: Serum insulin and C-peptide concentrations increase during both first-phase (0–15 min) and second-phase secretion (15–90 min) under hyperglycemic clamp, and serum C-peptide concentration returns to the fasting level during hyperinsulinemic-euglycemic clamp. The subjects in this cluster were optimal for *Model I*, *IV*, and *VI*.

*Cluster 2*: Serum insulin and C-peptide concentrations increase during the first-phase secretion, but do not increase much during the second-phase secretion. Most subjects in this cluster were in the NGT group and optimal for *Model VI*.

*Cluster 3*: Serum insulin and C-peptide concentrations do not increase much during the first-phase secretion, but increase during the second-phase secretion. Half the subjects in this cluster were in the NGT group and the others were in the borderline type group, and half of the subjects were optimal for *Model VI*.

*Cluster 4*: Serum insulin and C-peptide concentrations do not increase during both the first- and second-phase secretion. Most subjects in this cluster were in the T2DM group and optimal for *Model VI*.

*Cluster 5*: Serum insulin and C-peptide concentrations increase during both first- and second-phase secretions, and serum insulin concentration during hyperinsulinemic-euglycemic clamp was relatively low compared to that of the subjects in the other clusters. Most subjects in this cluster were in the NGT group and optimal for *Model VI*.

*Cluster 6*: Serum insulin and C-peptide concentrations moderately increase during both first- and second-phase secretions. NGT, borderline type, and T2DM subjects were included in this cluster, and most subjects were optimal for *Model VI*.

As described above, the cluster of time course of serum insulin and C-peptide concentrations corresponds to the stage of progression of T2DM of the subjects in the cluster to some extent. NGT, borderline type, and T2DM subjects account for the majority of *Clusters 2* and *5*, *Cluster 3*, and *Cluster 4*, respectively. NGT, borderline type, and T2DM subjects are included in *Cluster 6*, suggesting that it is possible to classify subjects independent of the stage of progression of glucose intolerance. However, optical models for the subjects were not significantly different among clusters in which the subjects were classified (Supplementary Table S2).

(**b**) Normalized time courses of serum insulin and C-peptide concentration of NGT (green), borderline type (red), and T2DM (blue) subjects classified in each cluster (column) and optimal for each model (row) are shown. Each panel in the leftmost column shows the time courses of subjects in each cluster, and each panel in the top row shows the time courses of subjects optimal for each model. The other panels show time courses of subjects in the cluster of the row and optimal for the model of the column.

**Figure S5.** Scatter plots of *k_I_*_out_, ISI, and MCR. The partial correlation coefficient, *ρ*_X,Y|Z_, defined as the correlation between X and Y conditioning of Z, is shown. Model parameter *k_I_*_out_ shows the correlations with clinical indices after removing the effect of the other clinical index.

**Figure S6.** The roles of *k*_ratio_ and *k_I_*_out_ in the amplitude and temporal patterns of serum insulin concentration for each subject.

**Figure S7.** The estimated parameters of *Model VI* for subjects who selected each of Model *I*, *II*, *IV*, *V*, and *VI.*

(**a**) Medians and upper and lower quartiles of 76 subjects who were optimal for *Model VI* (gray) and of 45 subjects who were not optimal for *Model VI* (yellow, pink, blue, and green indicate subjects who were optimal for *Models I*, *II*, *IV*, and *V*, respectively) are shown. Each dot corresponds to the indicated parameter for an individual subject.

(**b**) Box plots of 76 subjects who were optimal for *Model VI* (gray) and of 45 subjects who were not optimal for *Model VI* are shown. Note that all graphs use a log-10 scale for the Y axis.

No parameter differed significantly between subjects who were optimal for each of *Models I*, *II*, *IV*, and *V* and subjects who were optimal for *Model VI*, using two-sided Wilcoxon rank sum test (36 tests were performed, FDR-corrected *P* value > 0.05).

**Figure S8.** Distribution of model parameters and indices of peak amplitude and temporal pattern of serum insulin concentration. (**a**) Scatter plots of *ipeak* and *k*_ratio_ and of *iTPI* and *k_I_*_out_. *ipeak* and *iTPI* are calculated directly from the measured serum insulin concentration. (**b**) Scatter plot of *k_I_*_out_ and *k*_ratio_.

**Figure S9.** The estimated parameters of *Model VI* for subjects who had two or more trials which minimized RSS (#41: NGT, #83: T2DM, #90: T2DM, #115: T2DM). Each dot corresponds to the indicated parameter of an individucal trial of an individual subject in linear (**a**) and log-10 (**b**) scale. The parameters indicated by triangle dots were used in this study.

**Figure S10.** Concentrations of plasma glucose, serum insulin, and C-peptide for each subject. Red dots, experimental measurement; blue curves, the simulated time courses; black lines, plasma glucose concentration in the linear interpolation. Subjects #1 to #50 are NGT (green background), #51 to #68 are borderline type (red background), and #69 to #121 are T2DM (blue background). Subjects shown with an orange background (two NGT and one T2DM) were excluded from further analysis as outliers of residual sum of squares (RSS; see Supplementary Figure S3), and subjects shown with a gray background (one NGT, one borderline type, and five T2DM) were excluded from further analysis as outliers of parameter values. The RSS between the time course for the clamp and the model trajectory is shown for each subject. The distribution of RSS for all subjects is shown in Supplementary Figure S3.

**Table S1.** Significant differences of plasma and serum concentrations and insulin infusion at each time point among NGT, borderline type, and T2DM groups

*P* values were calculated when comparing average plasma and serum measurements among these groups using two-sample *t*-test with FDR correction. C-P(*t*), C-IRI(*t*), C-CPR(*t*), and C-IIR(*t*) are measured concentrations of plasma glucose, serum insulin and C-peptide, and insulin infusion at time *t*, respectively. The highlighted cells represent *P* < 0.05, and the color becomes darker as the *P* value decreases.

**Table S2.** AIC of each model calculated using measured time courses of all 121 subjects

| Model | RSS | AIC | No. parameters |
| --- | --- | --- | --- |
| *I* | 49.0 | -1050 | 726 |
| *II* | 50.9 | -479 | 968 |
| *III* | 53.2 | -129 | 1089 |
| *IV* | 61.4 | -272 | 847 |
| *V* | 29.2 | -1320 | 1210 |
| *VI* | 31.8 | -1600 | 968 |

RSS between the actual time course obtained by clamp analyses and the model trajectories of all 121 subjects is given by summing RSS (Eq. 9) for all subjects.

| $RSS=\sum_{subjects} \left[\sum_{\text{points}} \left\{\frac{I(t)-I_{\text{sim}}(t)}{I_{\text{mean}}}\right\}^{2}+\sum_{\text{points}} \left\{\frac{CP(t)-CP_{\text{sim}}(t)}{CP_{\text{mean}}}\right\}^{2}\right]$ | (S9) |
| --- | --- |

AIC was calculated according to Eq. 12, where *n* is the total number of sampling time points of serum insulin and C-peptide for all subjects, and *K* is the number of estimated parameters of the model for all subjects as shown.

**Table S3.** Significant differences in optimal models for the subjects among clusters

The number of subjects who are classified or not classified in the cluster, those who were optimal or not optimal for the model, and *P* values for comparison using Fisher exact test are shown. *P* values are corrected by the number of tests, multiplied by 30.

**Table S4.** Characteristics of the three groups of study subjects

|  | NGT | Borderline | T2DM | Total |
| --- | --- | --- | --- | --- |
| Number | 47 | 17 | 47 | 111 |
| Sex (male/female) | 21/26 | 10/7 | 31/16 | 62/49 |
| Age (years) | 30.3 ± 8.68 | 42.0 ± 12.0 | 56.1 ± 12.7 | 43.0 ± 16.2 |
| BMI (kg/m^2^) | 21.2 ± 3.47 | 26.9 ± 6.80 | 25.9 ± 5.03 | 24.1 ± 5.34 |
| FPG (mg/dL) | 85.4 ± 6.79 | 91.8 ± 14.7 | 111 ± 23.5 | 97.2 ± 20.7 |
| 2-h PG (mg/dL) | 111 ± 17.2 | 167 ± 16.1 | 264 ± 76.9 | 184 ± 87.3 |
| F-IRI (μU/mL) | 5.44 ± 2.10 | 9.18 ± 5.63 | 6.65 ± 4.25 | 6.53 ± 3.95 |
| F-CPR (ng/mL) | 1.45 ± 0.394 | 2.13 ± 0.753 | 1.83 ± 0.872 | 1.71 ± 0.725 |

Data are means ± SD. BMI, body mass index; FPG, fasting plasma glucose concentration; 2-h PG, 2-h plasma glucose level during the oral glucose tolerance test; F-IRI, fasting serum immunoreactive insulin concentration; F-CPR, fasting serum immunoreactive C-peptide concentration.

**Table S5.** Statistic power of the two-sided Wilcoxon rank sum test for parameters.

| Parameter | Group_1_ | Group_2_ | *μ*_1_ | *μ*_2_ | *σ*_1_ | *σ*_2_ | *d* | *N*_1_ | *N*_2_ | 1 - *β* |
| --- | --- | --- | --- | --- | --- | --- | --- | --- | --- | --- |
| *k_I_*_out_ | NGT | Borderline | 0.245 | 0.168 | 0.0697 | 0.0324 | 1.10 | 47 | 17 | 0.875 |
|  | NGT | T2DM | 0.245 | 0.193 | 0.0697 | 0.0475 | 0.746 | 47 | 47 | 0.828 |
| *k*_ratio_ | NGT | Borderline | 0.599 | 0.446 | 0.192 | 0.128 | 0.797 | 47 | 17 | 0.535 |
|  | NGT | T2DM | 0.599 | 0.354 | 0.192 | 0.256 | 1.28 | 47 | 17 | 1.00 |
| *h* | NGT | T2DM | 4.40 | 5.68 | 1.14 | 1.80 | 0.711 | 47 | 47 | 0.782 |
| *X*_b_ | NGT | T2DM | 1550 | 2190 | 1400 | 3270 | 0.196 | 47 | 47 | 0.0545 |

Statistical power level 1 - *β* of each parameter which significantly differ between the NGT, borderline type, and T2DM groups (Figure 2) was calculated by use of G*Power 3.1 ^9^. Since two-sided Wilcoxon rank sum test does not depend on the specific form of the response distribution of each parameter of the group, we defined that the response distribution is normal distribution. The significance level α is defined as 0.00608, which is the largest uncorrected *P* value among the FDR-corrected *P* value <0.05 (Figure 2 and Methods). *μ*_k_, mean of the parameter; *σ*_k_, standard deviation of the parameter; *N*_k_, number of subjects of the Group_k_. The effect size *d* proposed by Cohen ^10^ is calculated as:

| $d=\frac{\left\vert\mu_{1}-\mu_{2}\right\vert}{\sigma}$ | (S10) |
| --- | --- |

, where *σ* is the larger between *σ*_1_ and *σ*_2_.

**Table S6.** Correlation between the model parameters and measured clinical indices

Correlation coefficients (*r*) for the model parameters versus the indicated clinical indices (see Methods) are listed in descending order of absolute value. *P* values are for testing the hypothesis of no correlation (corrected by the number of correlation calculations, multiplied by 54).

**Table S7.** Partial correlation between the model parameters and measured clinical indices

| Rank | ISI | | MCR | | AUC_IRI10_ | |
| --- | --- | --- | --- | --- | --- | --- |
|  |  | *ρ* |  | *ρ* |  | *ρ* |
| 1 | *k_I_*_out_ | 0.728 | *k_I_*_out_ | 0.778 | *k*_ratio_ | 0.287 |
| 2 | *k*_ratio_·*k_I_*_out_ | 0.633 | *k*_ratio_·*k_I_*_out_ | 0.516 | *k_I_*_out_ | -0.202 |
| 3 | *k*_ratio_ | 0.343 | *k*_ratio_ | 0.187 | *α* | -0.151 |
| 4 | *α* | -0.146 | *X*_b_ | 0.0527 | *k_CP_*_out_ | -0.126 |
| 5 | *β* | -0.0734 | *β* | -0.0518 | *X*_b_ | 0.101 |
| 6 | *k_CP_*_out_ | -0.0555 | *m* | 0.0275 | *k*_ratio_·*k_I_*_out_ | 0.0941 |
| 7 | *X*_b_ | -0.0554 | *α* | 0.0239 | *h* | -0.0845 |
| 8 | *m* | -0.0156 | *k_CP_*_out_ | 0.00347 | *m* | -0.0699 |
| 9 | *h* | 0.00799 | *h* | 0.000521 | *β* | -0.0637 |

The partial correlation coefficient (*ρ*), defined as the correlation between the model parameters and measured clinical indices conditioning of age, are listed in descending order of absolute value.

**Table S8.** Experimental measurements for each subject

Those shown with an orange background (two NGT and one T2DM) were excluded from further analysis as outliers of residual sum of squares (RSS; see Supplementary Figure S3), and subjects shown with a gray background (one NGT, one borderline type, and five T2DM) were excluded from further analysis as outliers of parameter values (see Methods).

**Table S9.** The estimated model parameters for each subject

Subjects shown with an orange background (two NGT and one T2DM) were excluded from further analysis as outliers of residual sum of squares (RSS; see Supplementary Figure S3), and subjects shown with a gray background (one NGT, one borderline type, and five T2DM) were excluded from further analysis as outliers (see Methods).

**Supplementary References**

1 Vølund, A., Polonsky, K. S. & Bergman, R. N. Calculated pattern of intraportal insulin appearance without independent assessment of C-peptide kinetics. *Diabetes* **36**, 1195-1202 (1987).

2 Bergman, R. N., Phillips, L. S. & Cobelli, C. Physiologic evaluation of factors controlling glucose tolerance in man: measurement of insulin sensitivity and beta-cell glucose sensitivity from the response to intravenous glucose. *J Clin Invest* **68**, 1456-1467 (1981).

3 Eaton, R. P., Allen, R. C., Schade, D. S., Erickson, K. M. & Standefer, J. Prehepatic insulin production in man: kinetic analysis using peripheral connecting peptide behavior. *J Clin Endocrinol Metab* **51**, 520-528, doi:10.1210/jcem-51-3-520 (1980).

4 Cobelli, C. & Pacini, G. Insulin secretion and hepatic extraction in humans by minimal modeling of C-peptide and insulin kinetics. *Diabetes* **37**, 223-231 (1988).

5 Grodsky, G. M. A threshold distribution hypothesis for packet storage of insulin and its mathematical modeling. *J Clin Invest* **51**, 2047-2059, doi:10.1172/JCI107011 (1972).

6 Ličko, V. & Silvers, A. Open-loop glucose-insulin control with threshold secretory mechanism: Analysis of intravenous glucose tolerance tests in man. *Mathematical Biosciences* **27**, 319-332, doi:http://dx.doi.org/10.1016/0025-5564(75)90110-8 (1975).

7 Toffolo, G. *et al.* Quantitative indexes of beta-cell function during graded up&down glucose infusion from C-peptide minimal models. *Am J Physiol Endocrinol Metab* **280**, E2-10 (2001).

8 Toffolo, G., Campioni, M., Basu, R., Rizza, R. A. & Cobelli, C. A minimal model of insulin secretion and kinetics to assess hepatic insulin extraction. *Am J Physiol Endocrinol Metab* **290**, E169-E176, doi:10.1152/ajpendo.00473.2004 (2006).

9 Faul, F., Erdfelder, E., Lang, A.-G. & Buchner, A. G*Power 3: A flexible statistical power analysis program for the social, behavioral, and biomedical sciences. *Behavior Research Methods* **39**, 175-191 (2007).

10 Cohen, J. *Statistical Power Analysis for the Behavioral Sciences*. (Academic Press, 1969).
