# Supplementary figures and images for: Increase in hepatic and decrease in peripheral insulin clearance characterize abnormal temporal patterns of serum insulin in diabetic subjects
Source: NPJ Syst Biol Appl. 2018 Mar 14;4:14. doi: 10.1038/s41540-018-0051-6 (PMC5852153; doi:10.1038/s41540-018-0051-6)

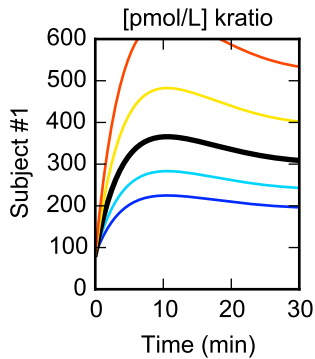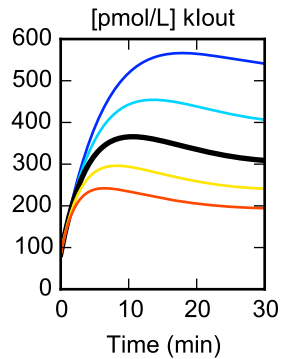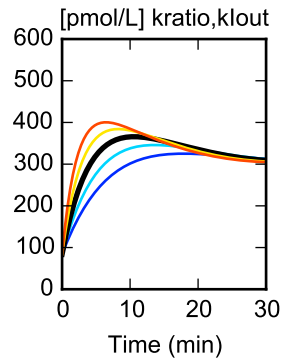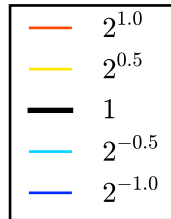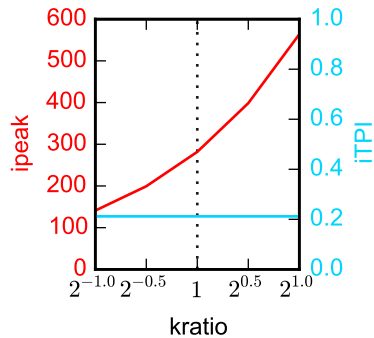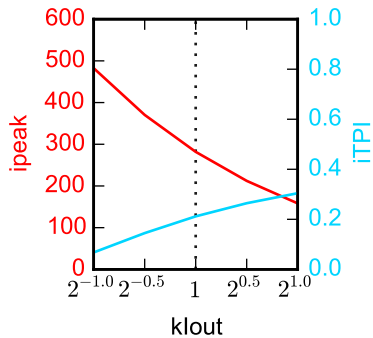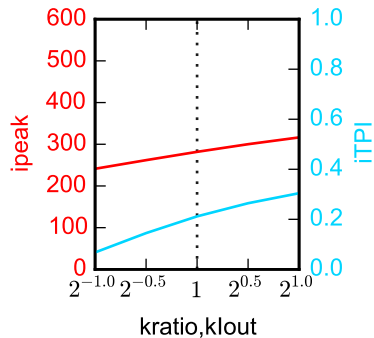

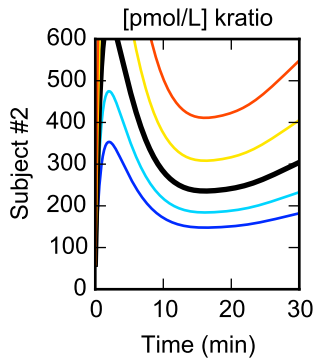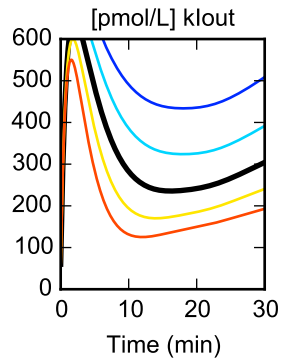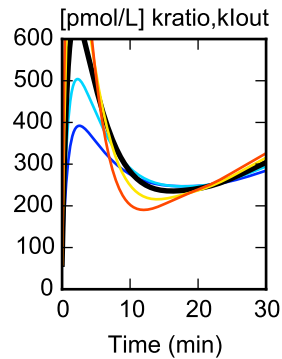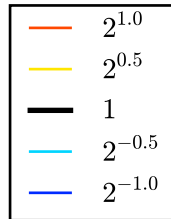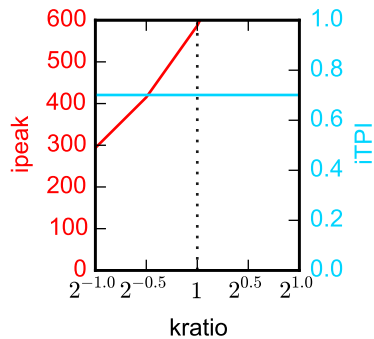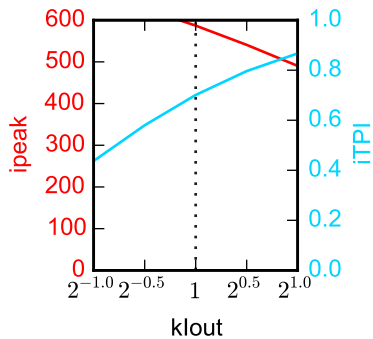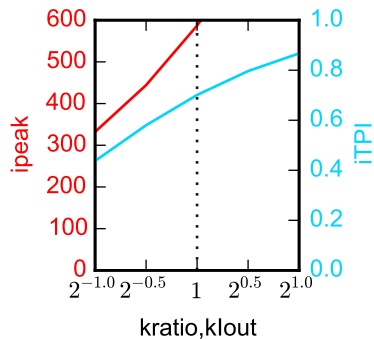

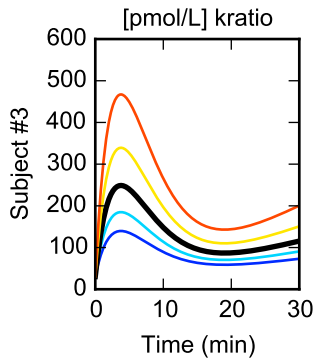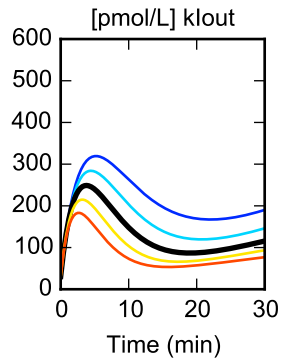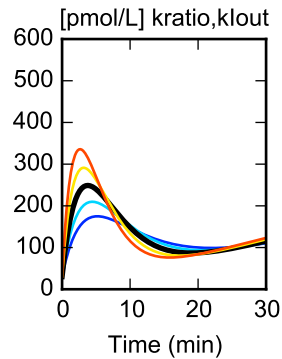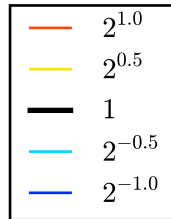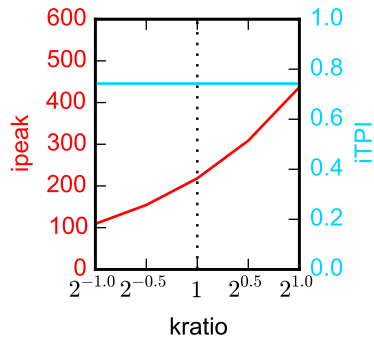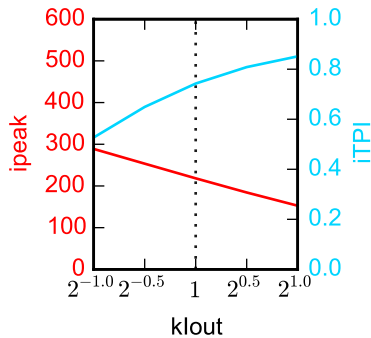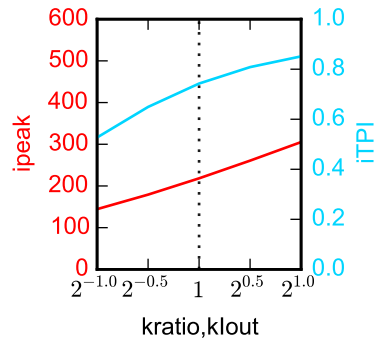

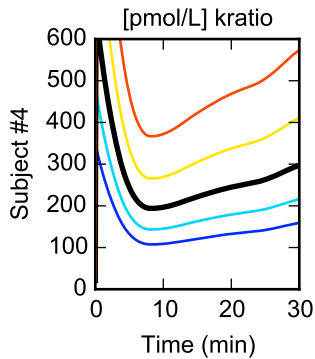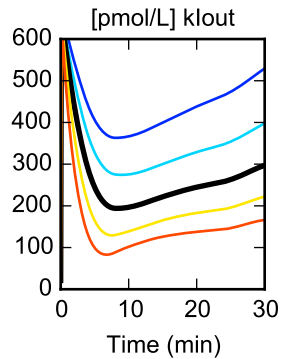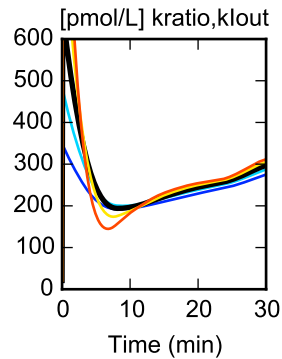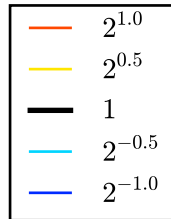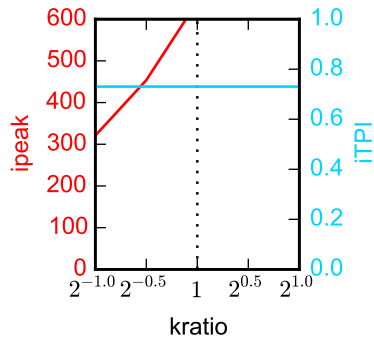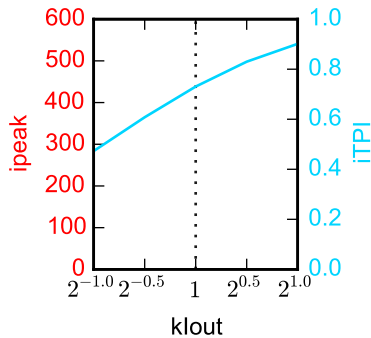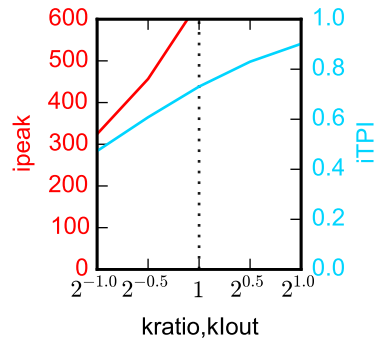

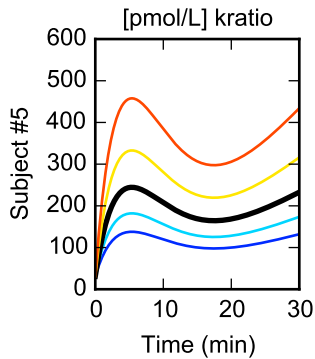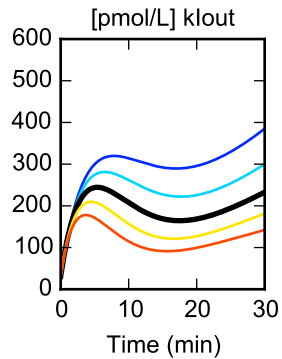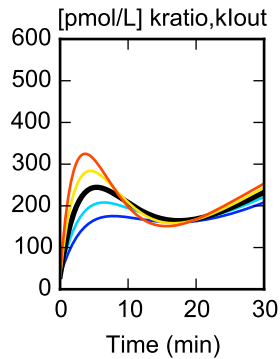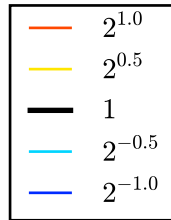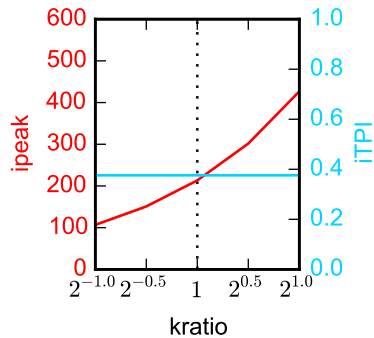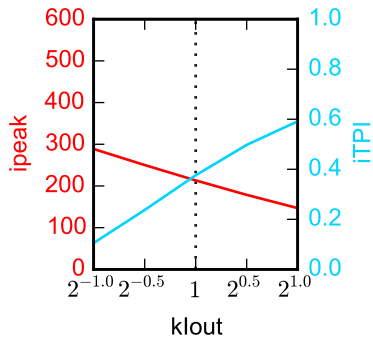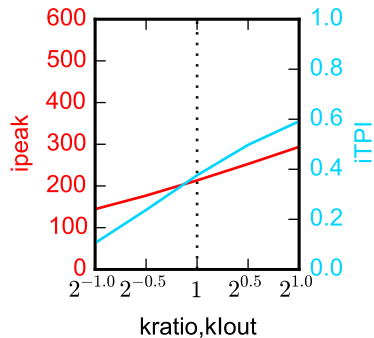

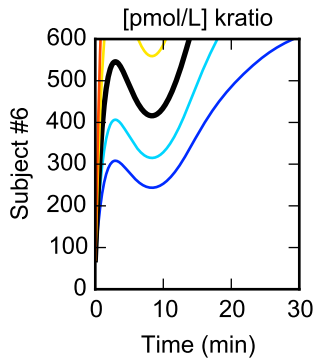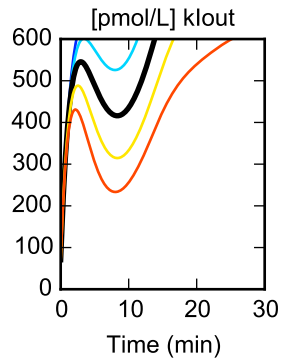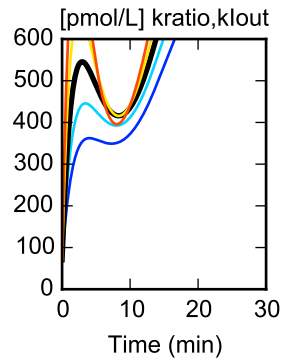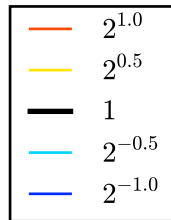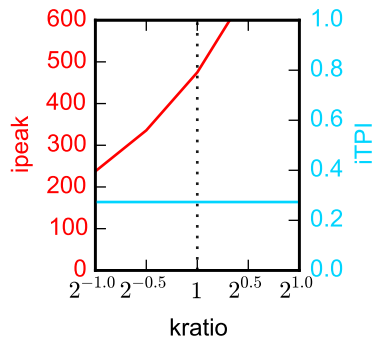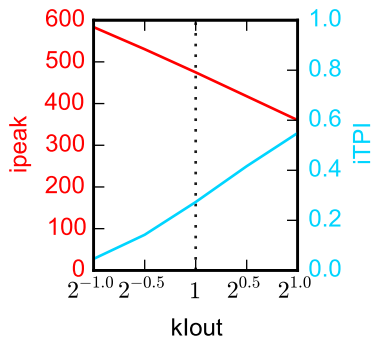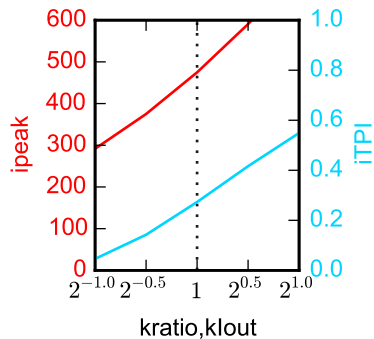

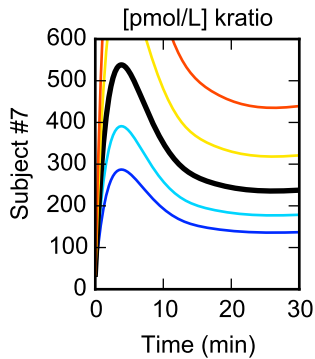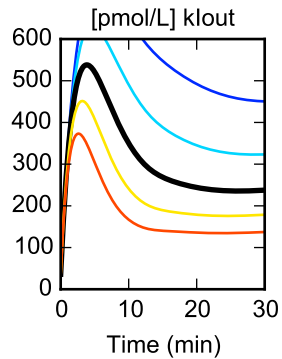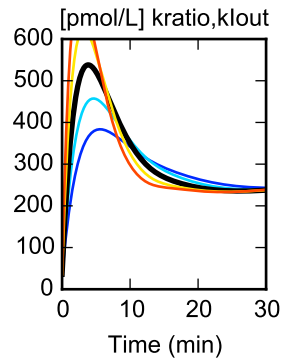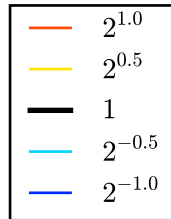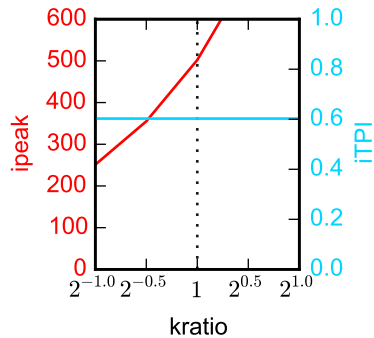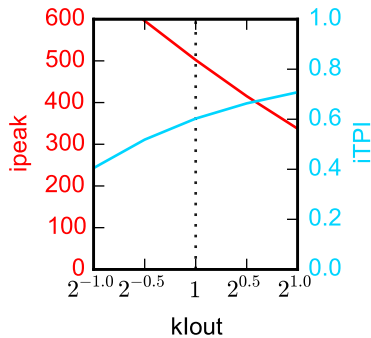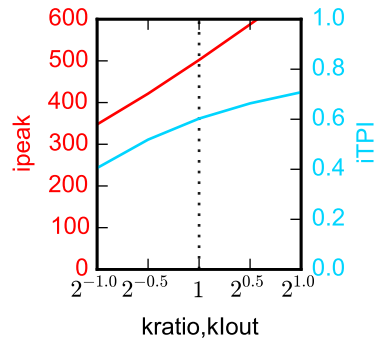

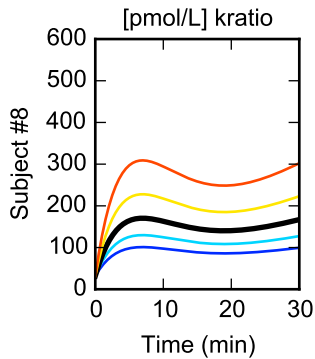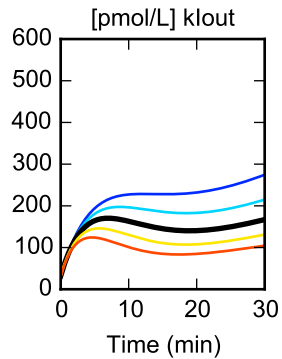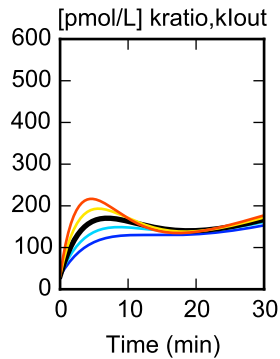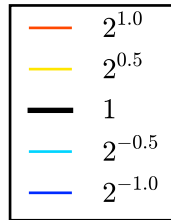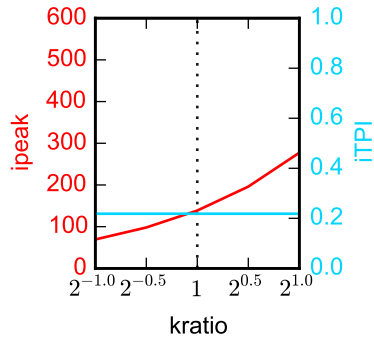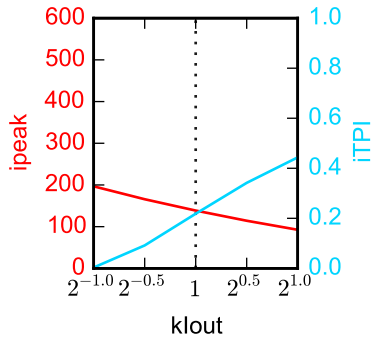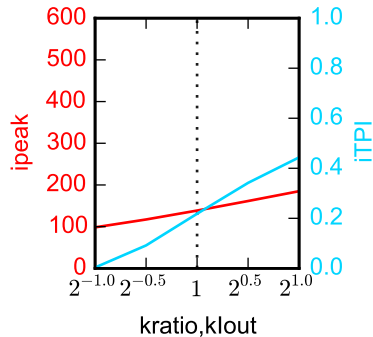

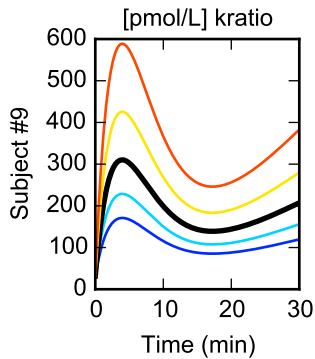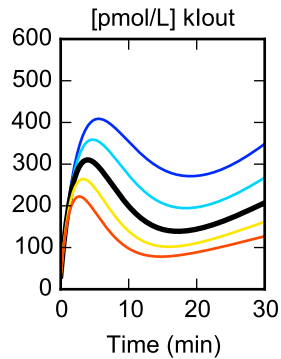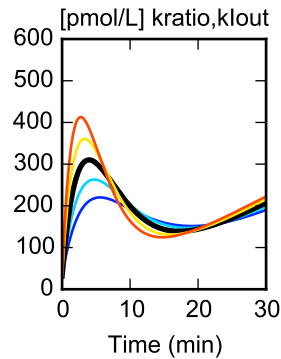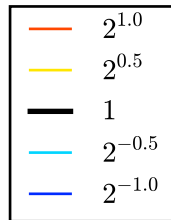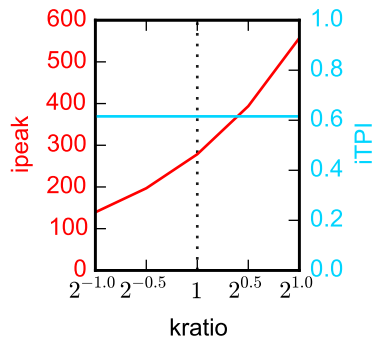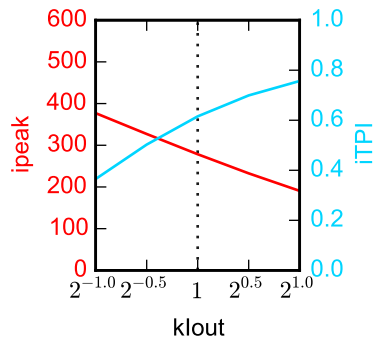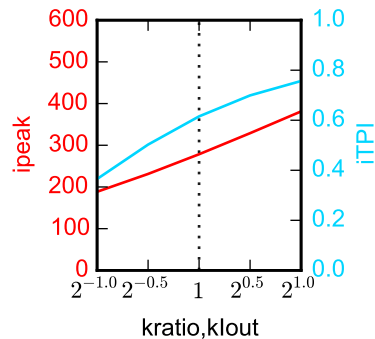

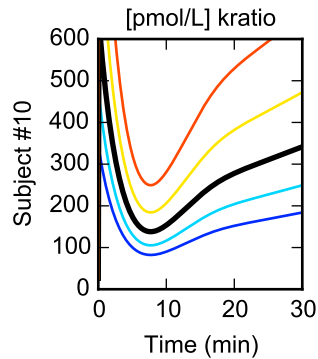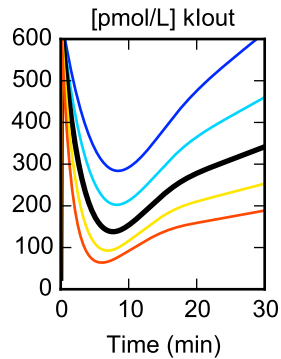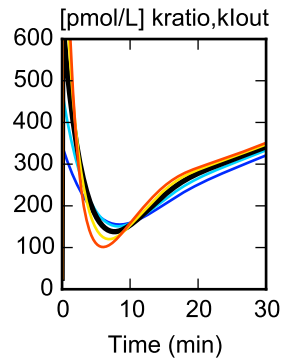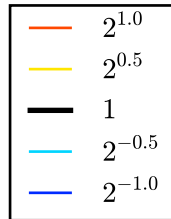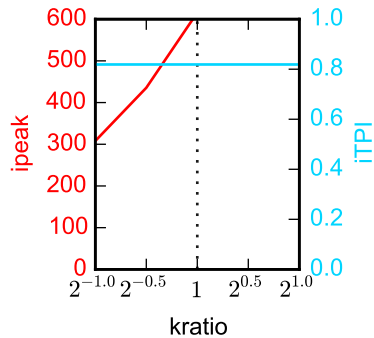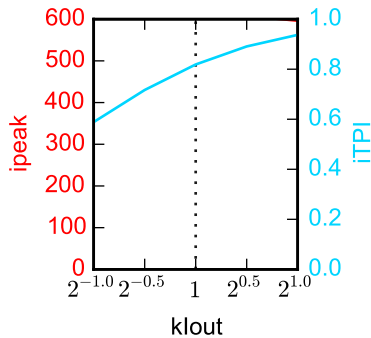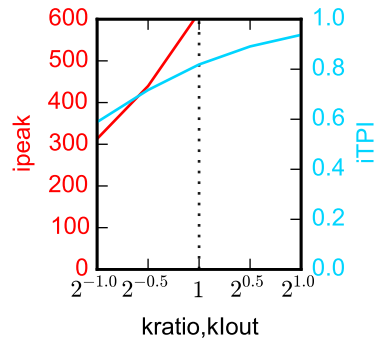

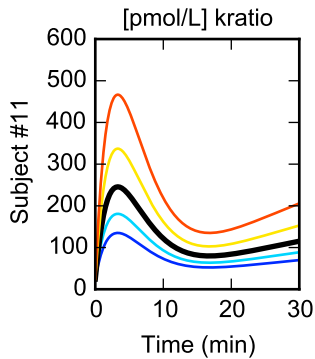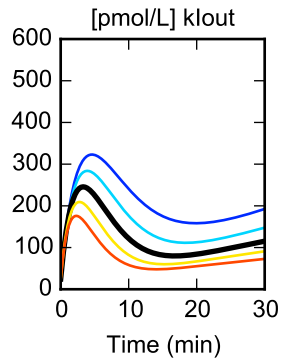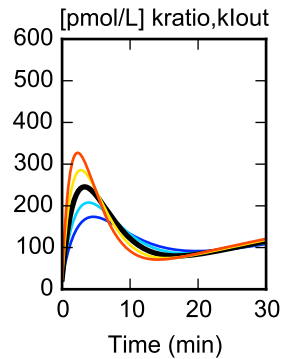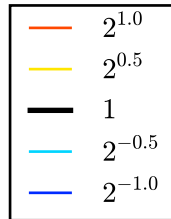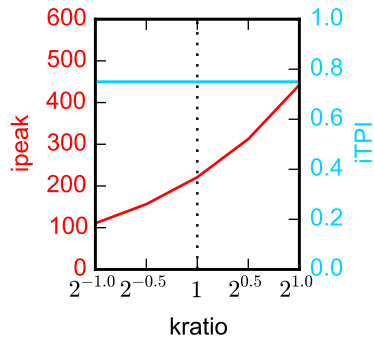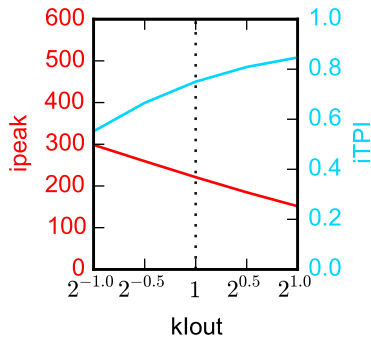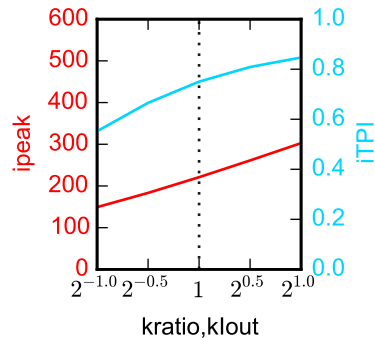

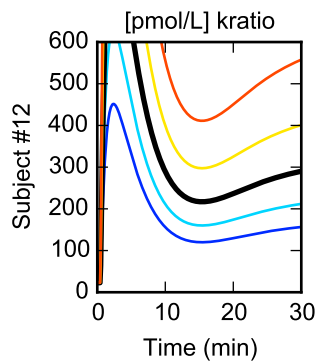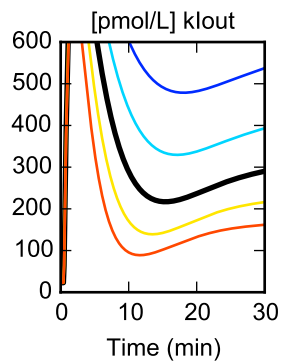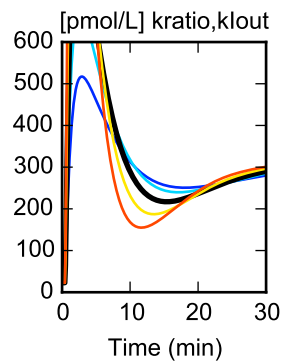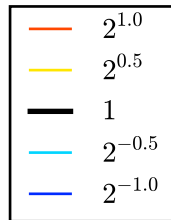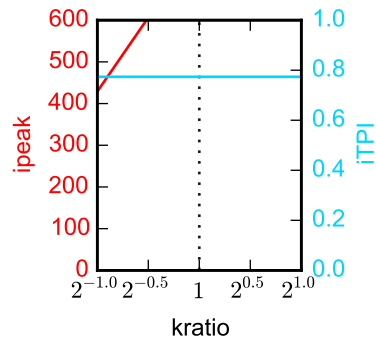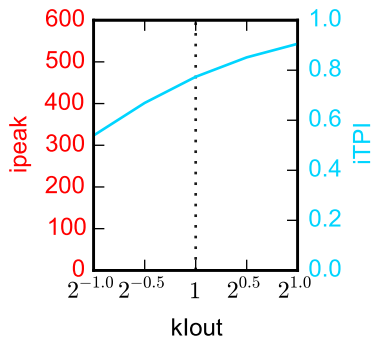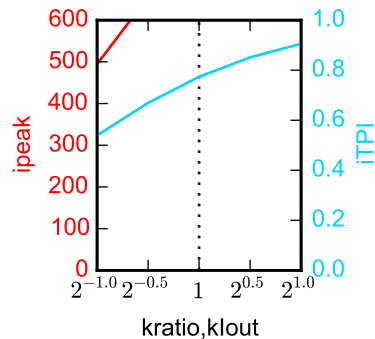

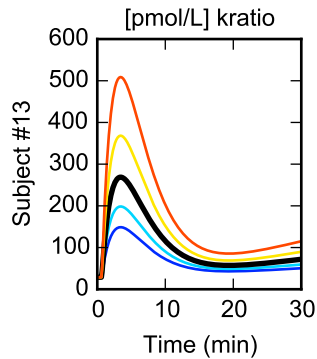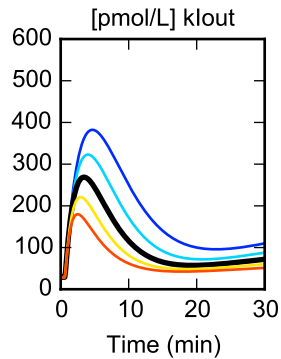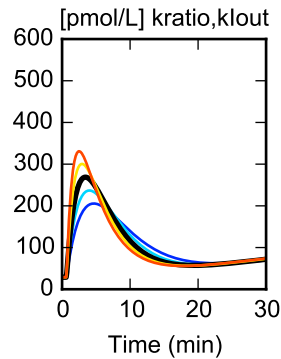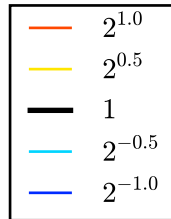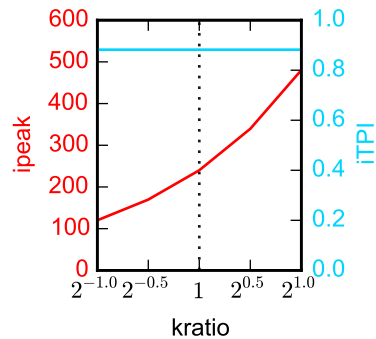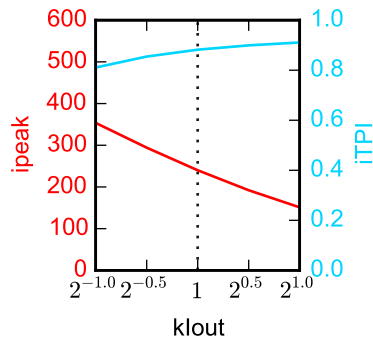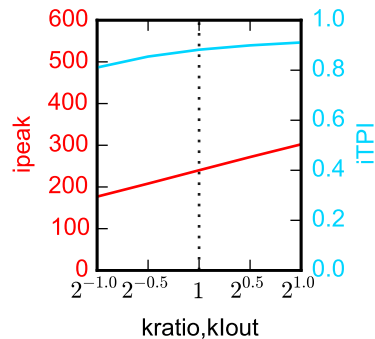

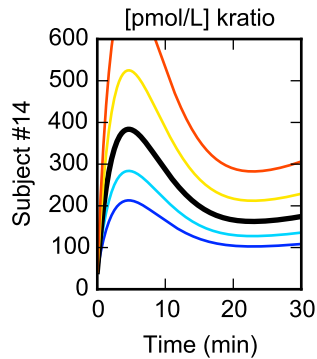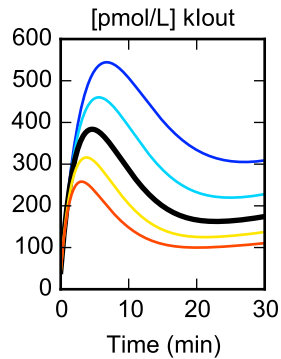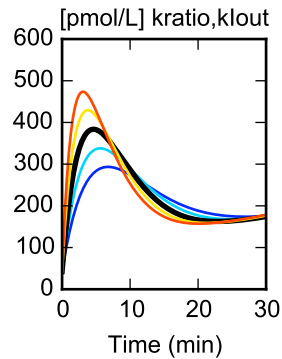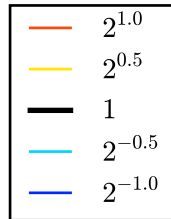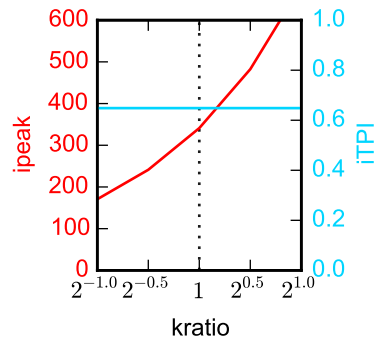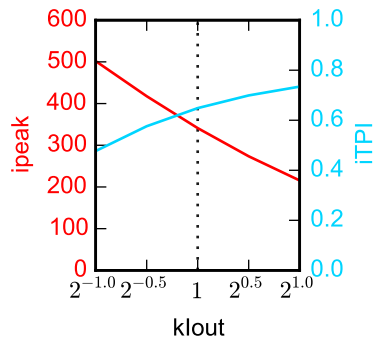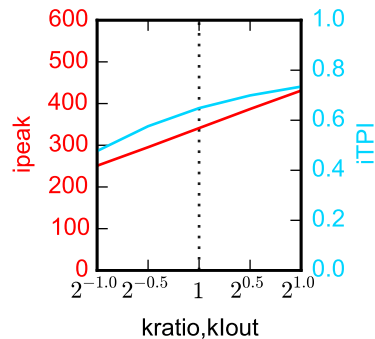

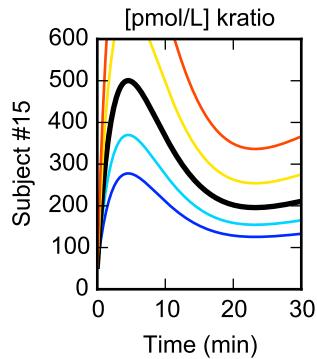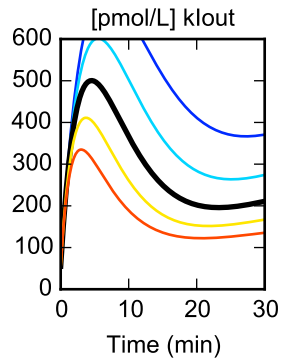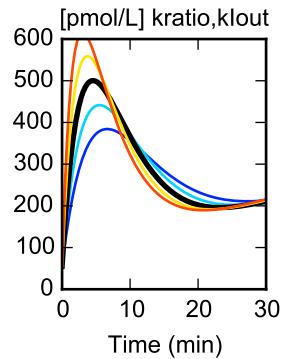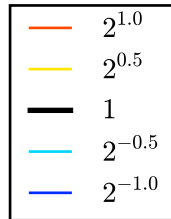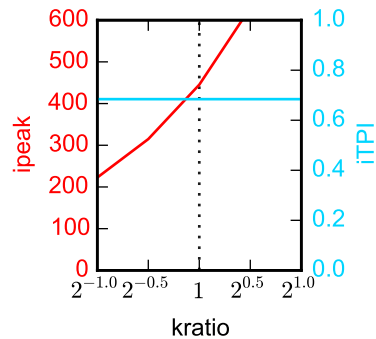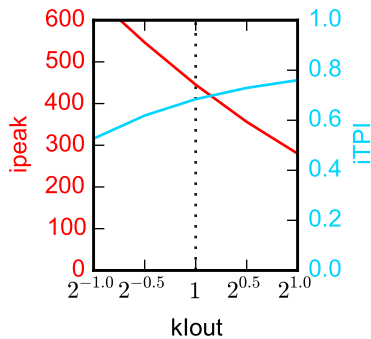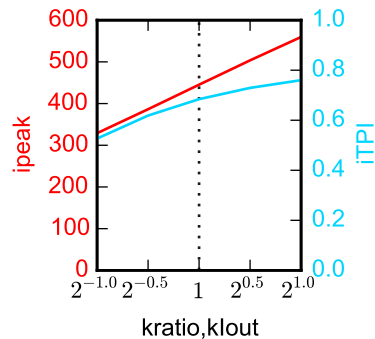

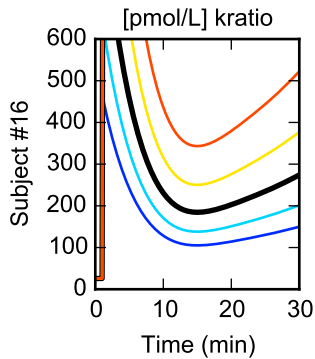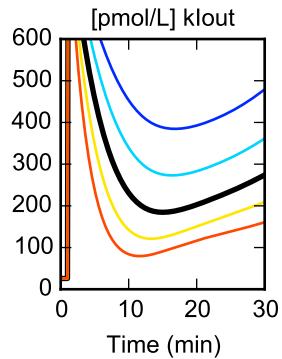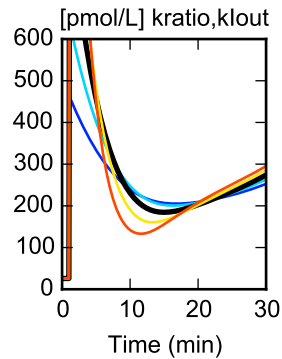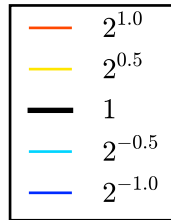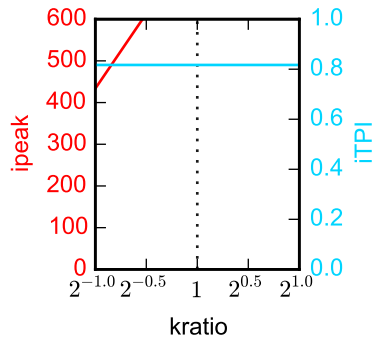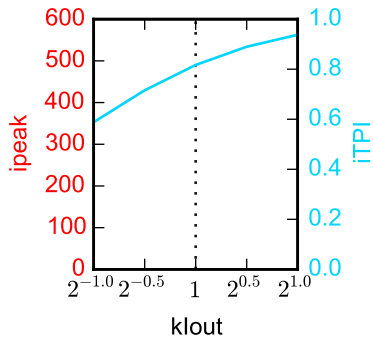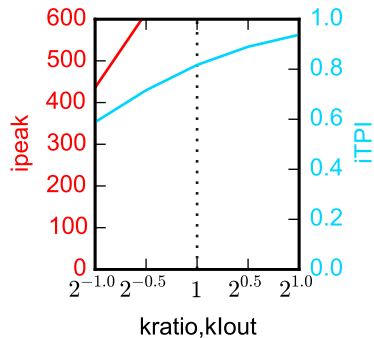

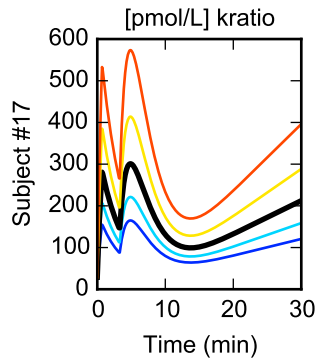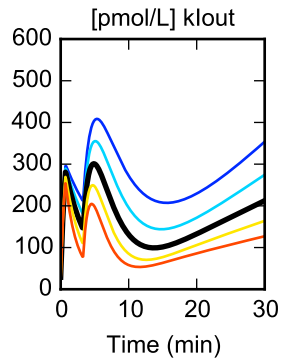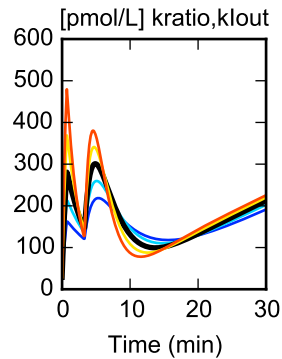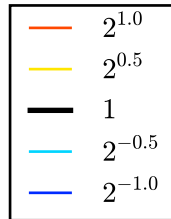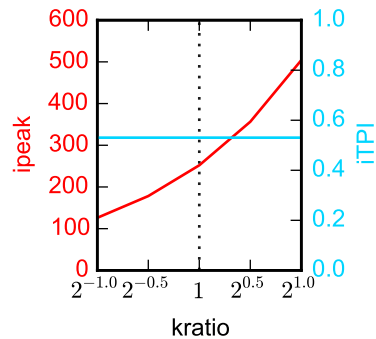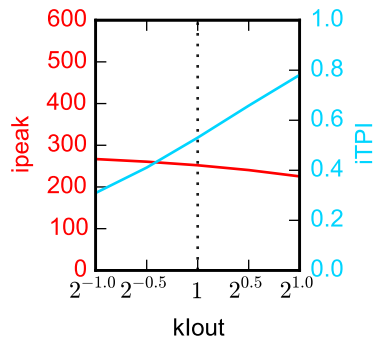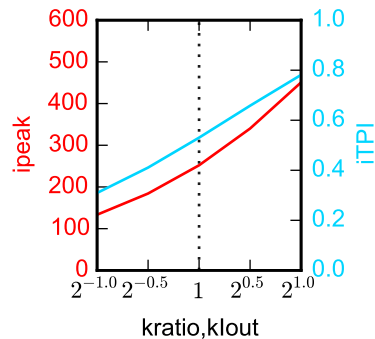

Subject #18

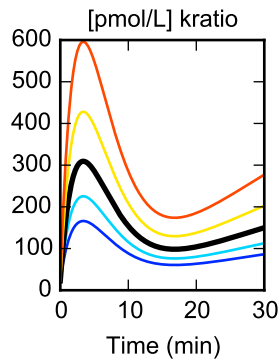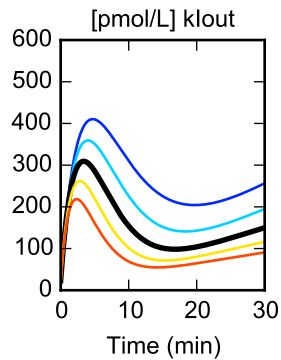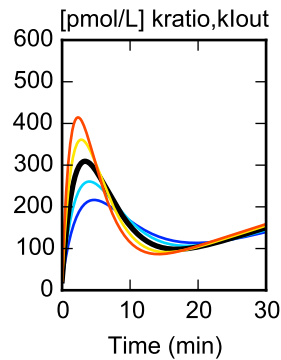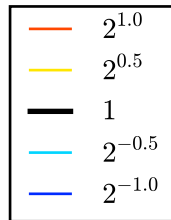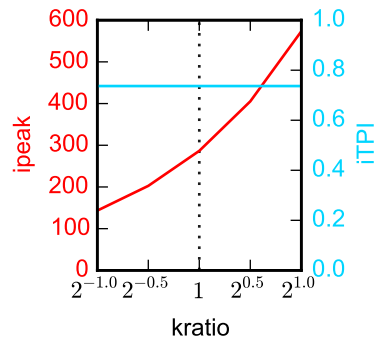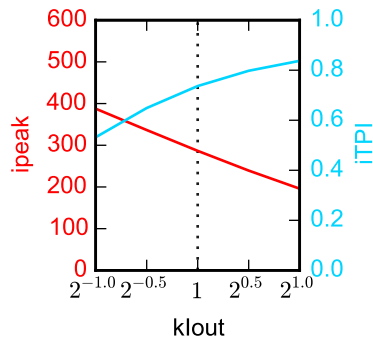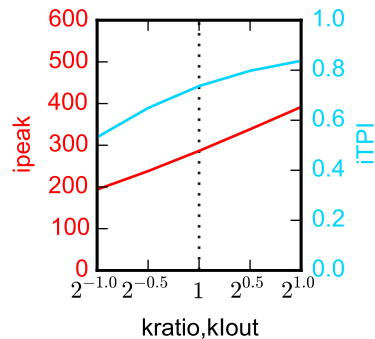

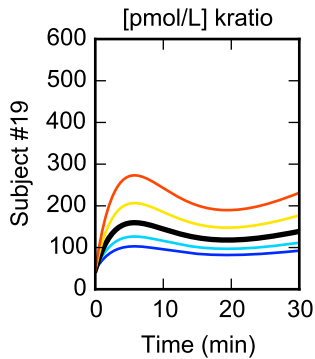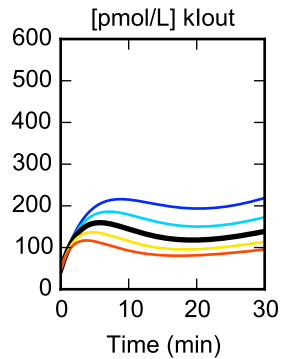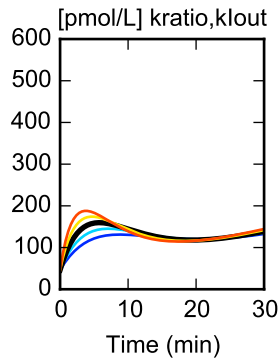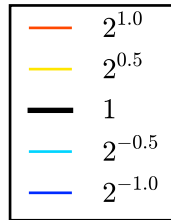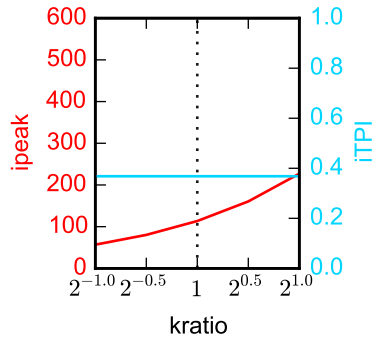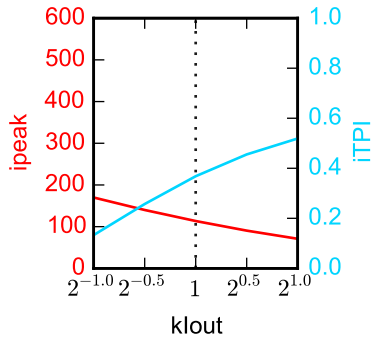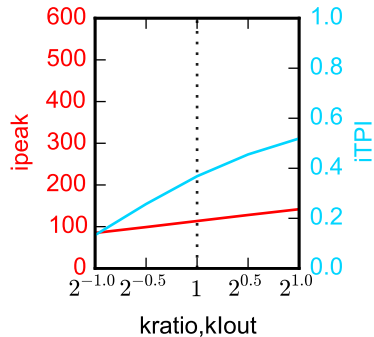

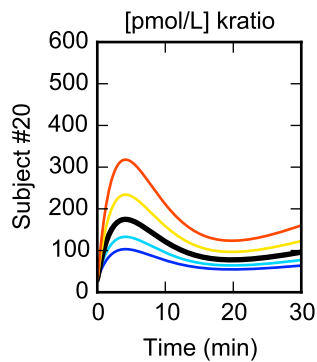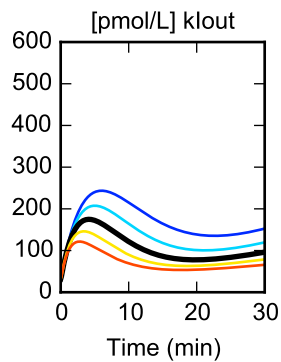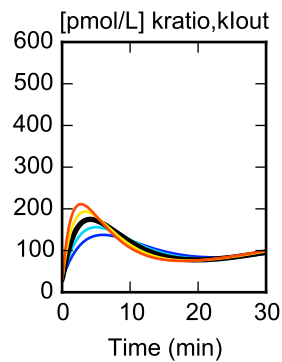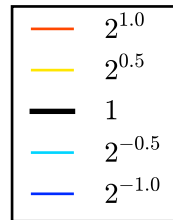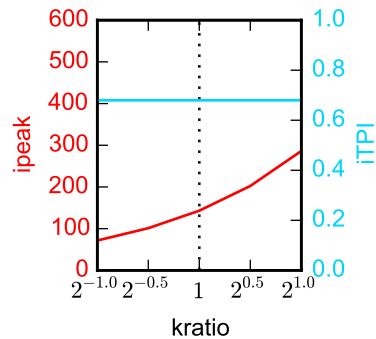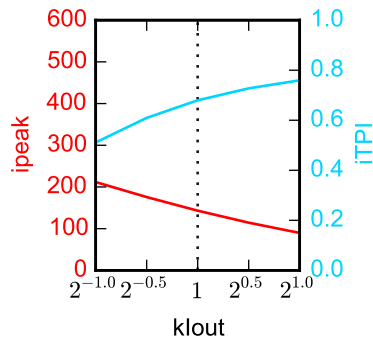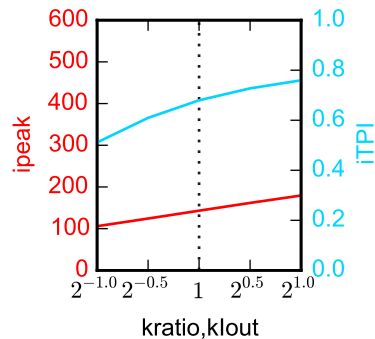

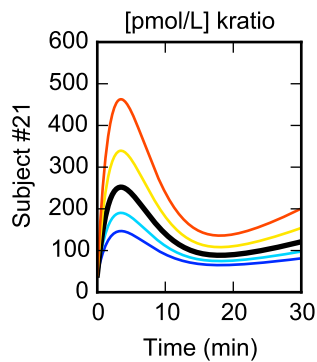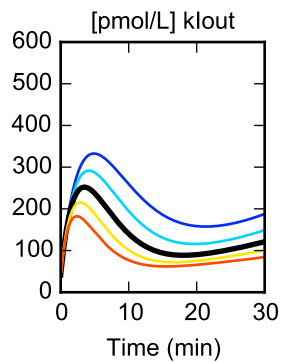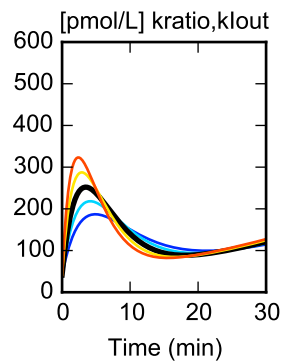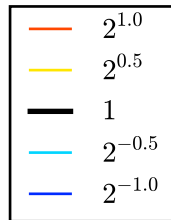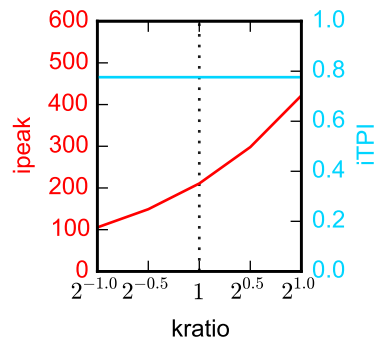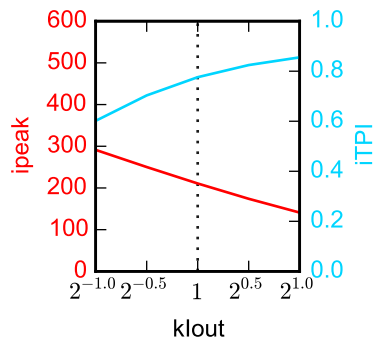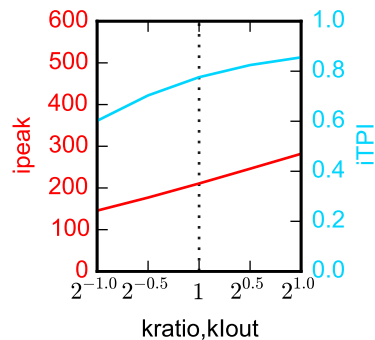

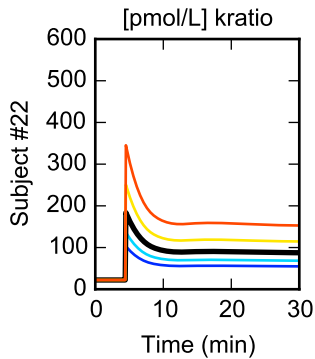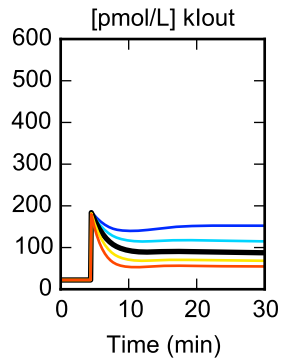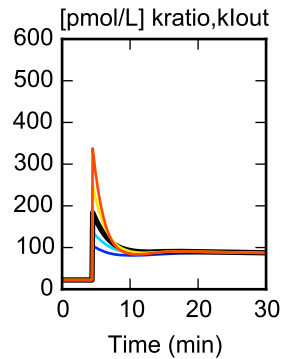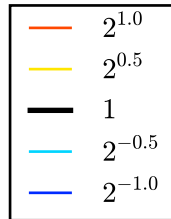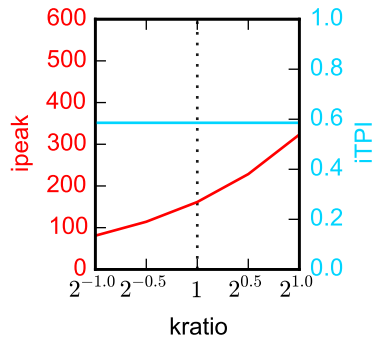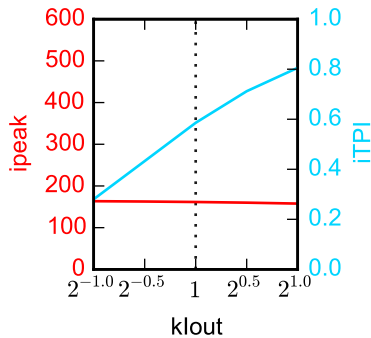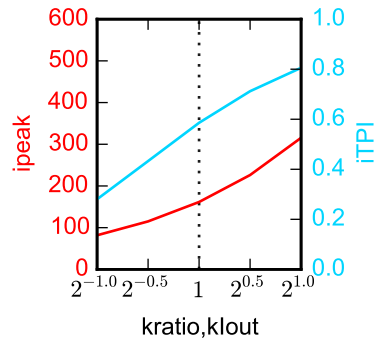

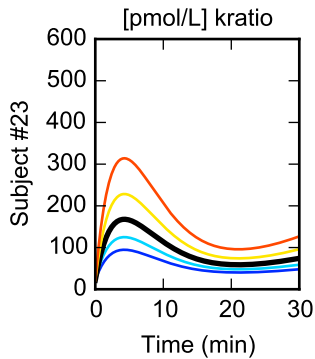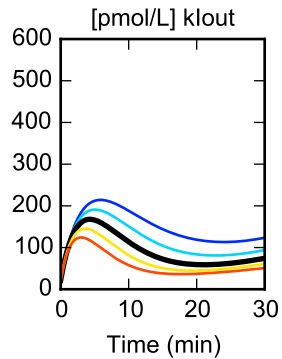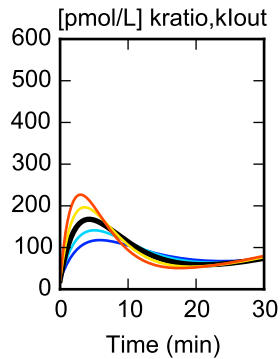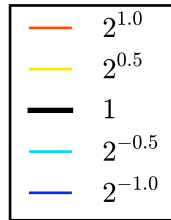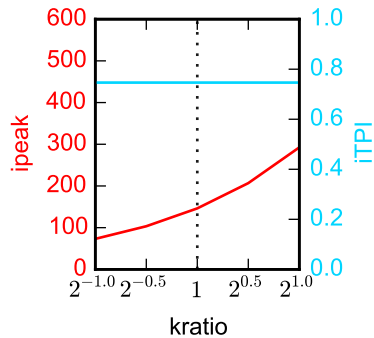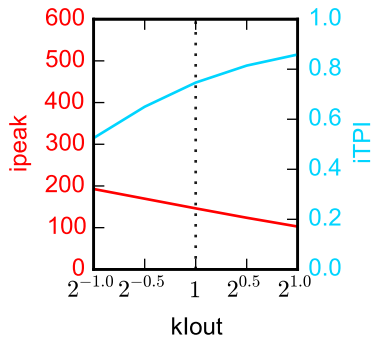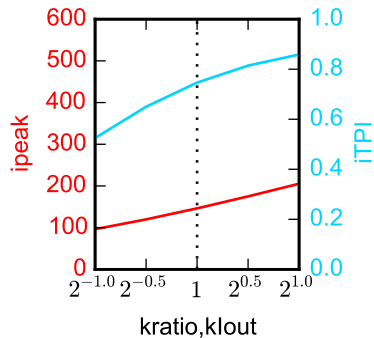

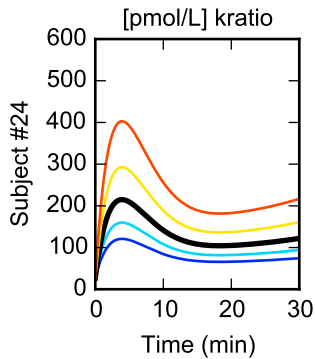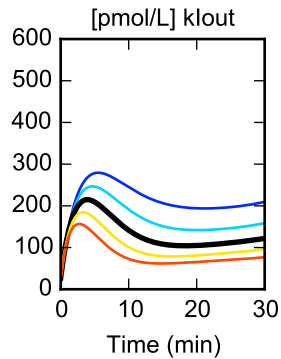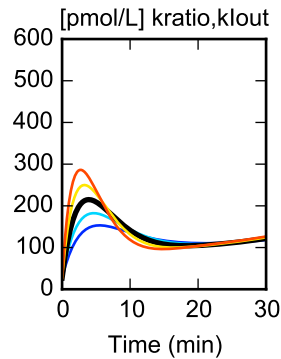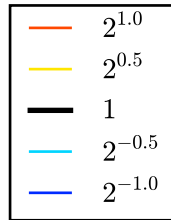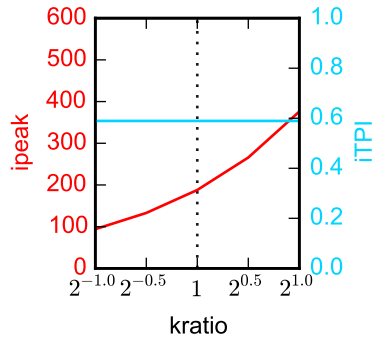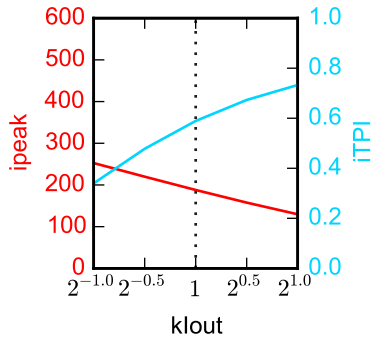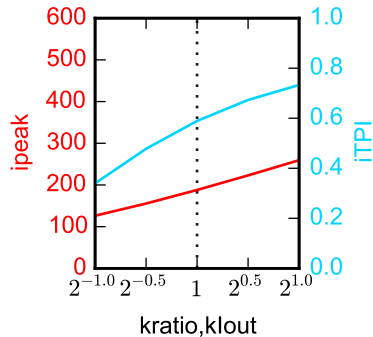

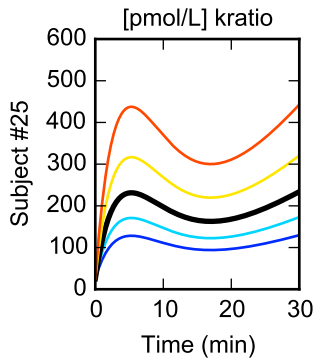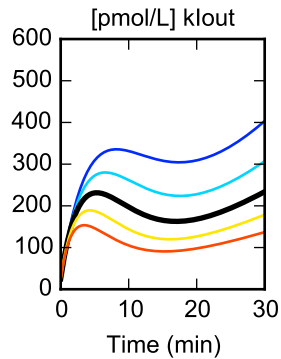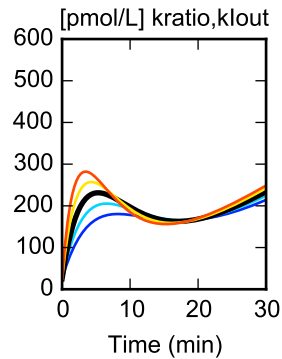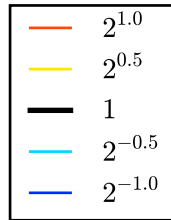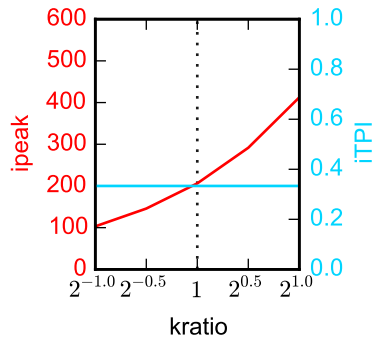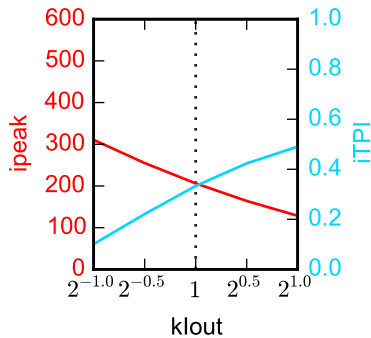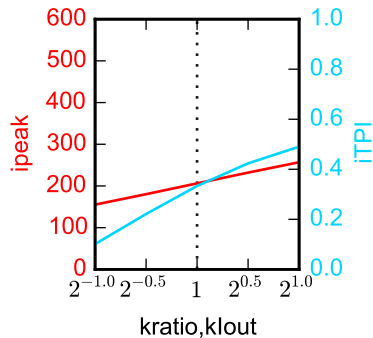

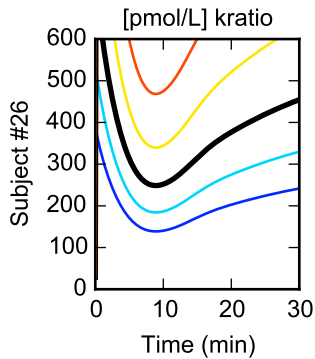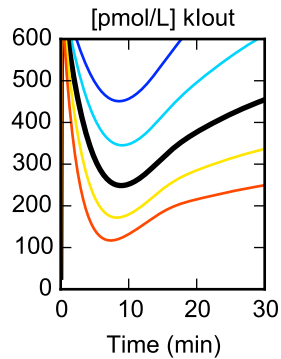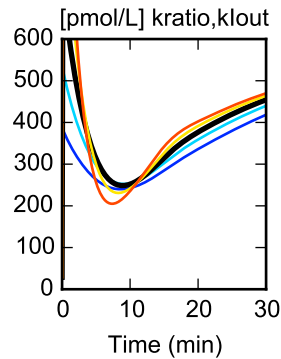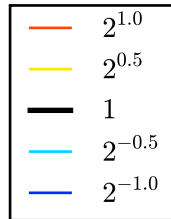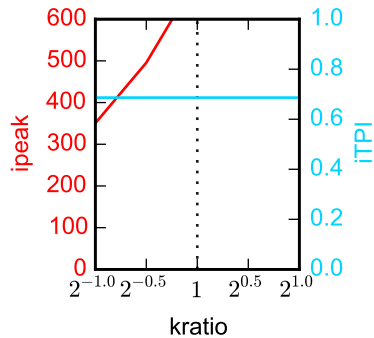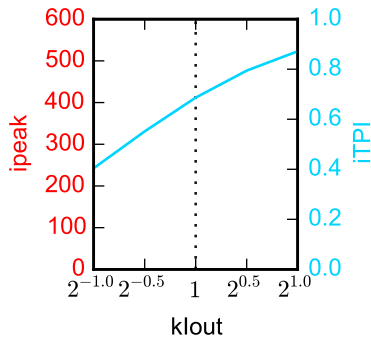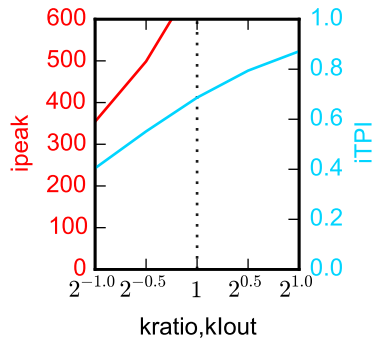

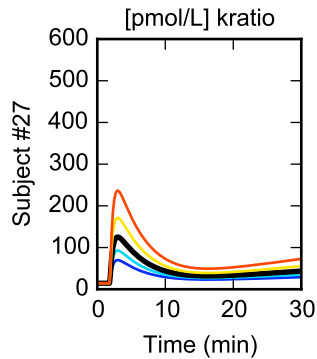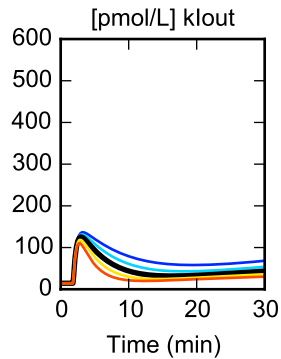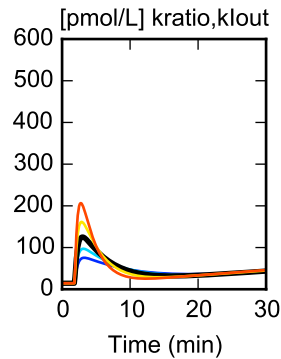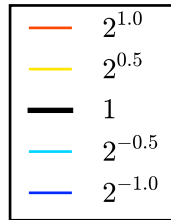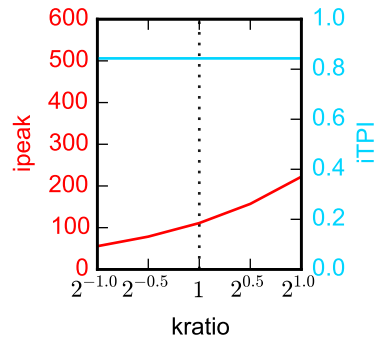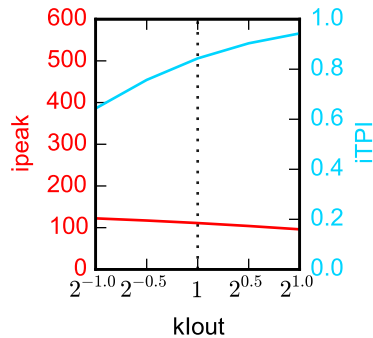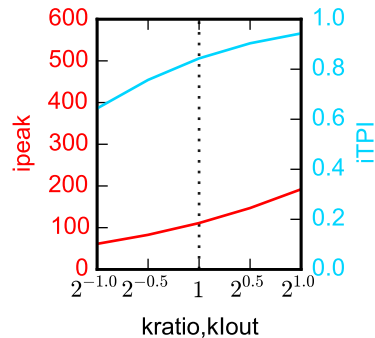

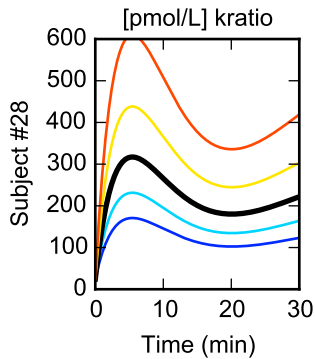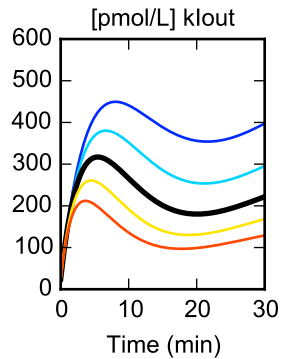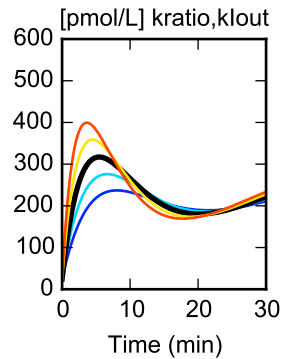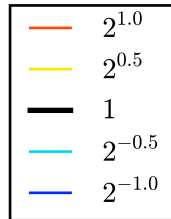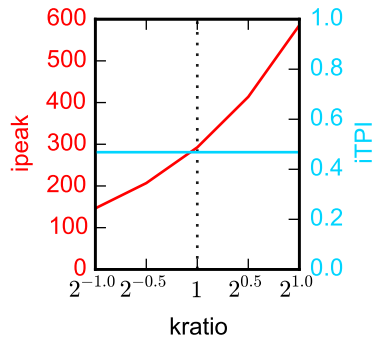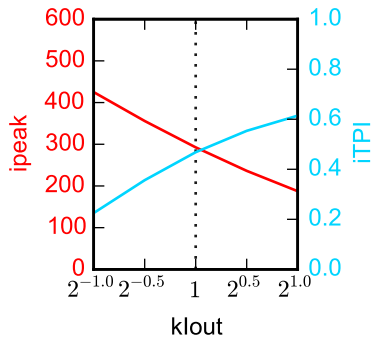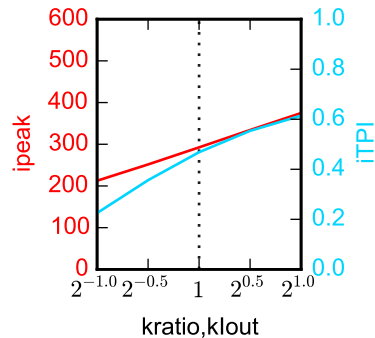

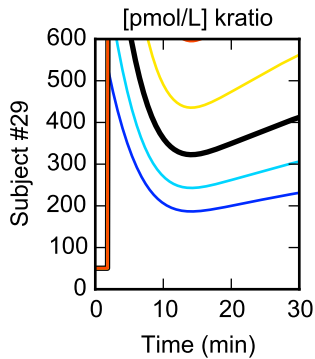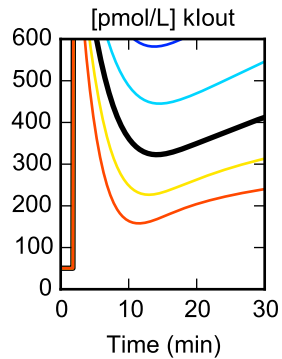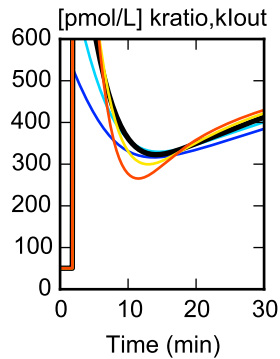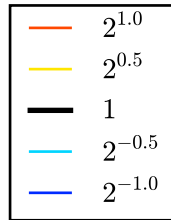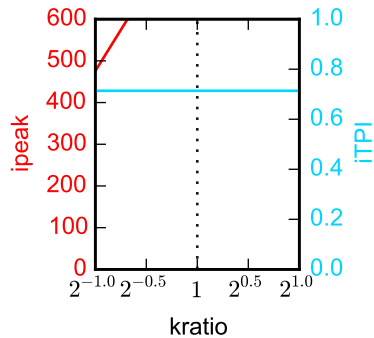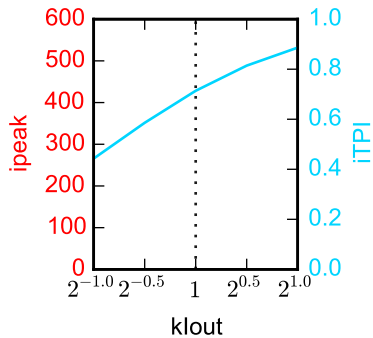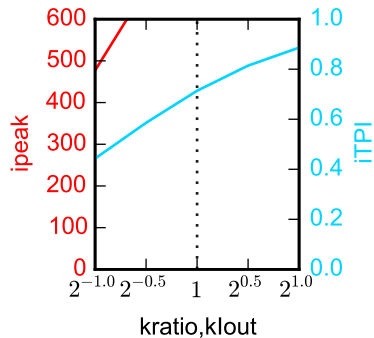

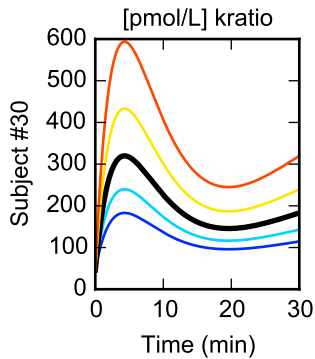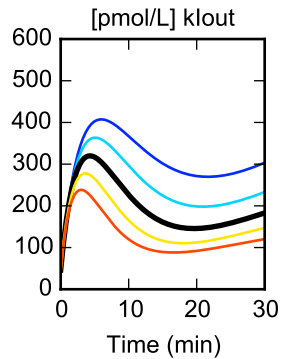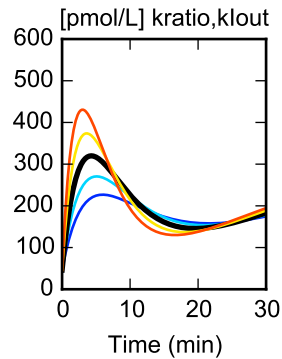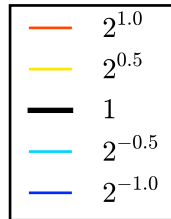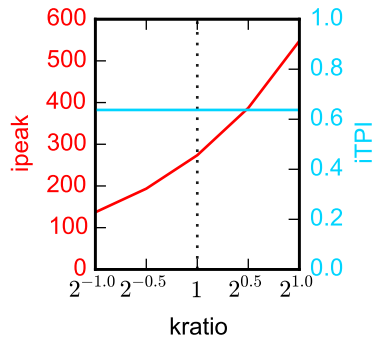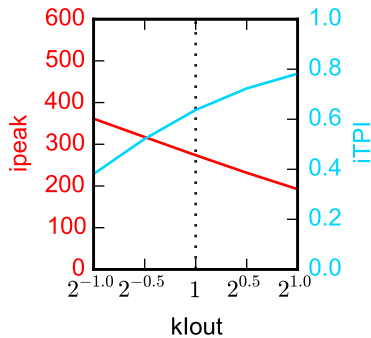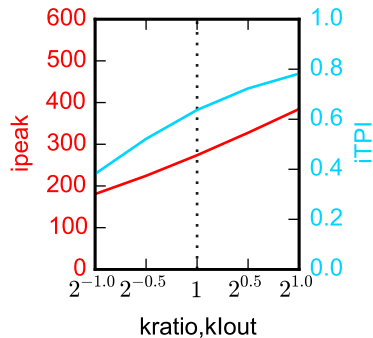

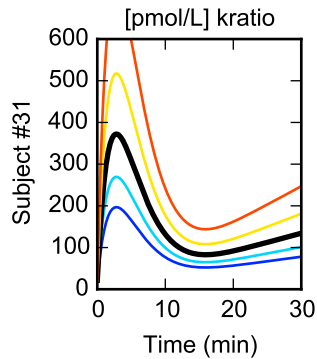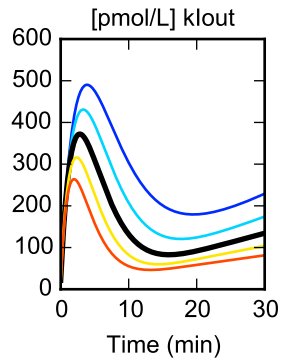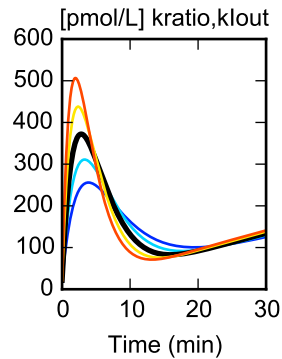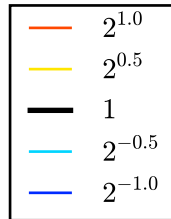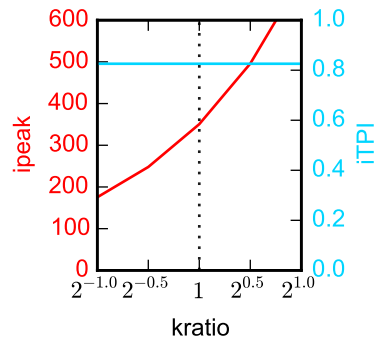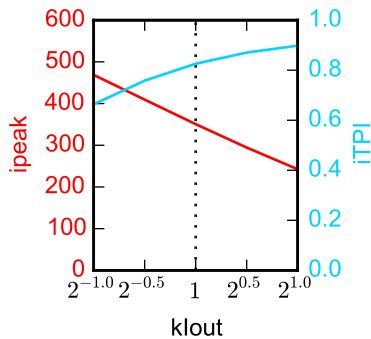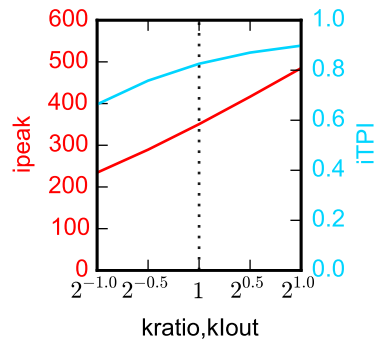

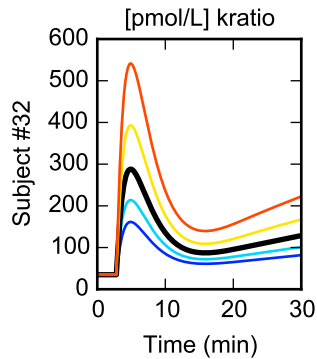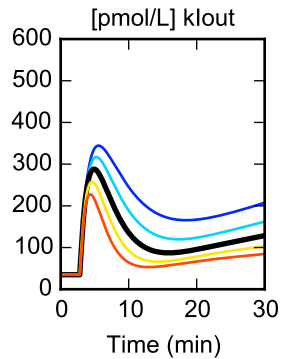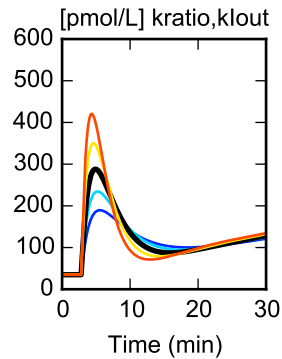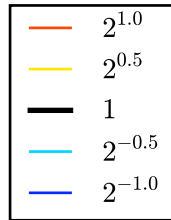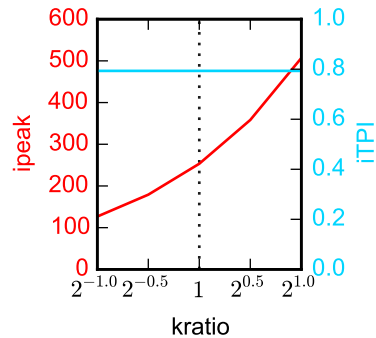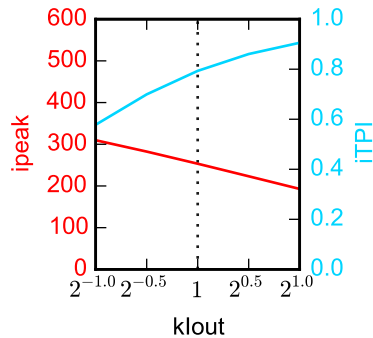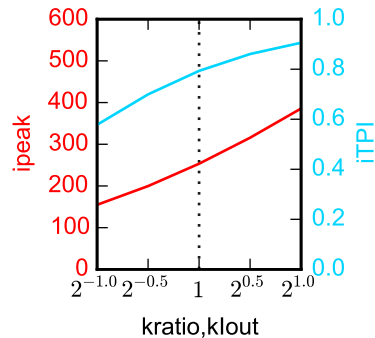

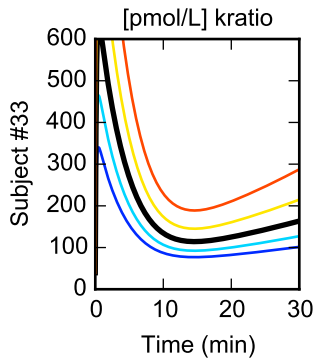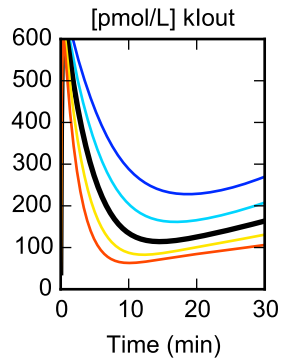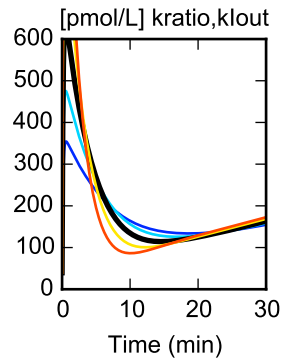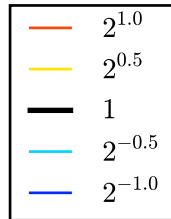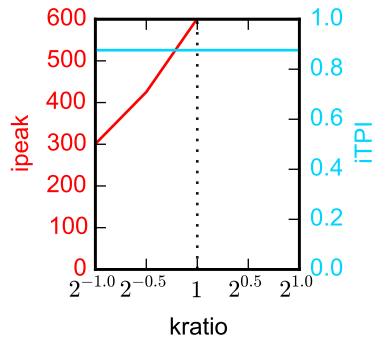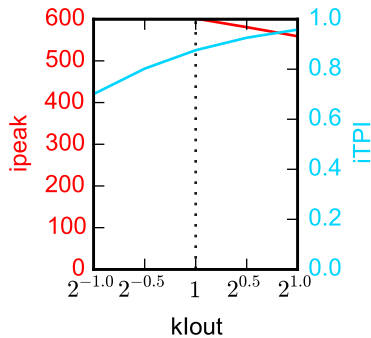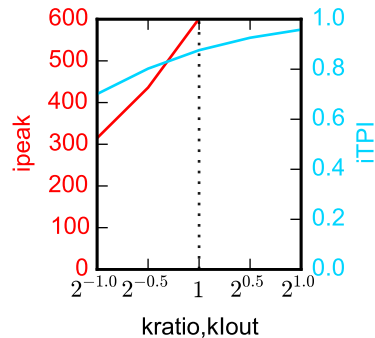

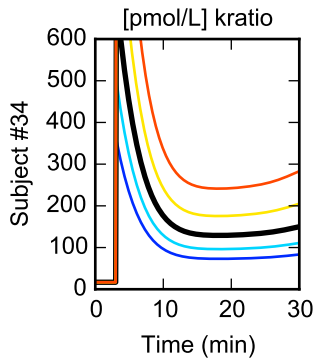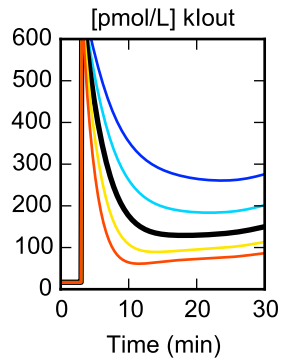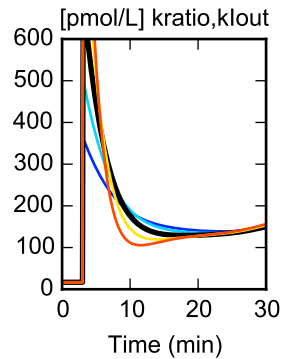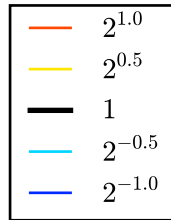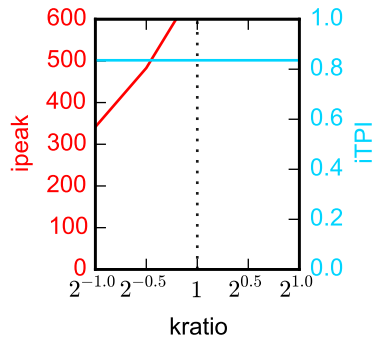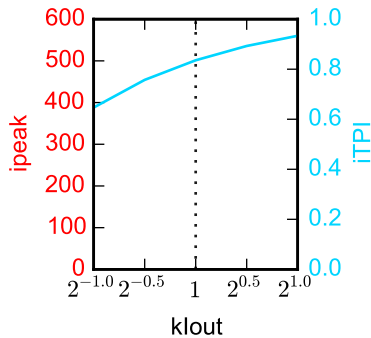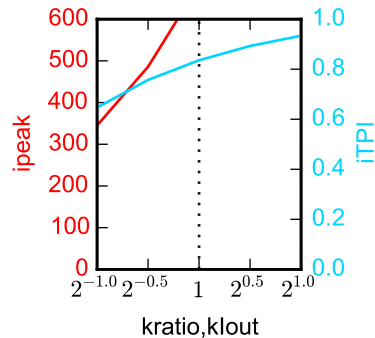

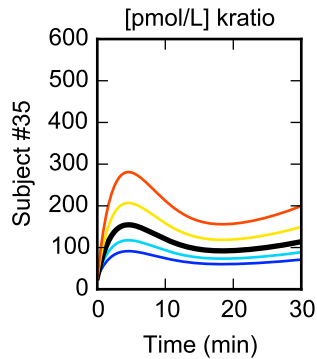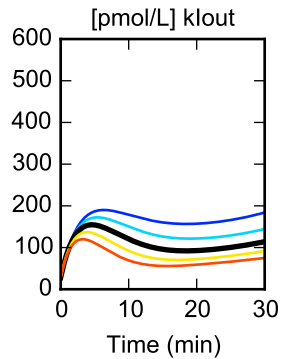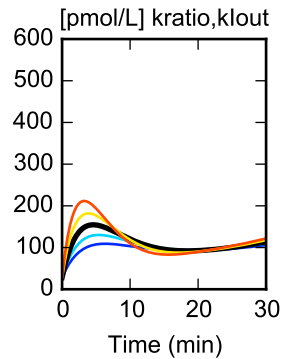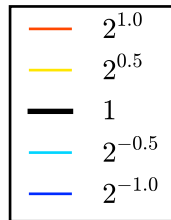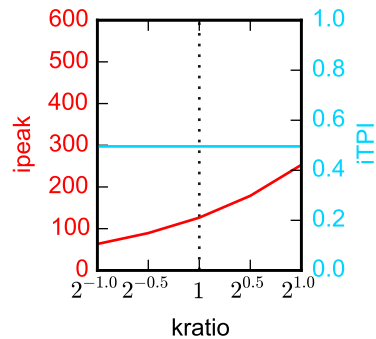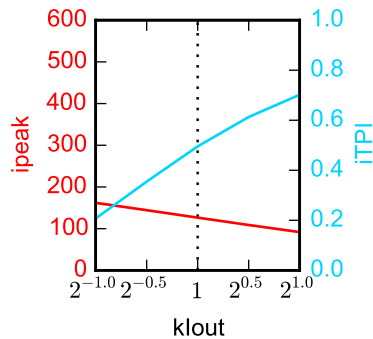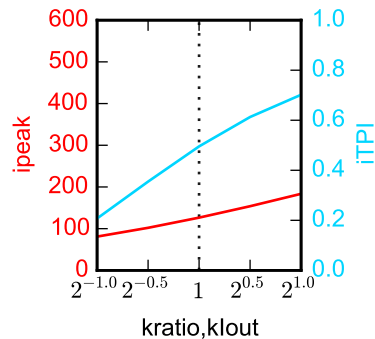

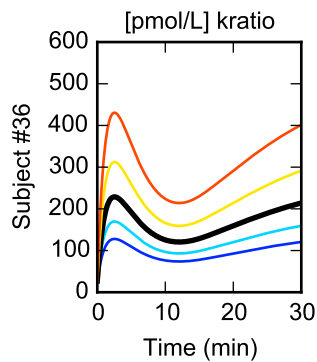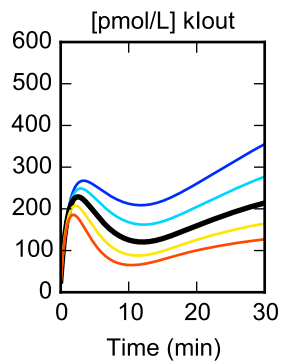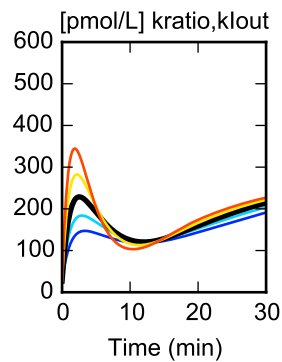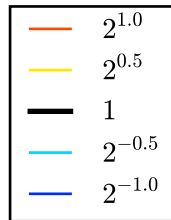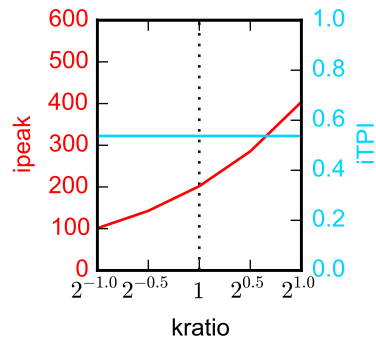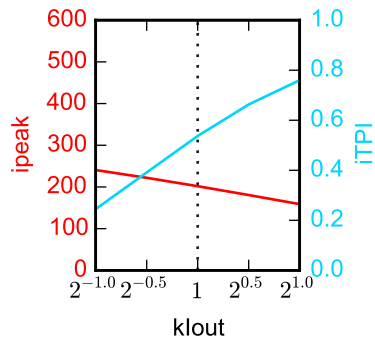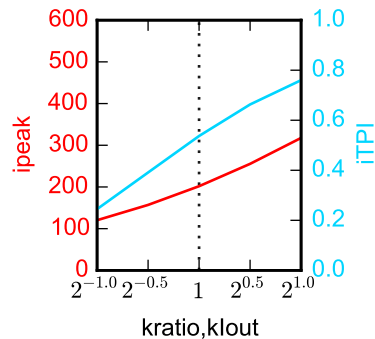

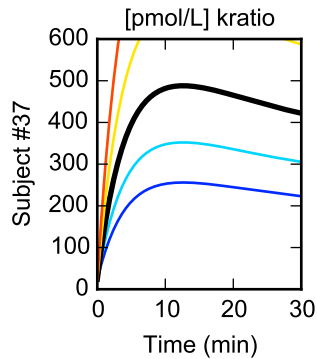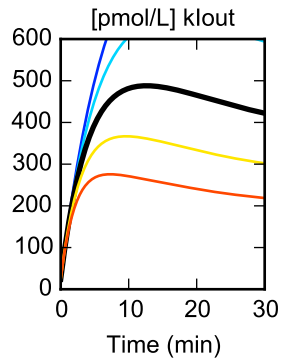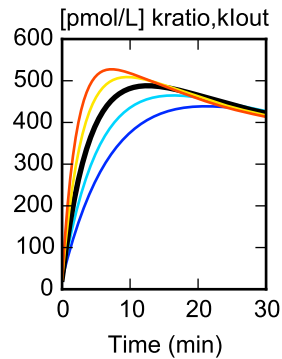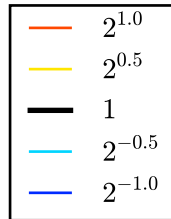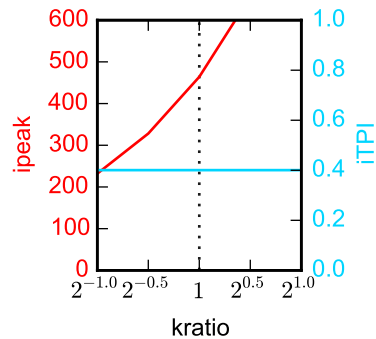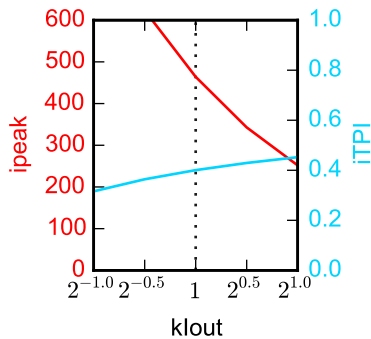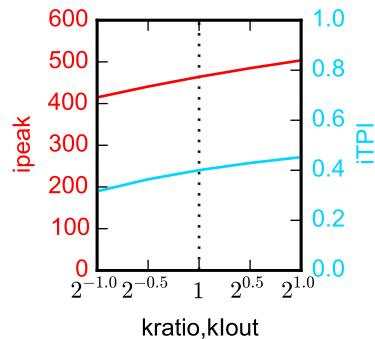

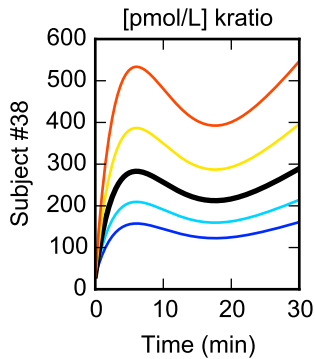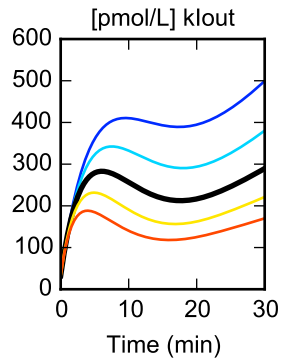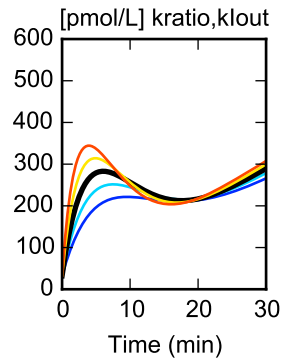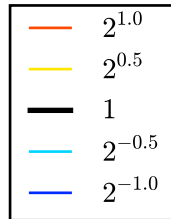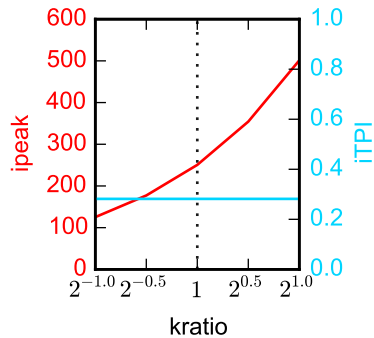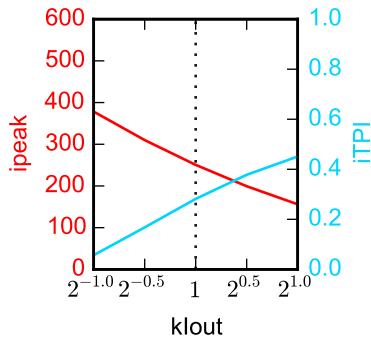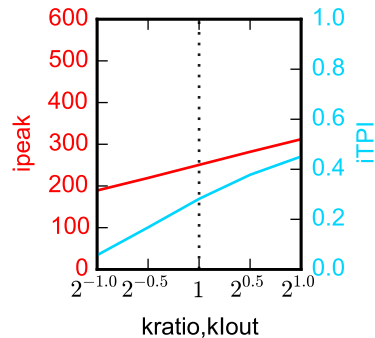

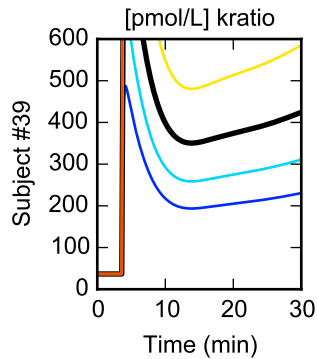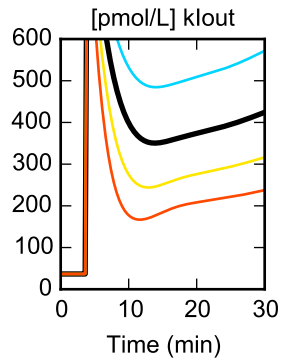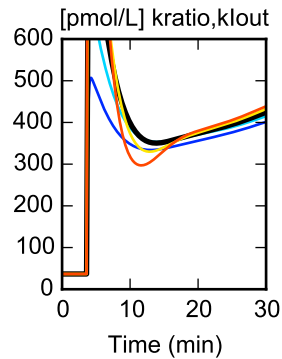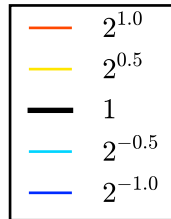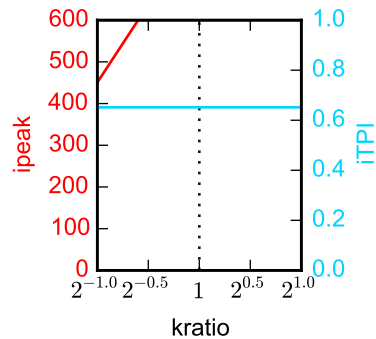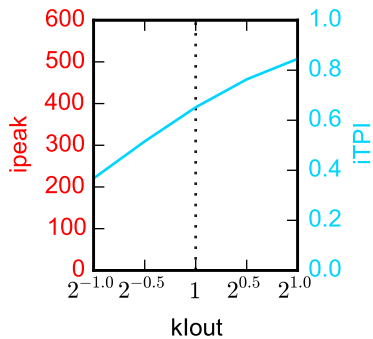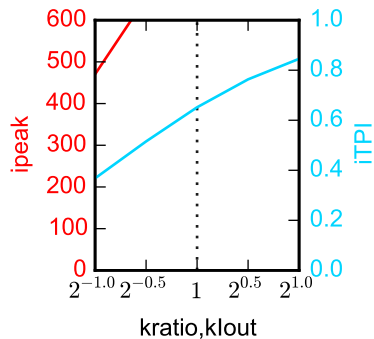

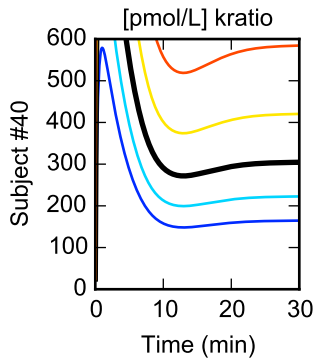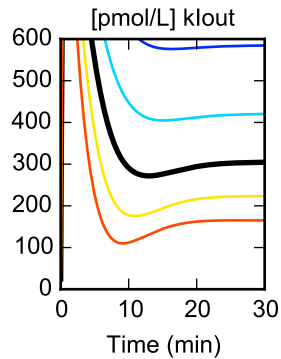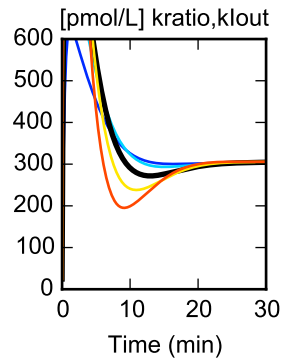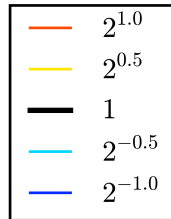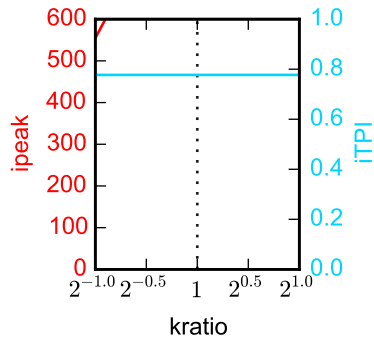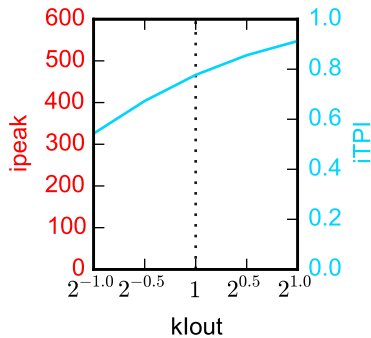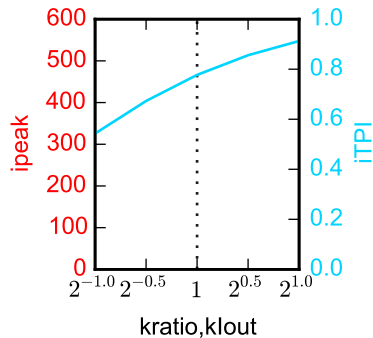

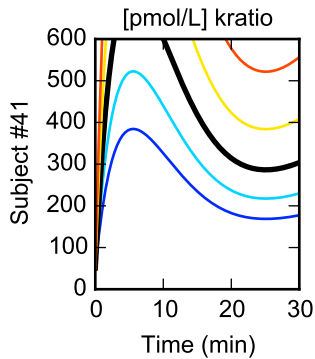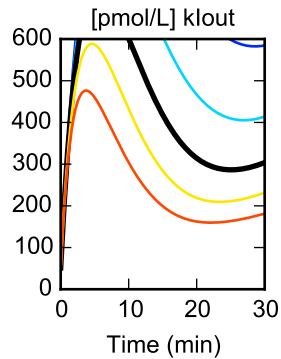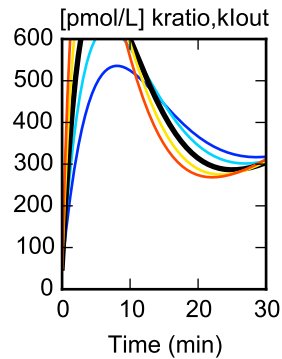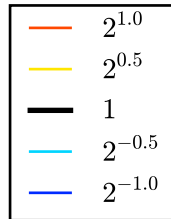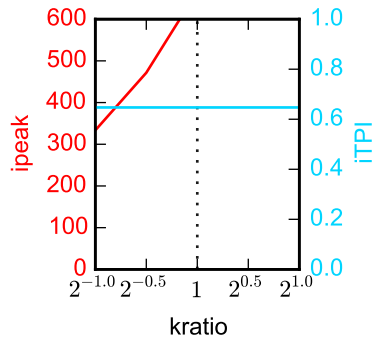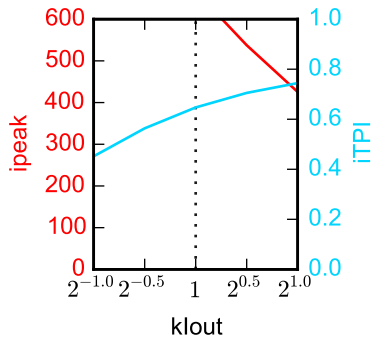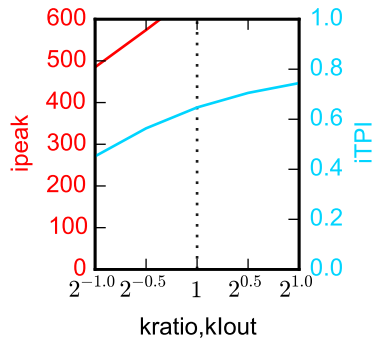

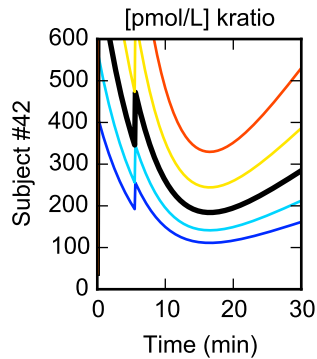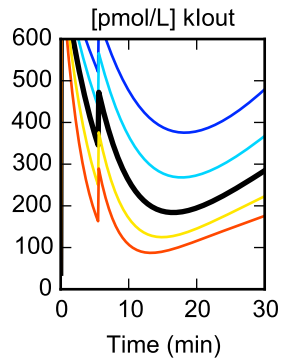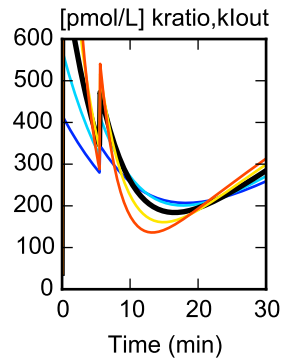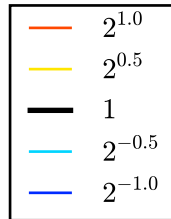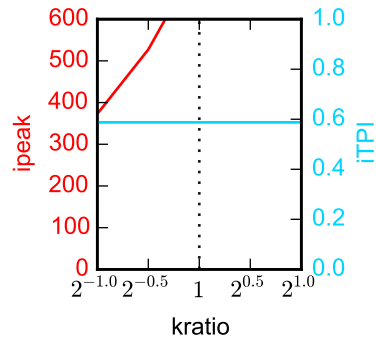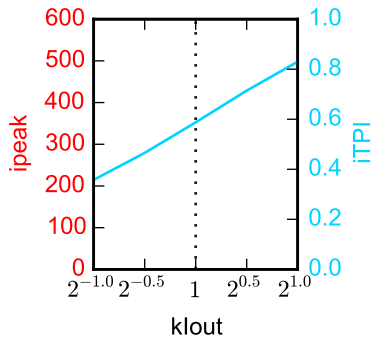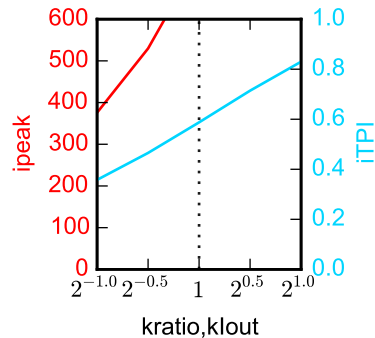

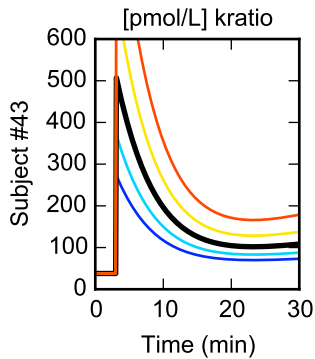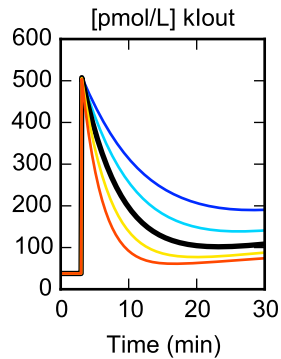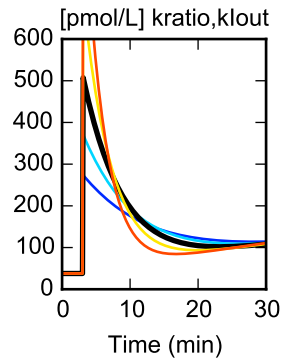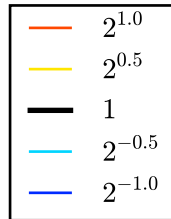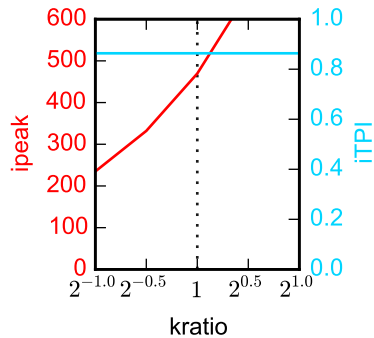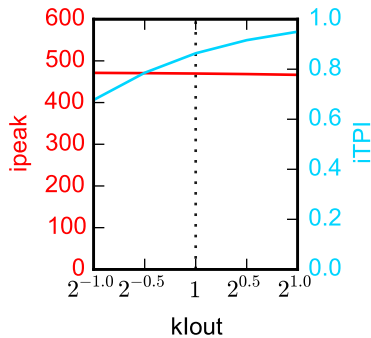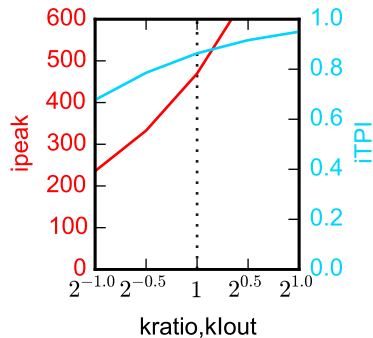

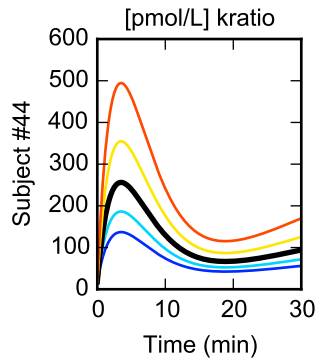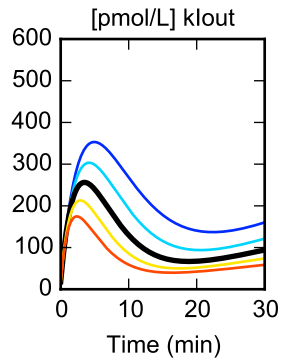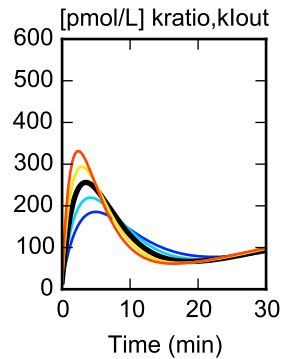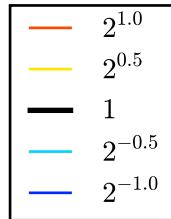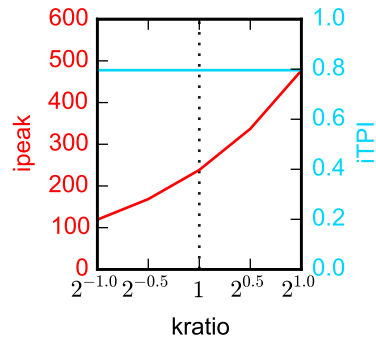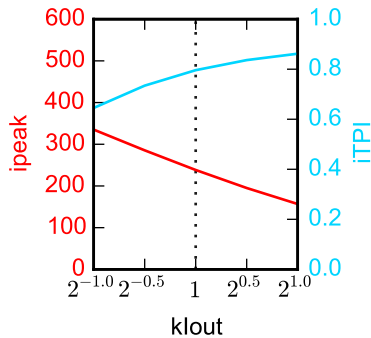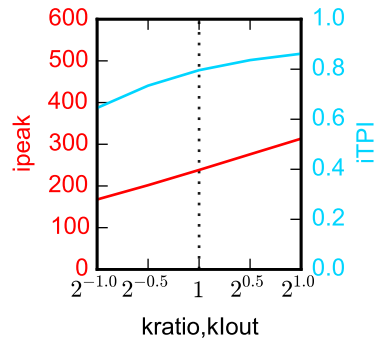

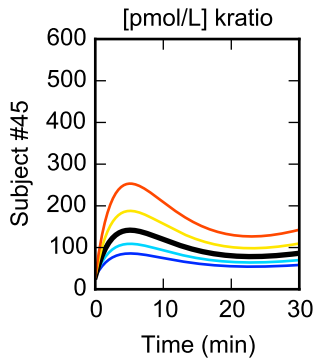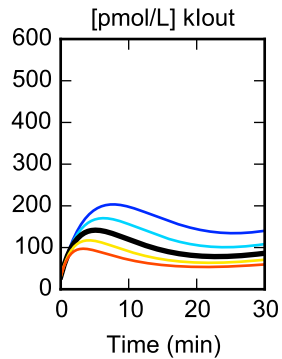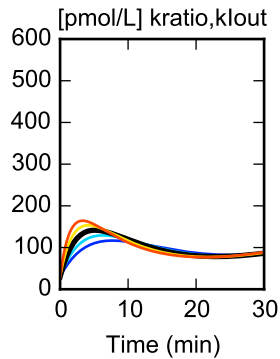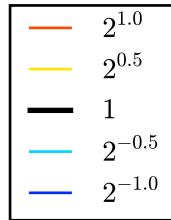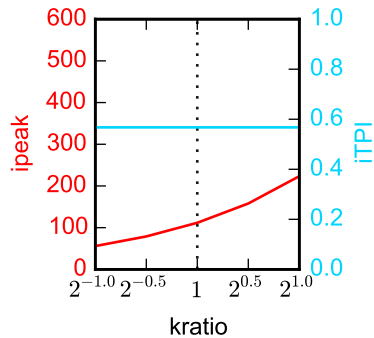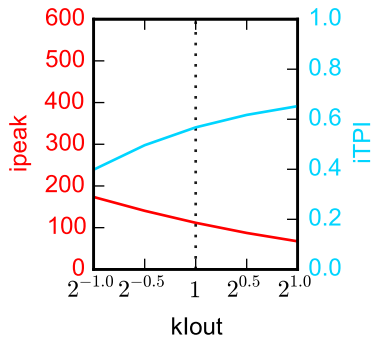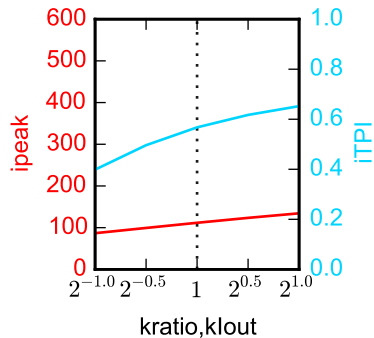

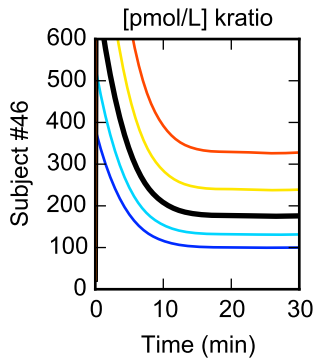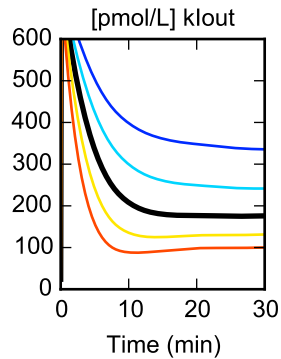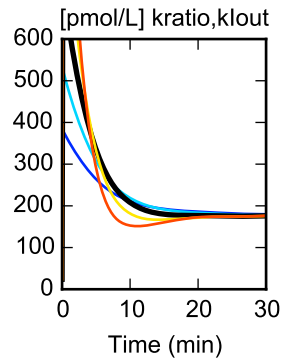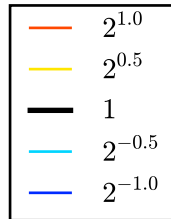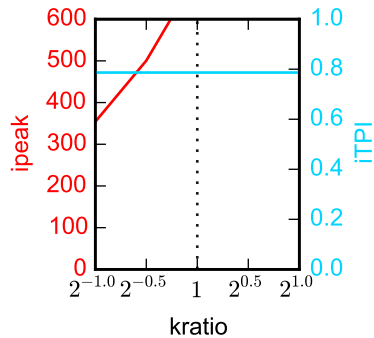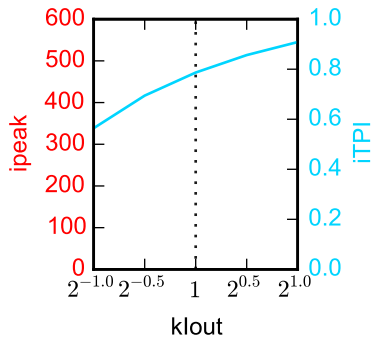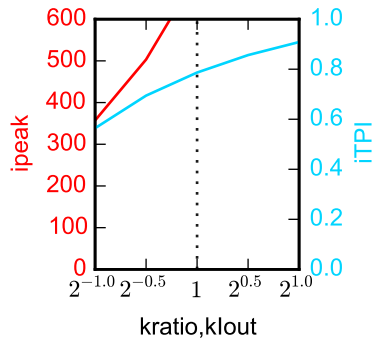

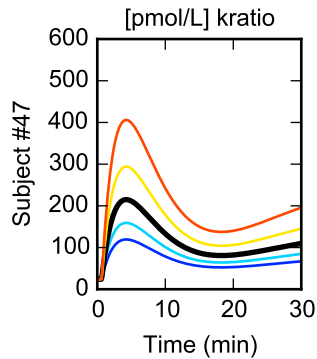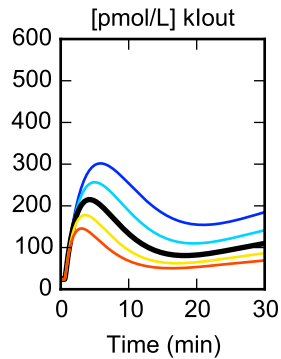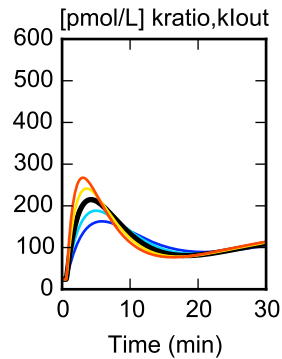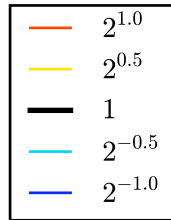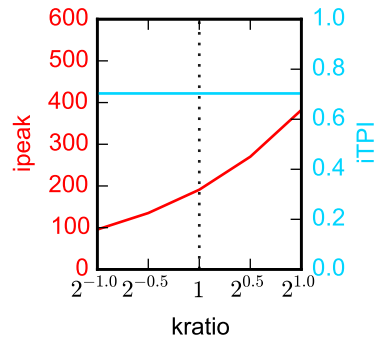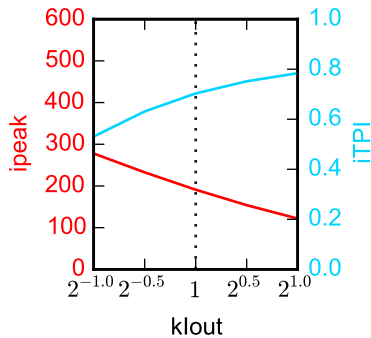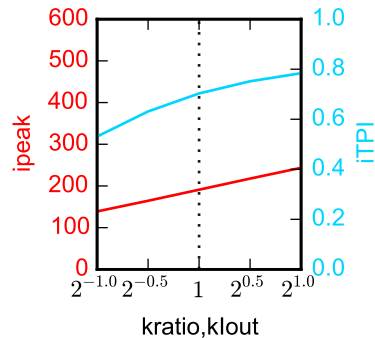

Subject #48

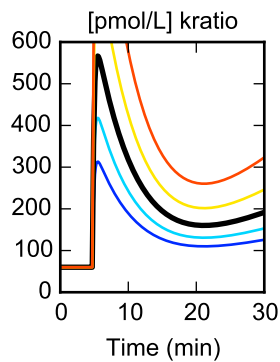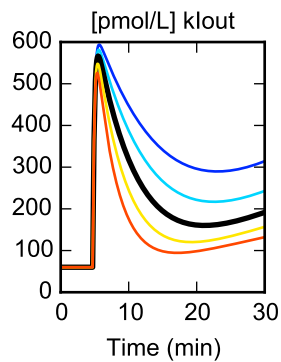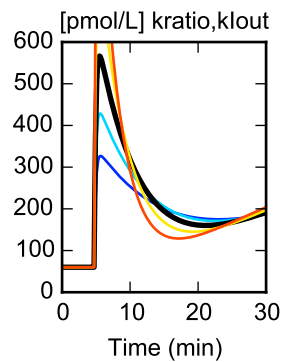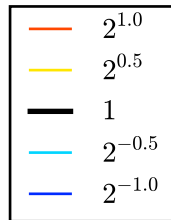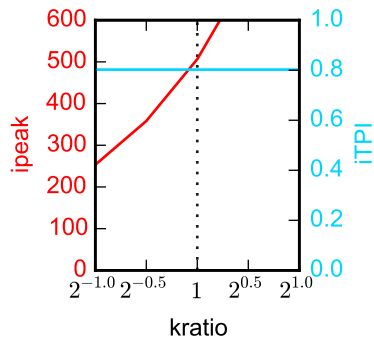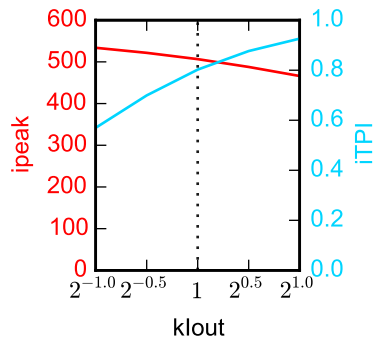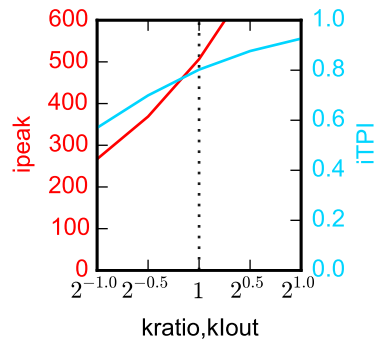

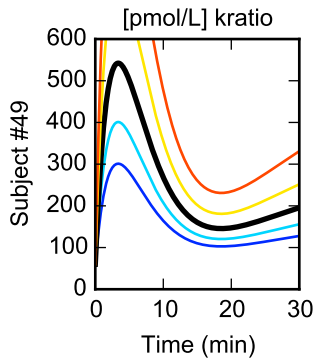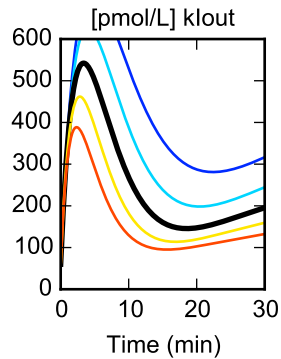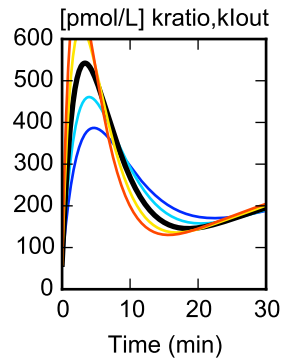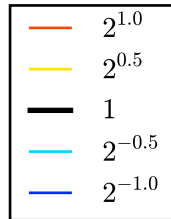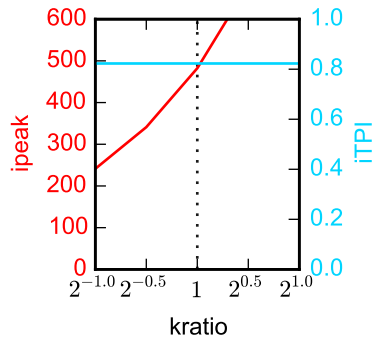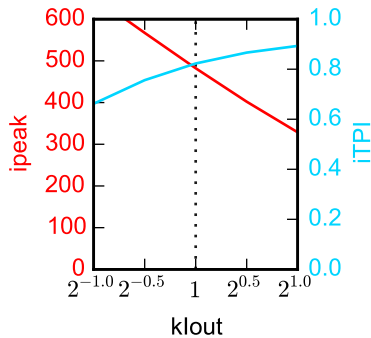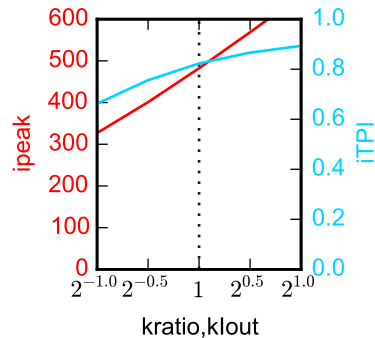

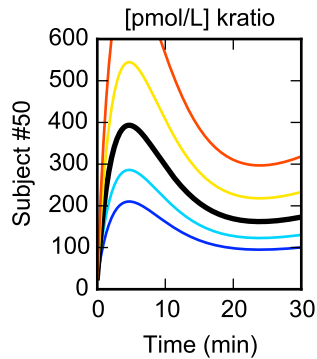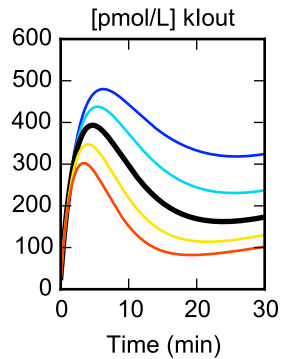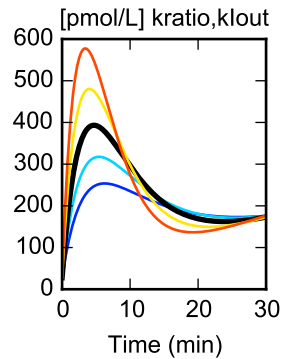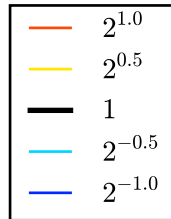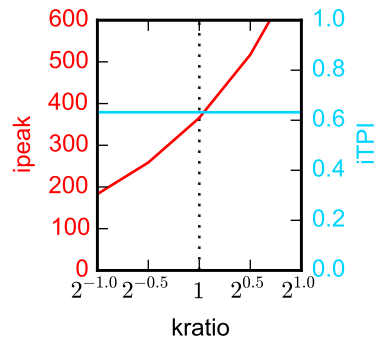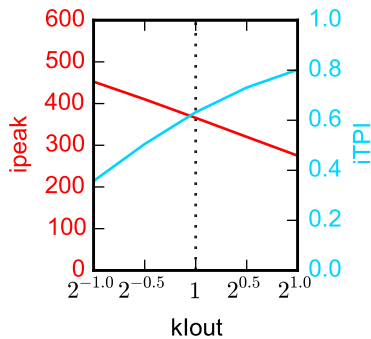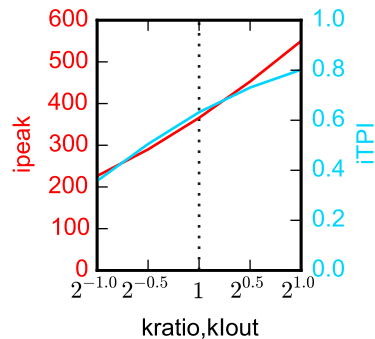

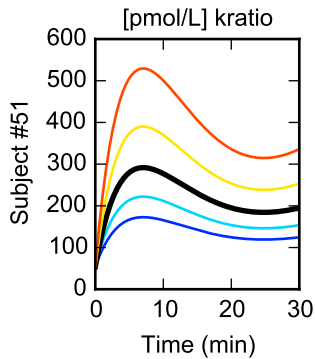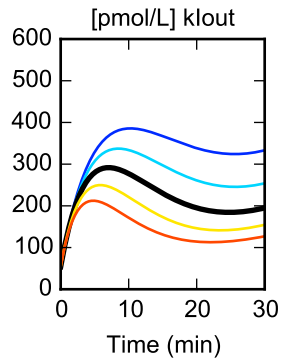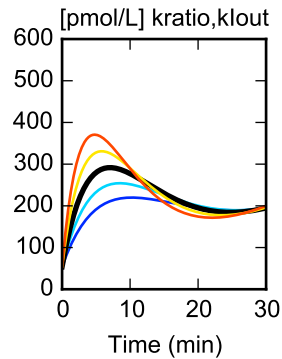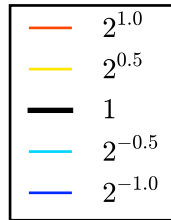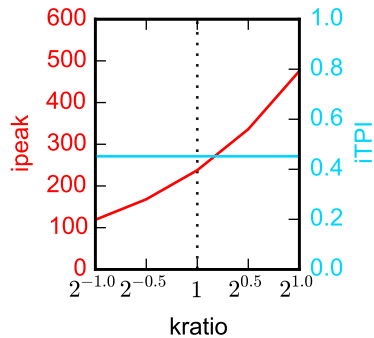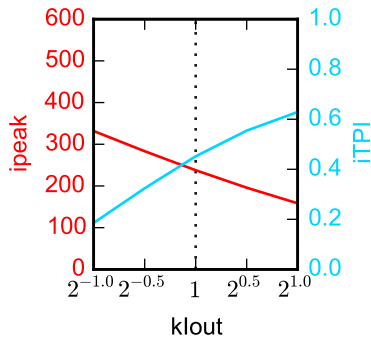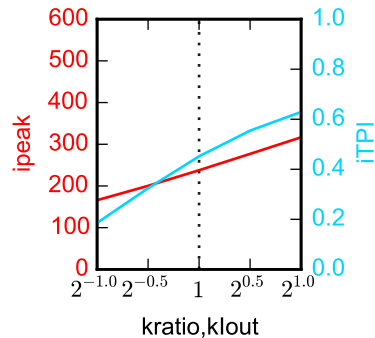

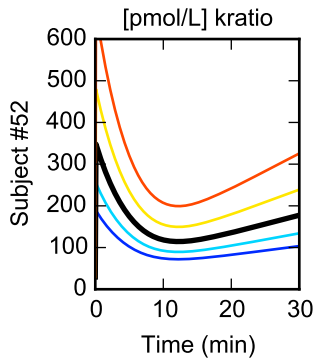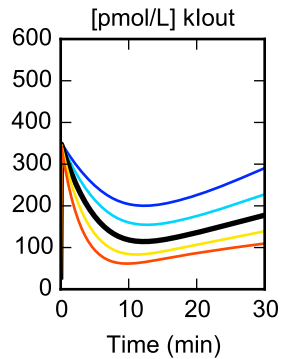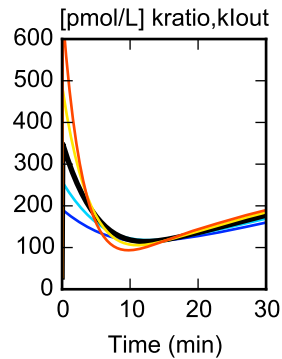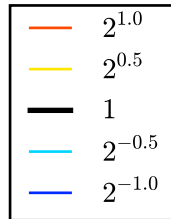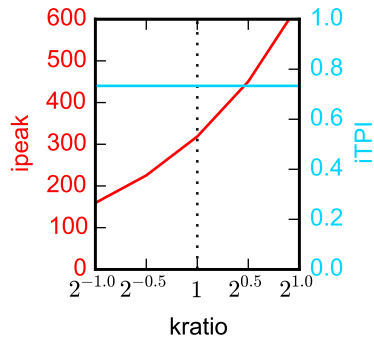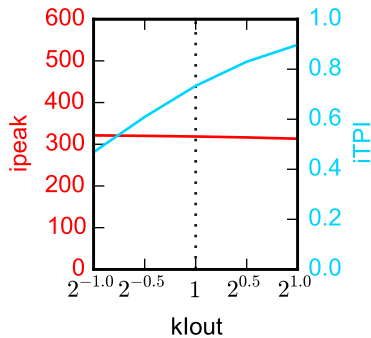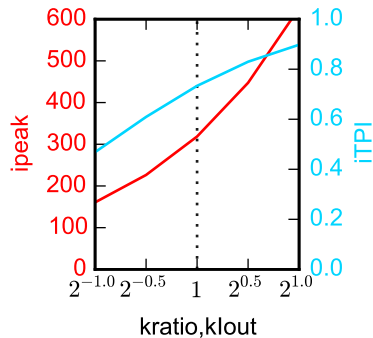

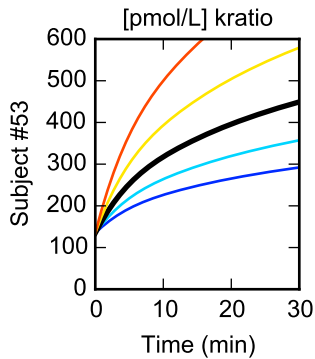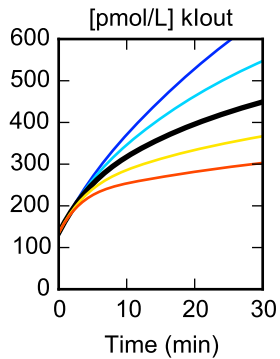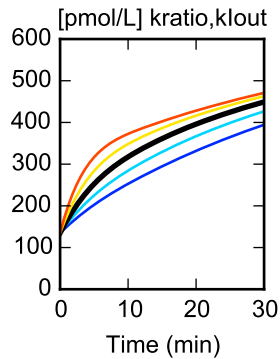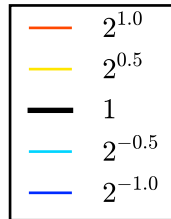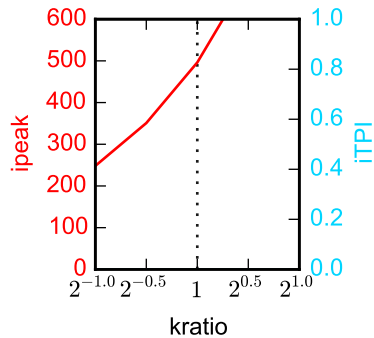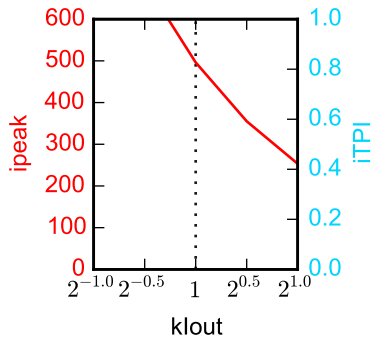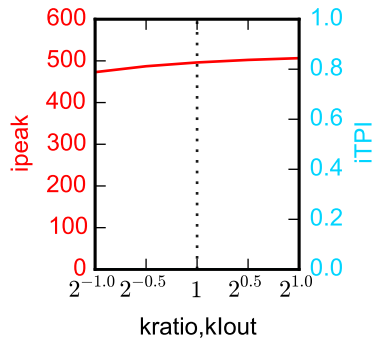

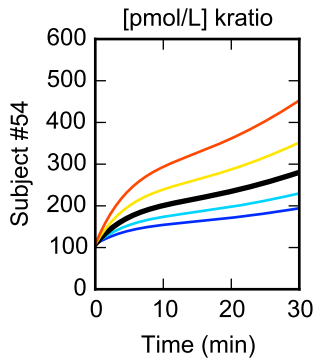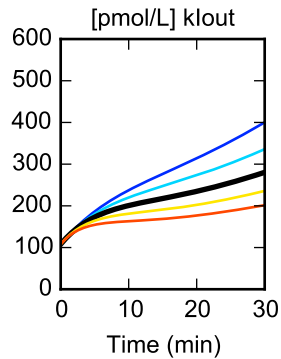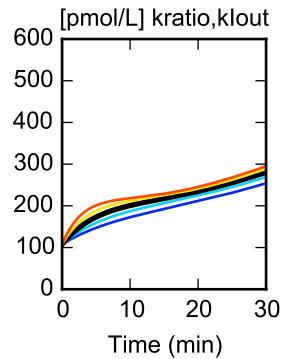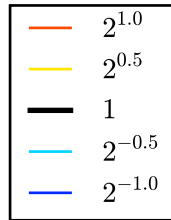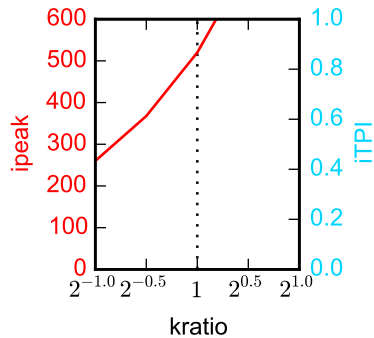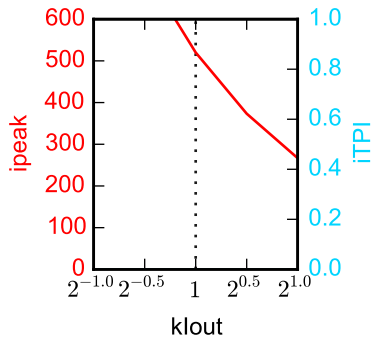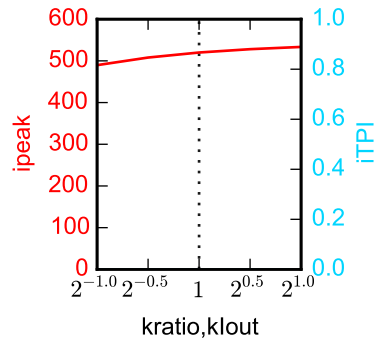

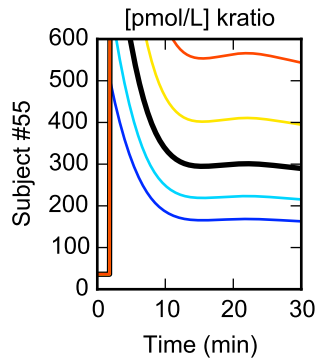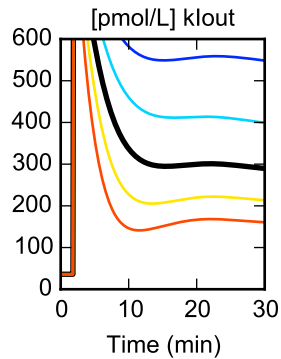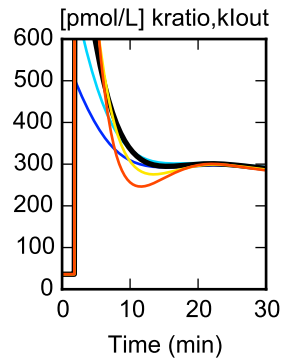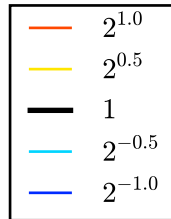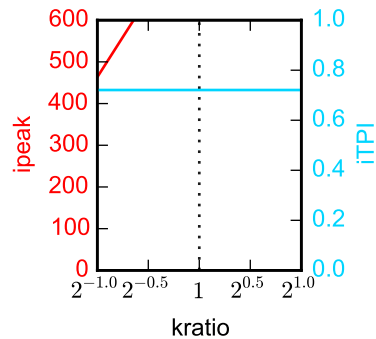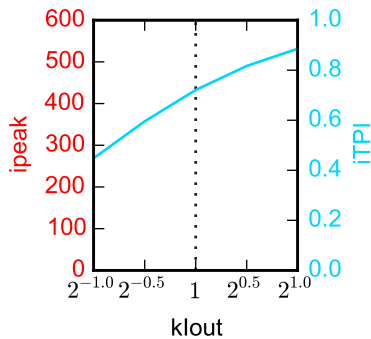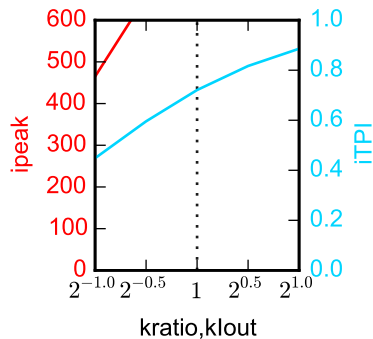

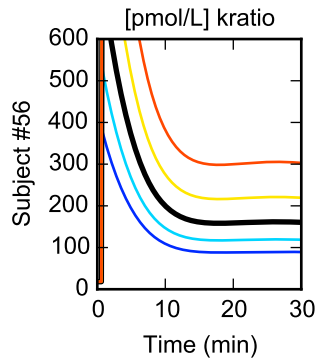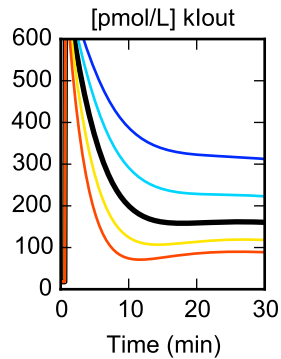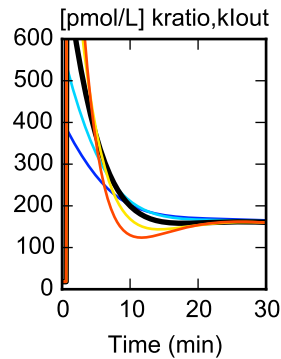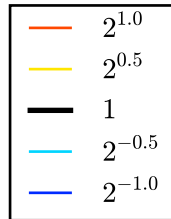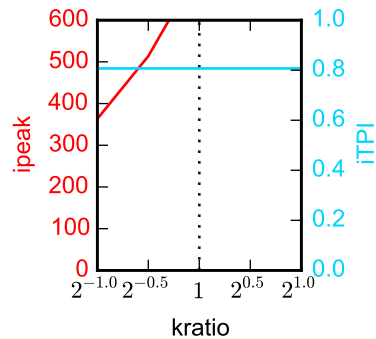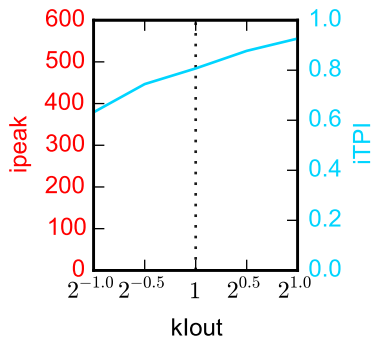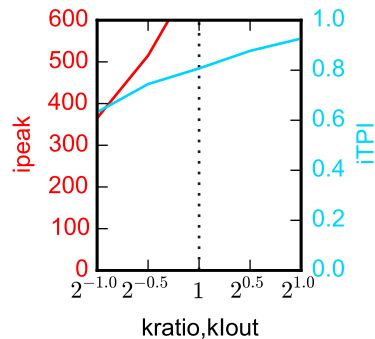

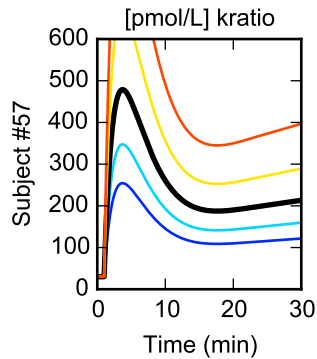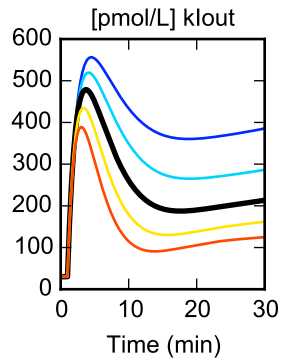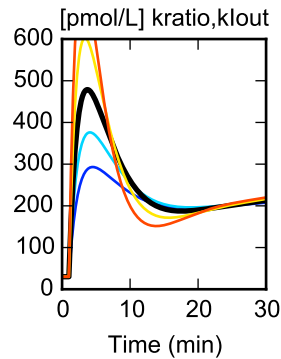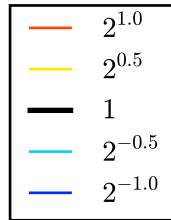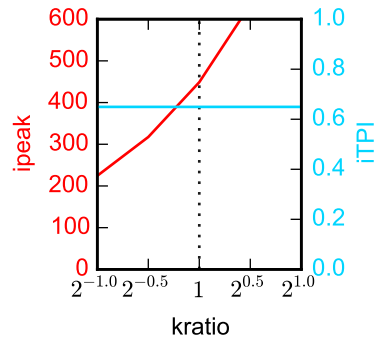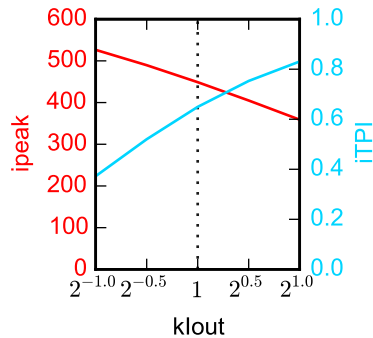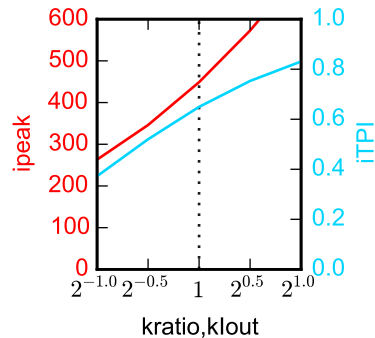

Subject #58

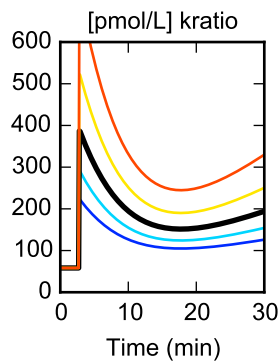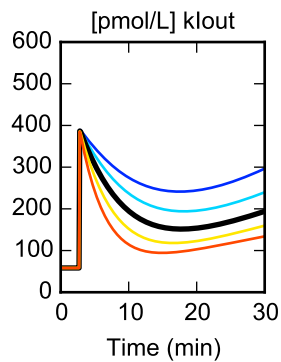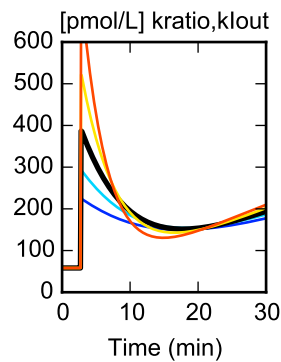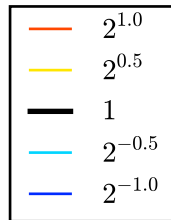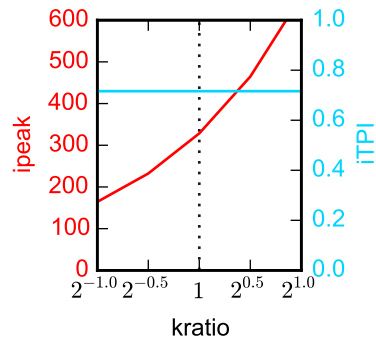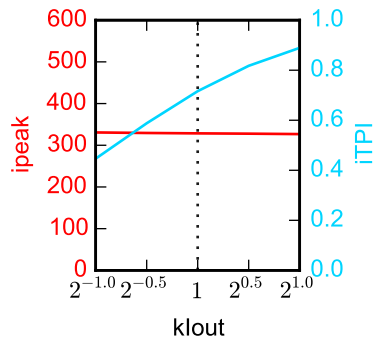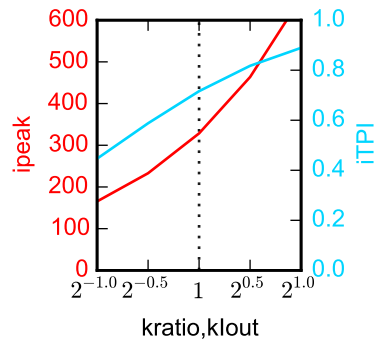

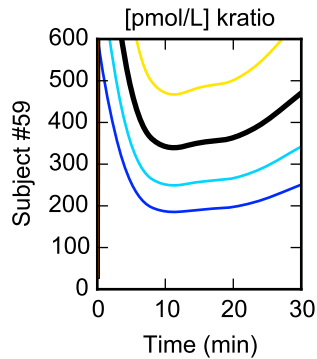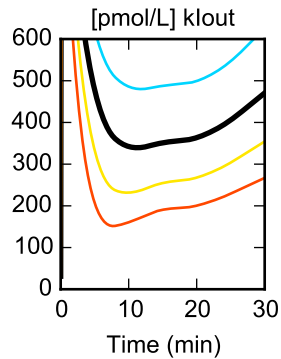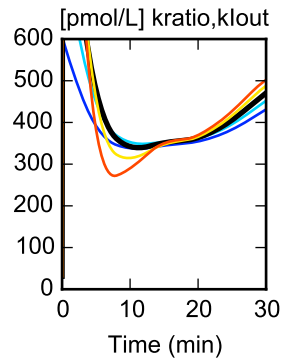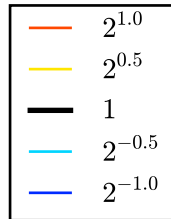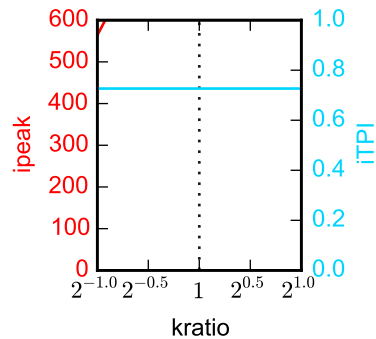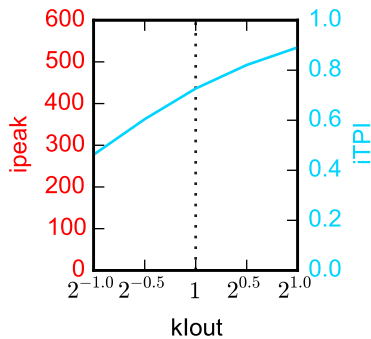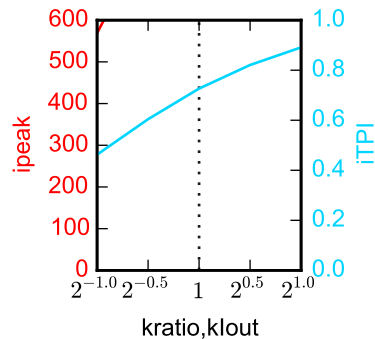

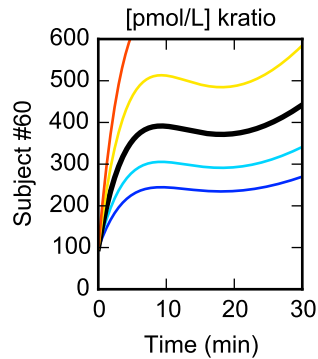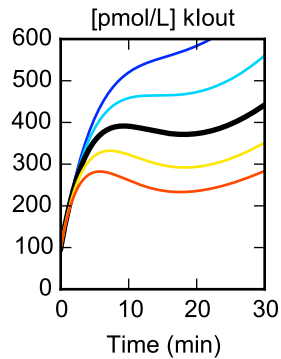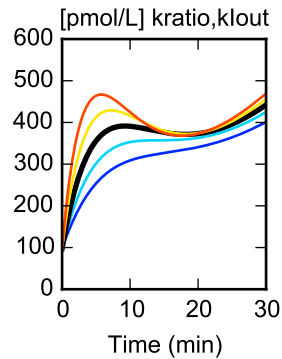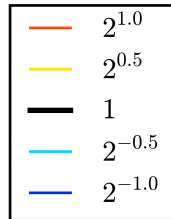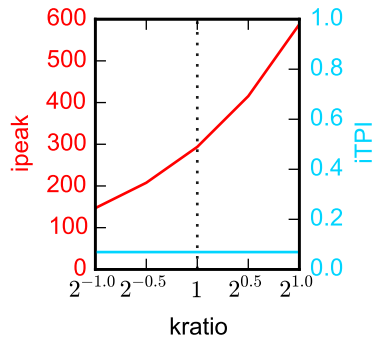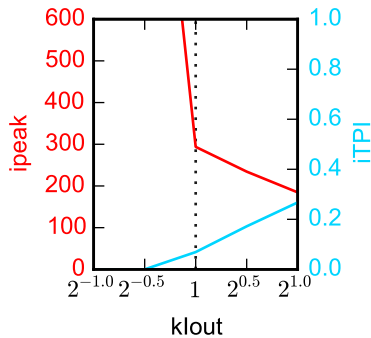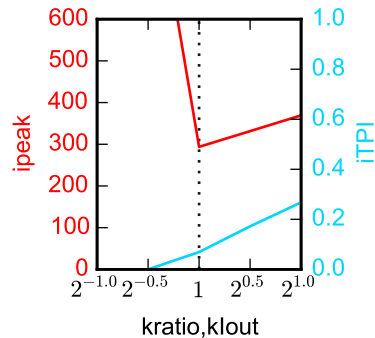

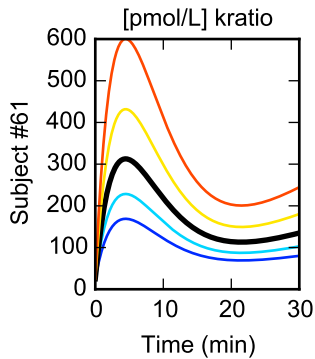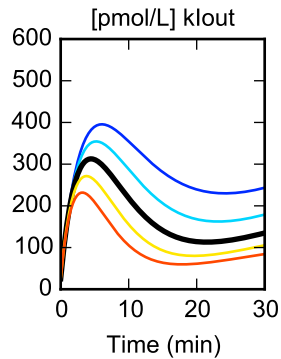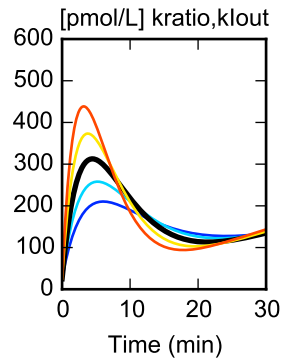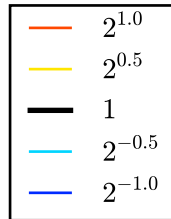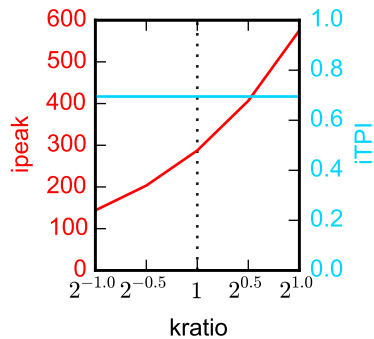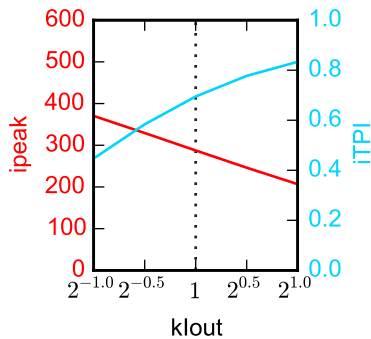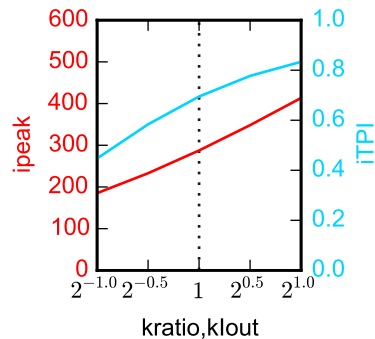

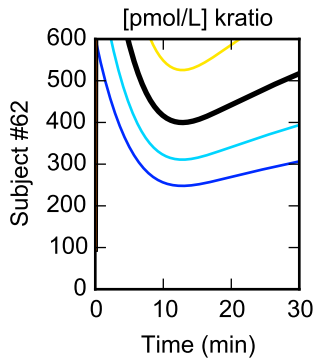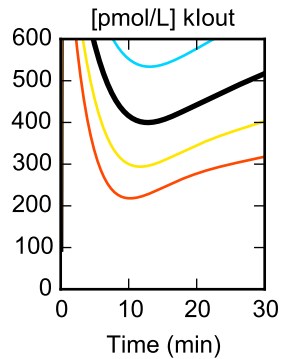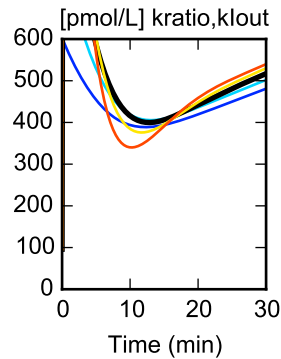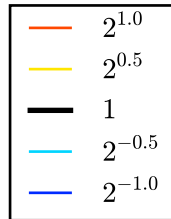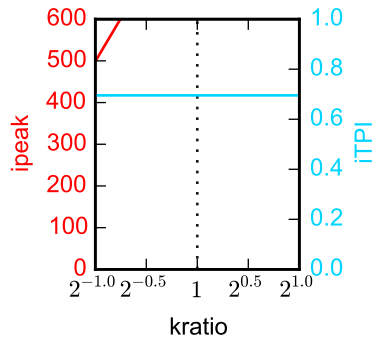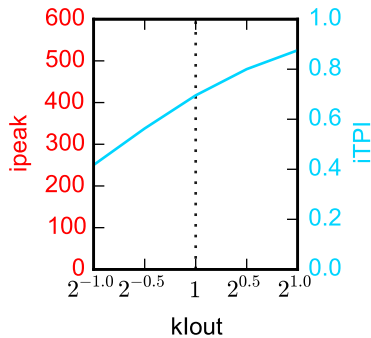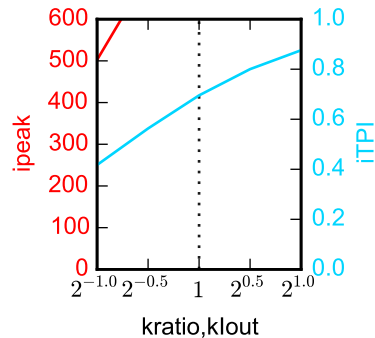

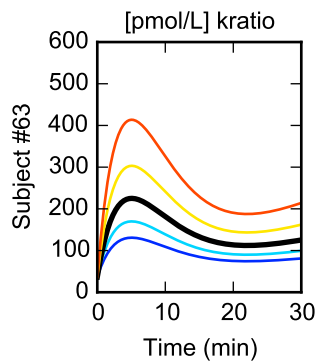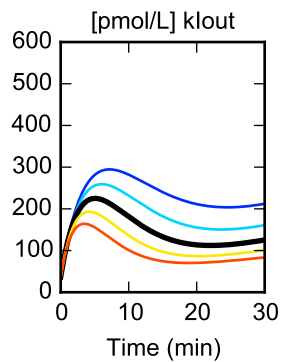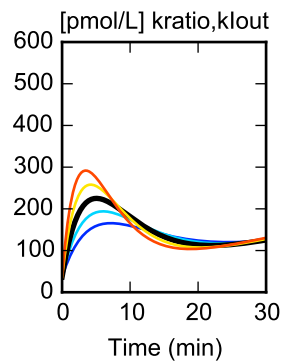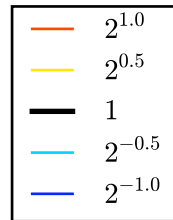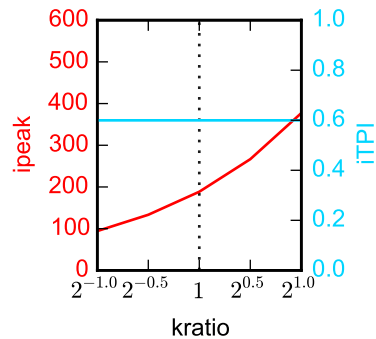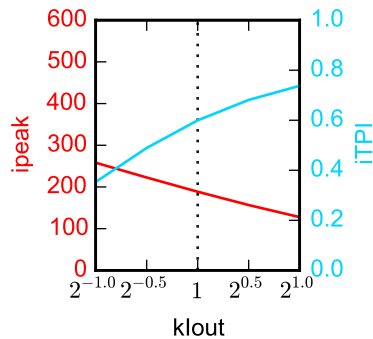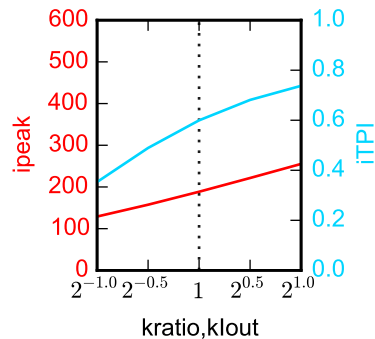

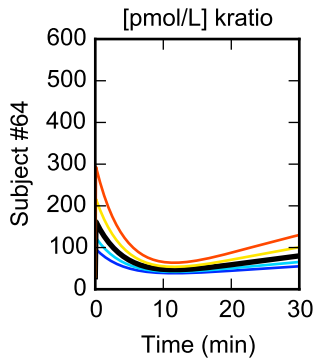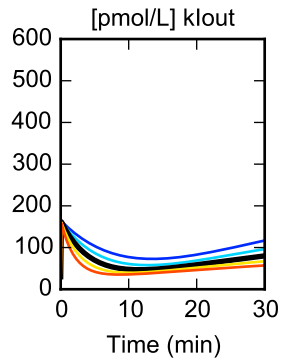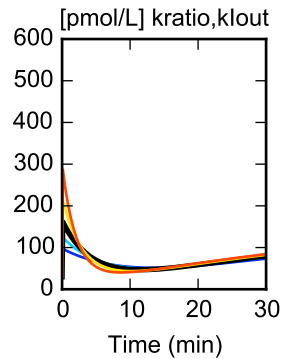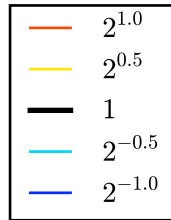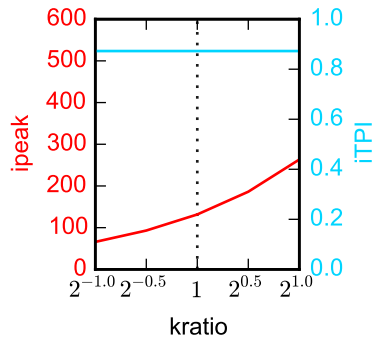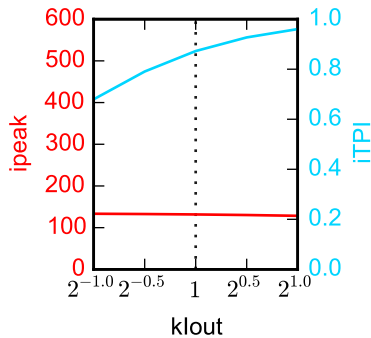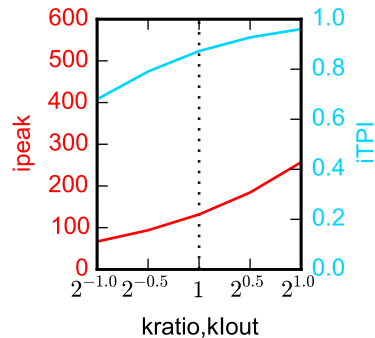

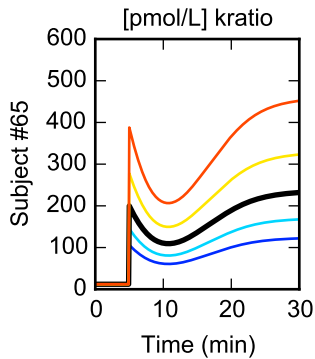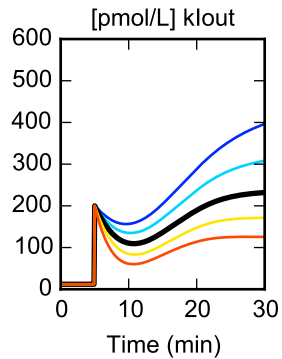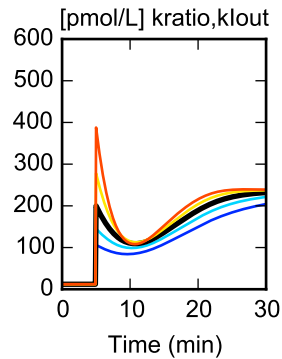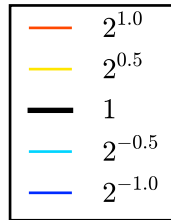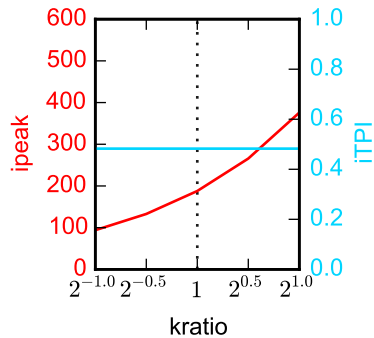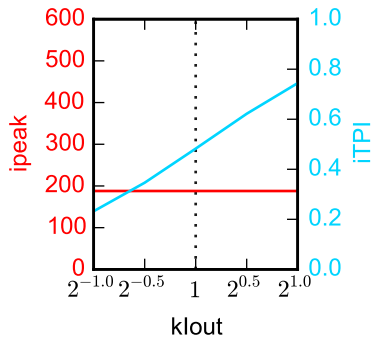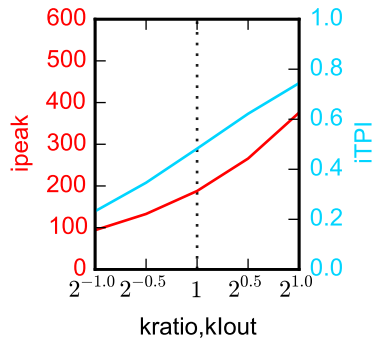

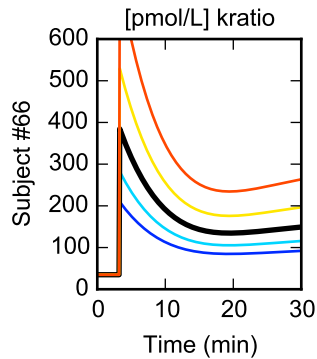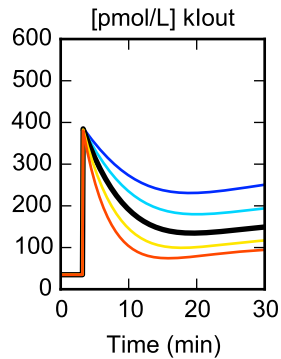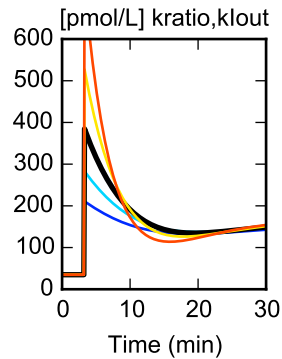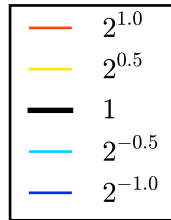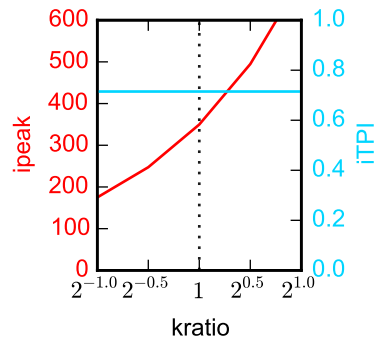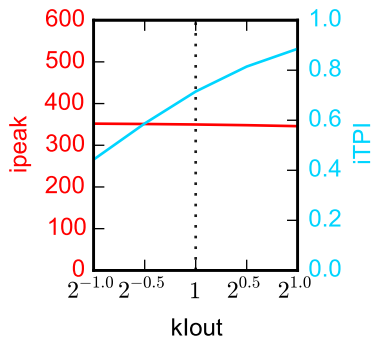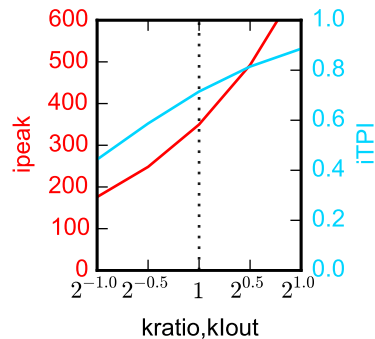

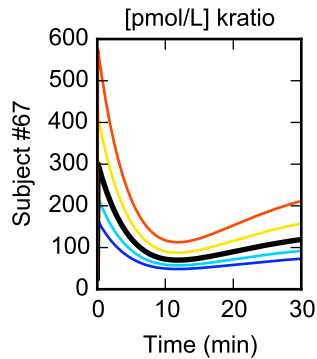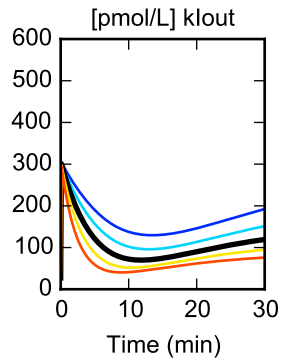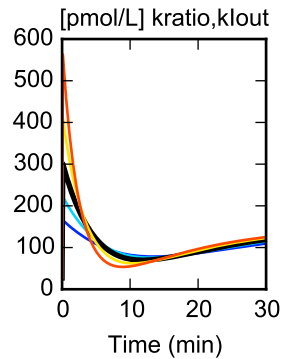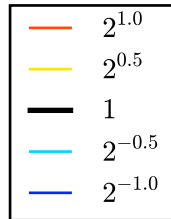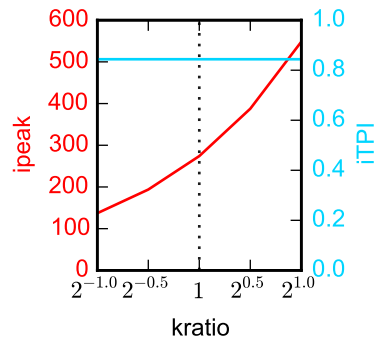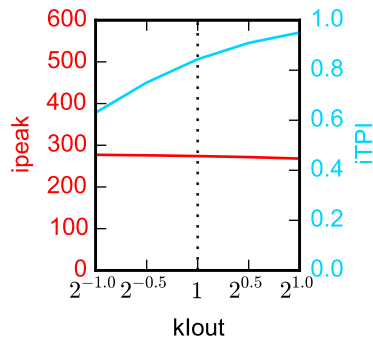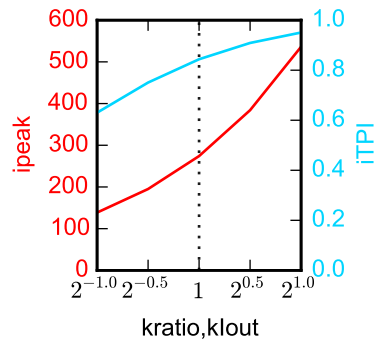

Subject #68

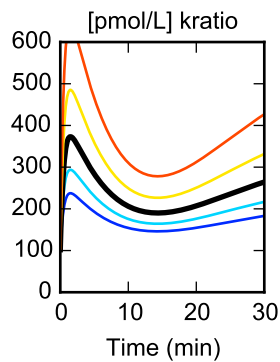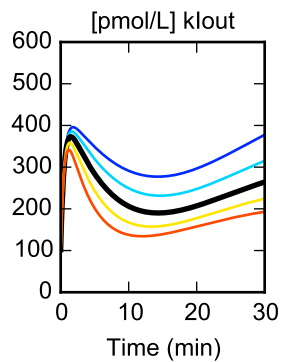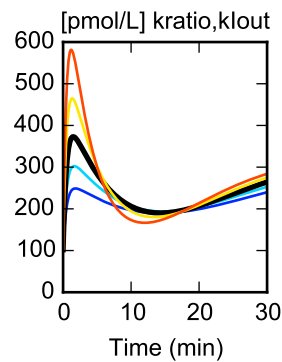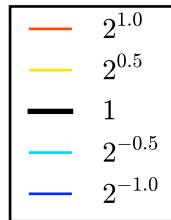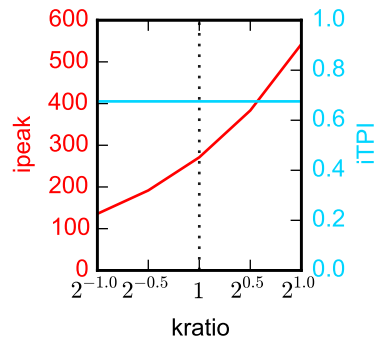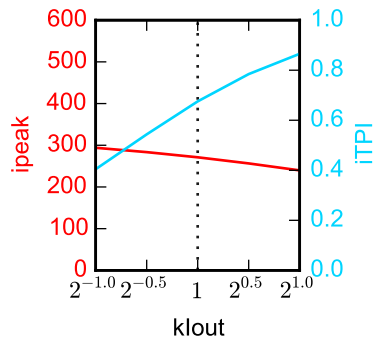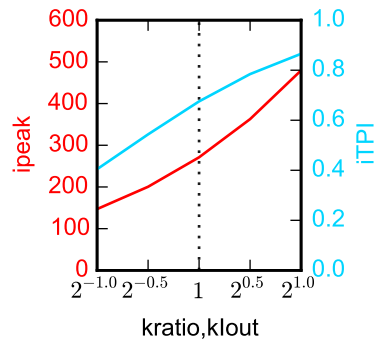

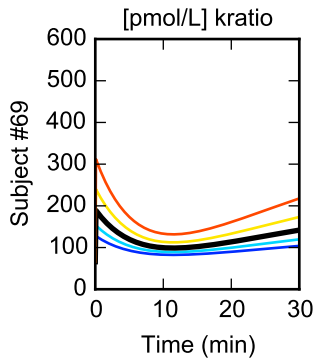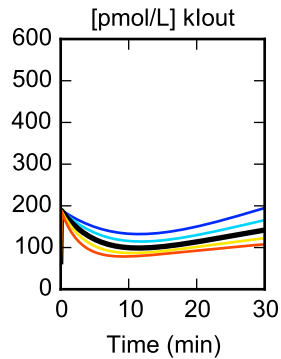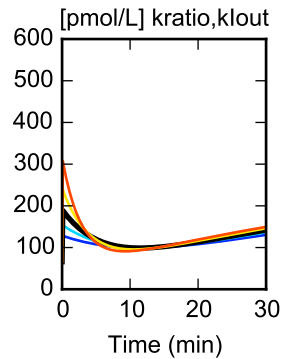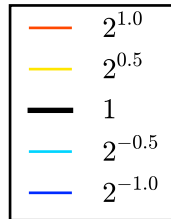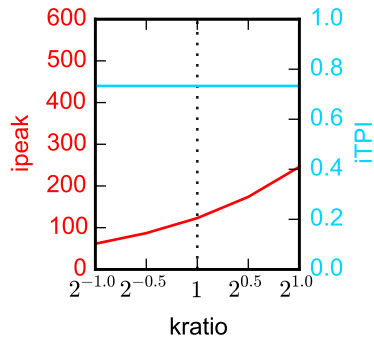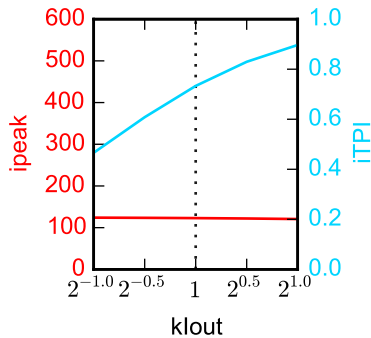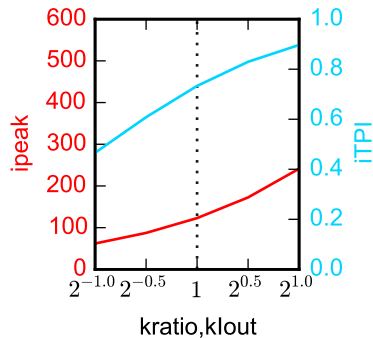

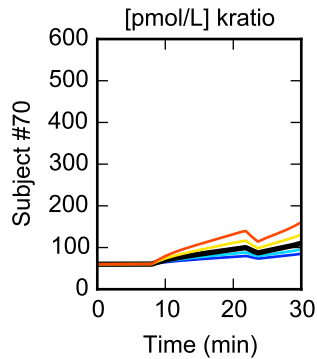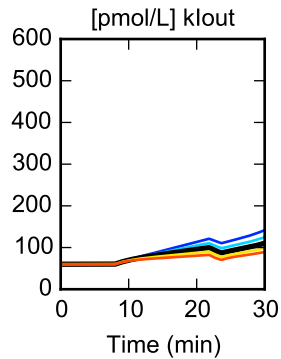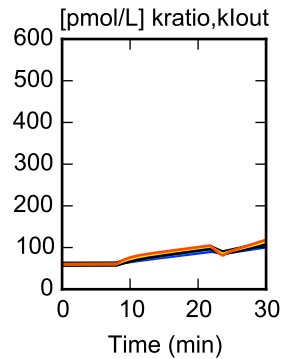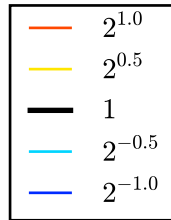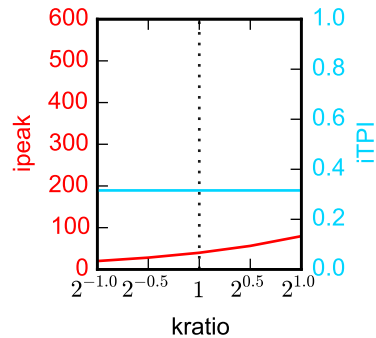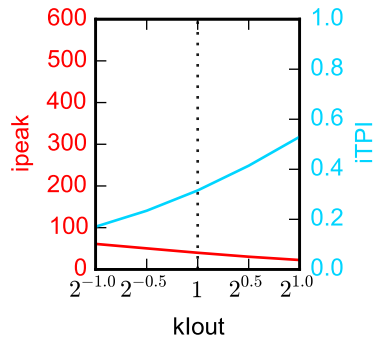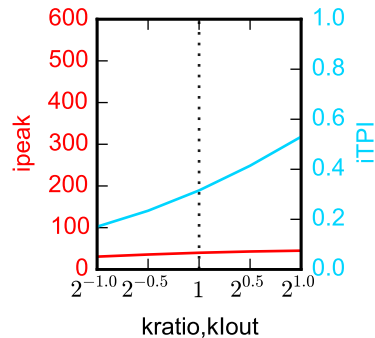

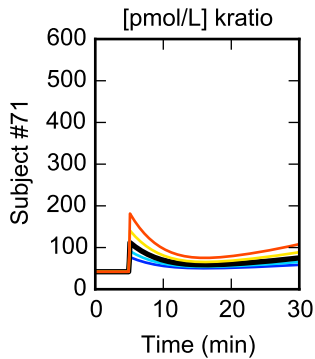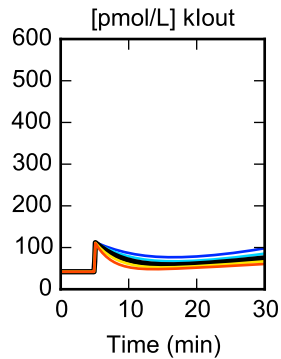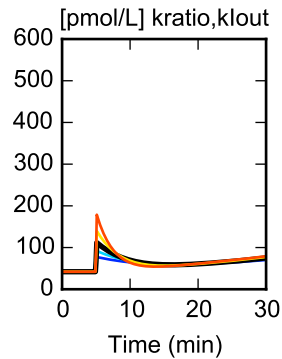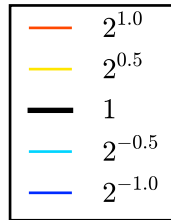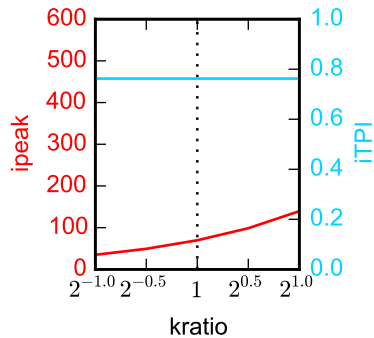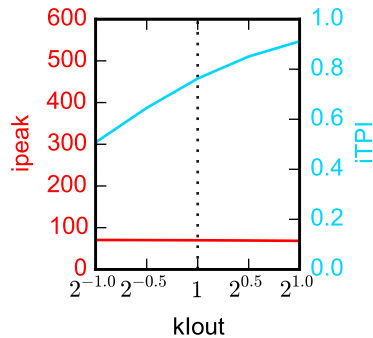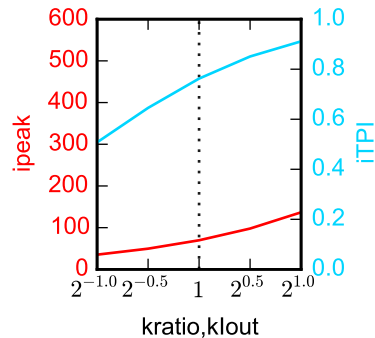

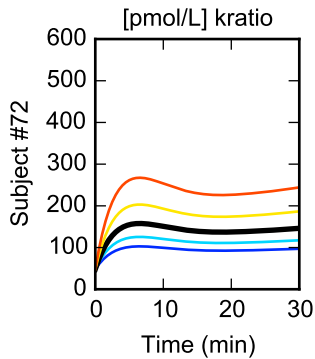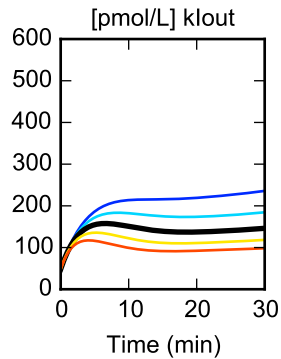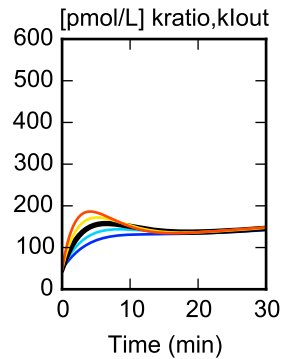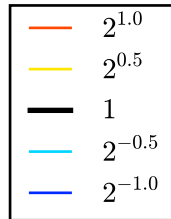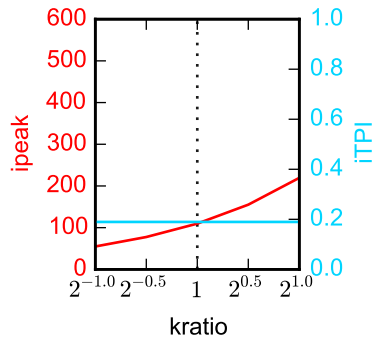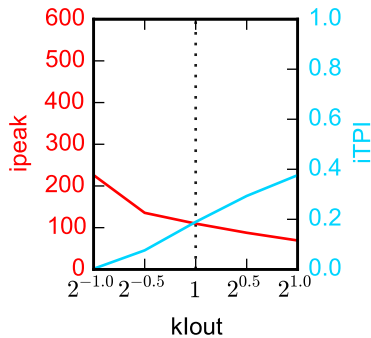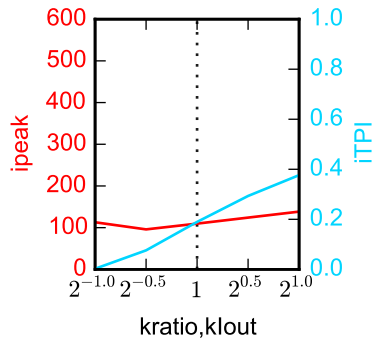

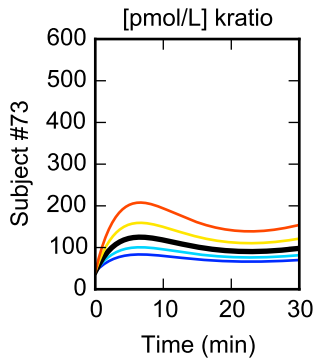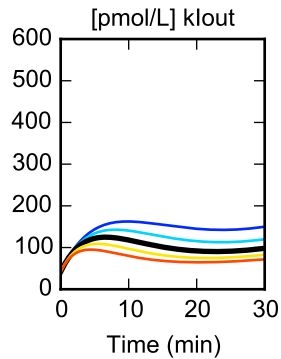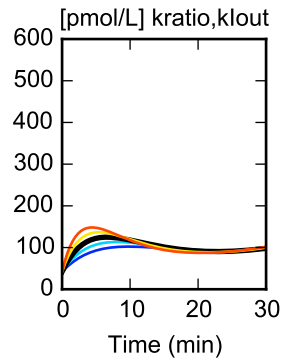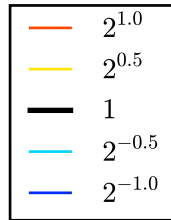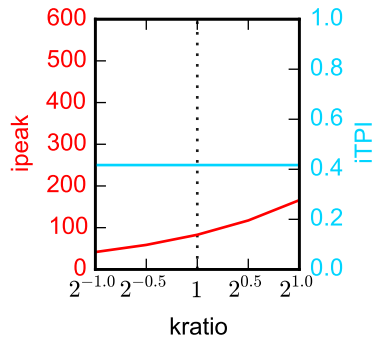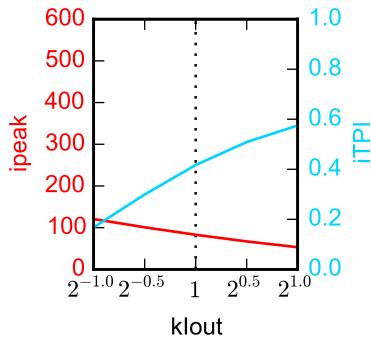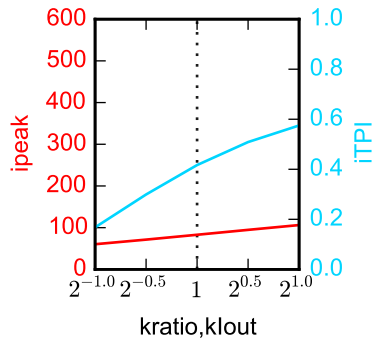

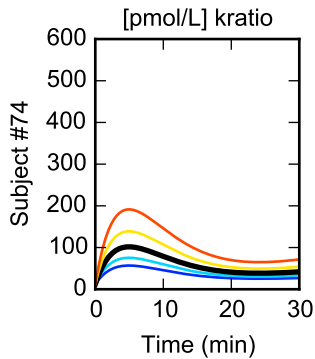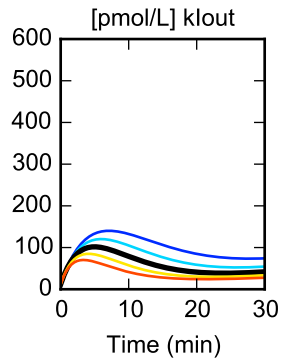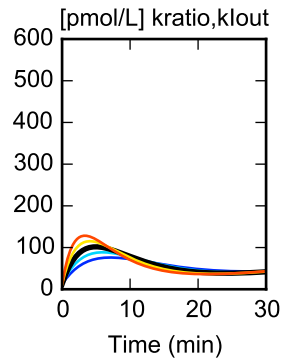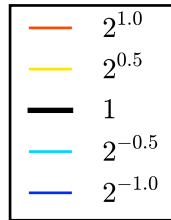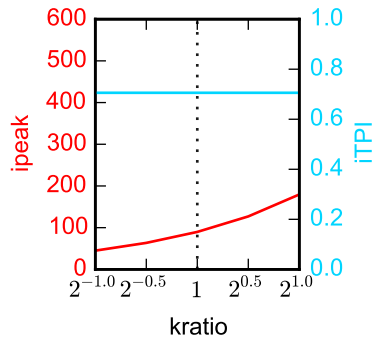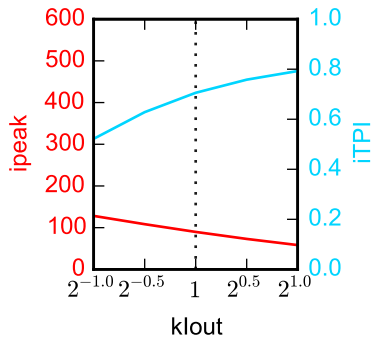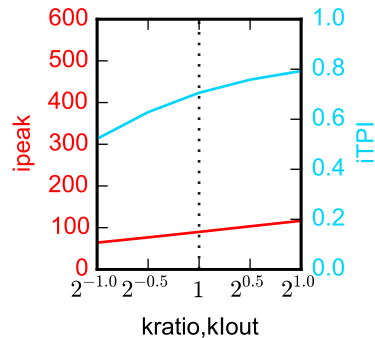

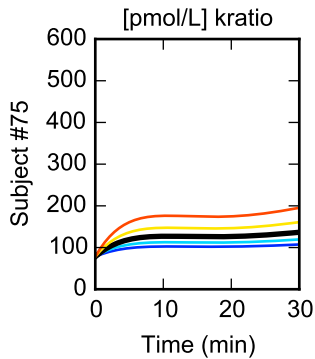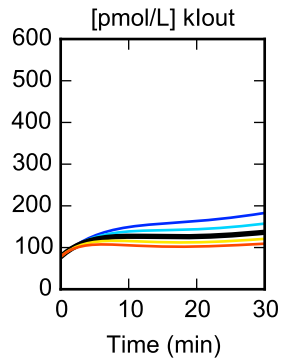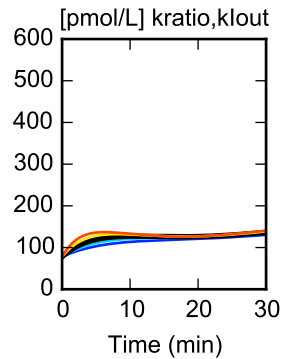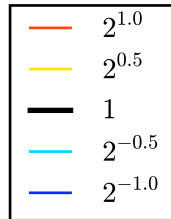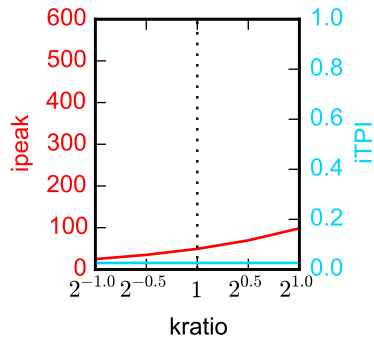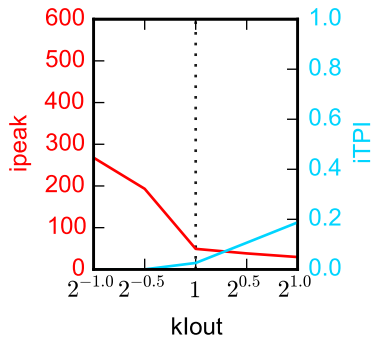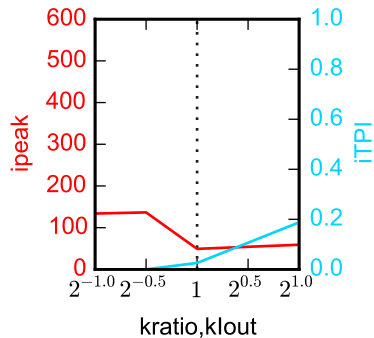

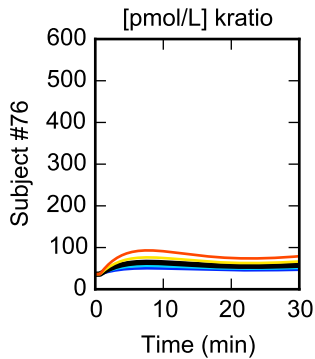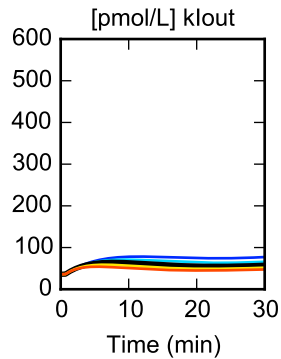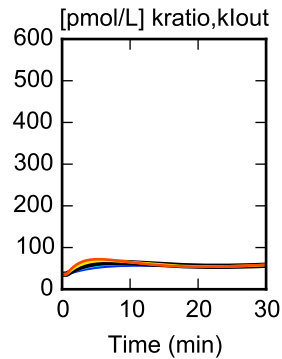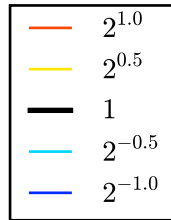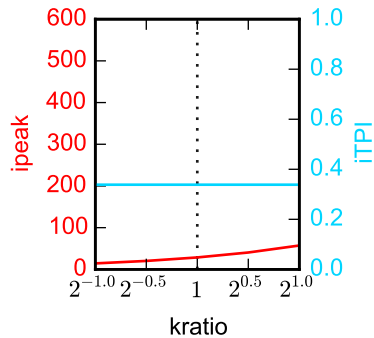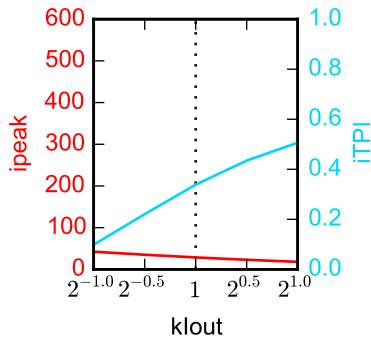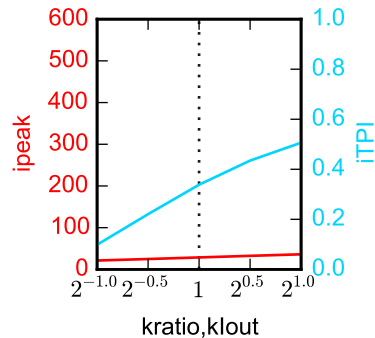

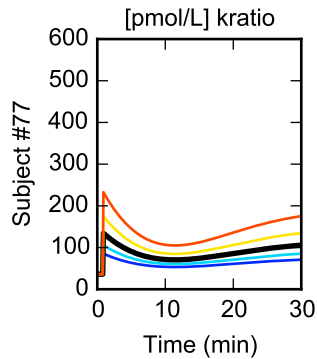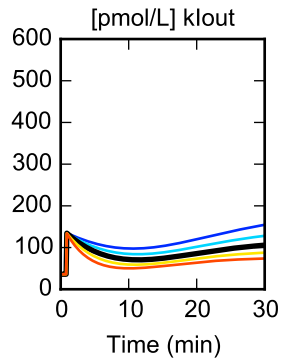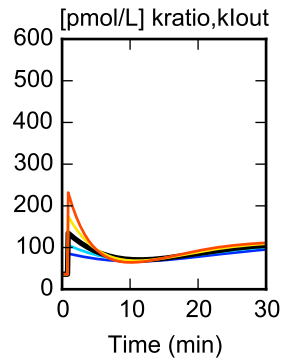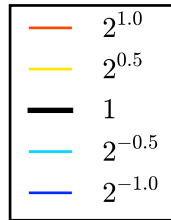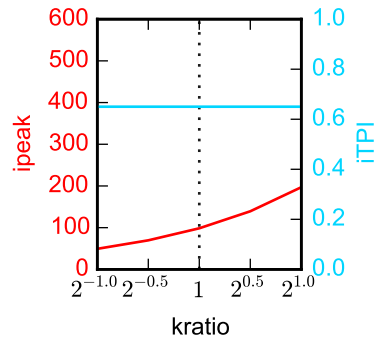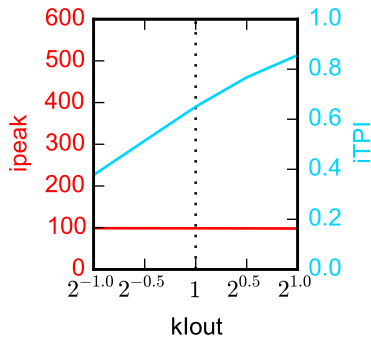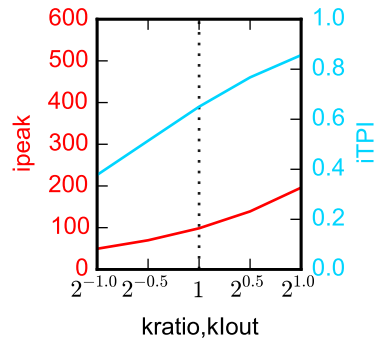

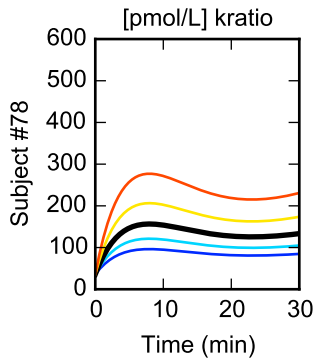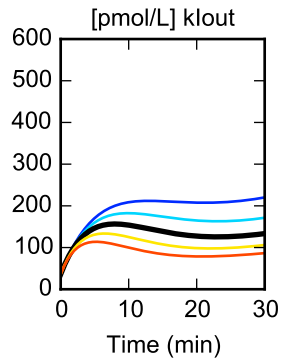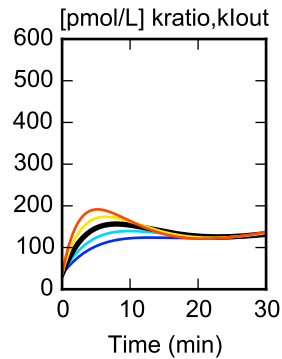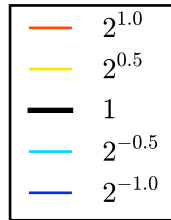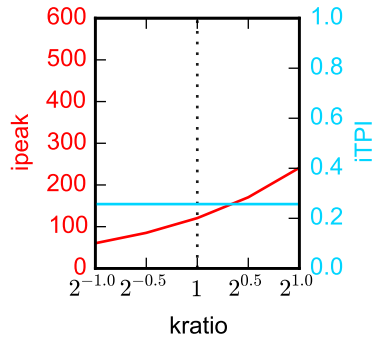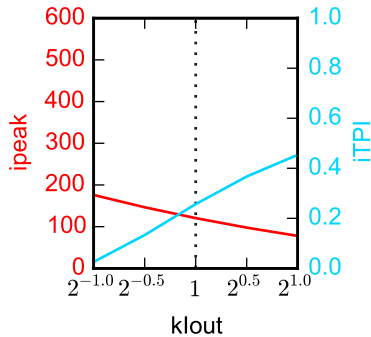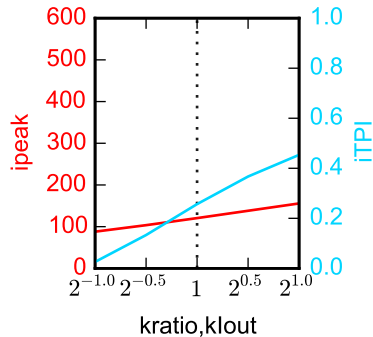

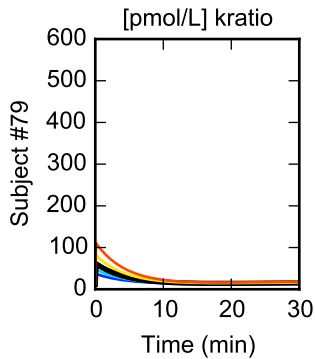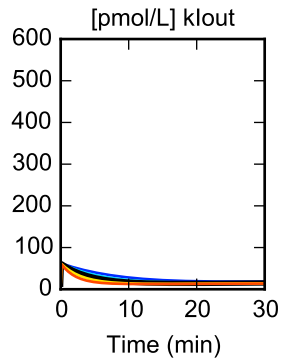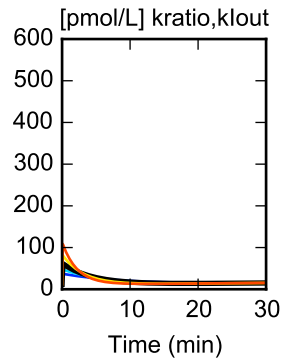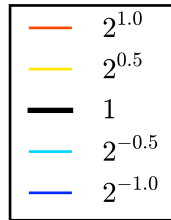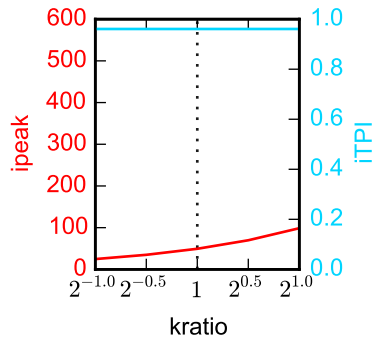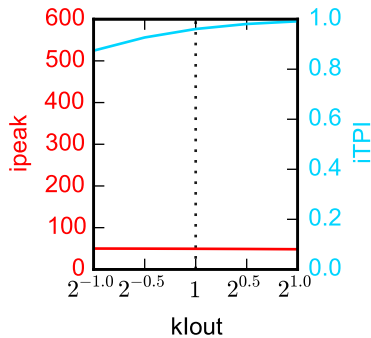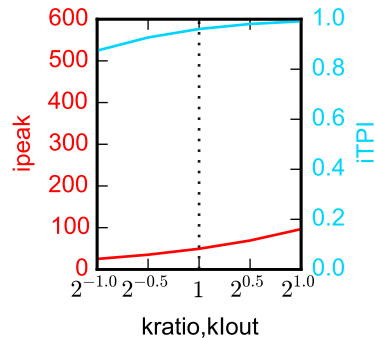

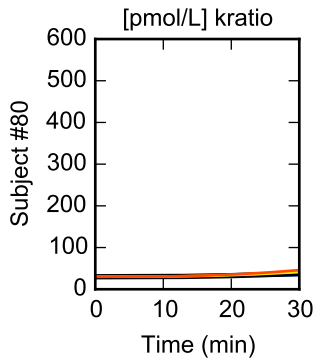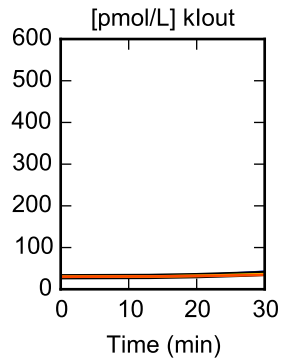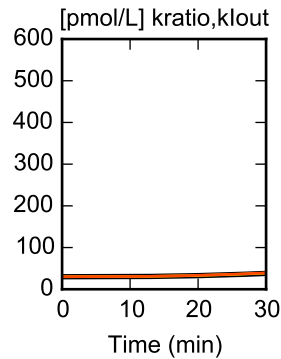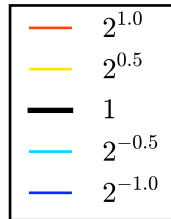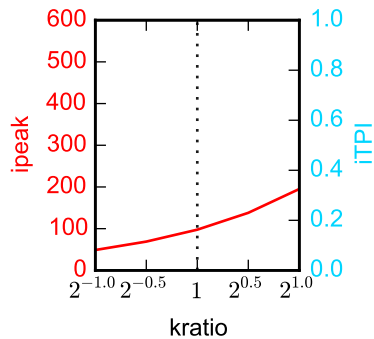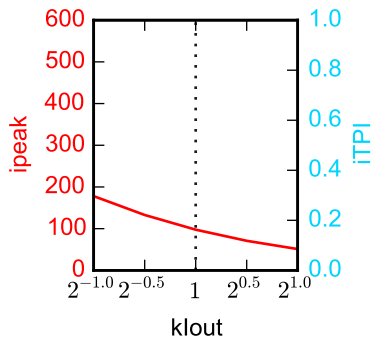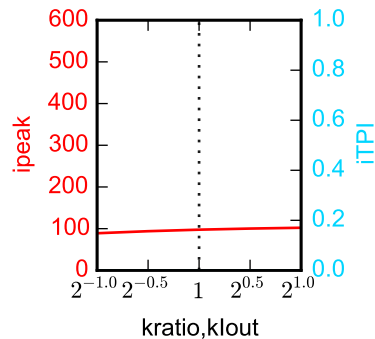

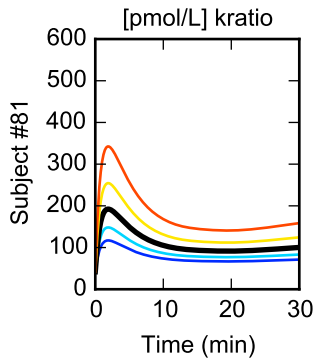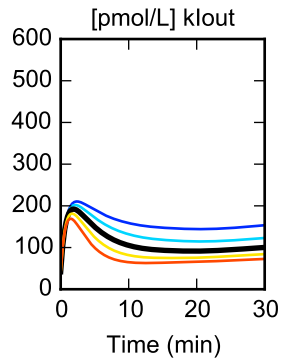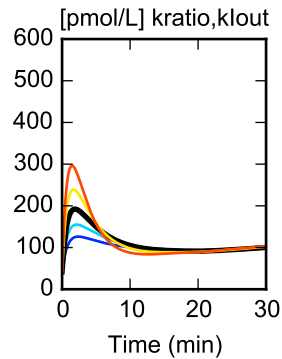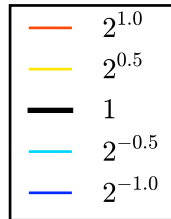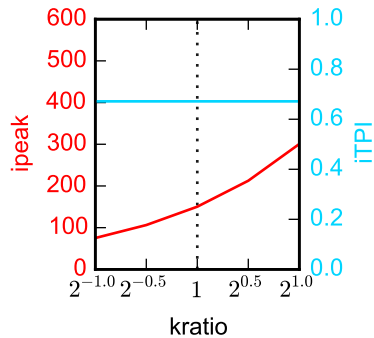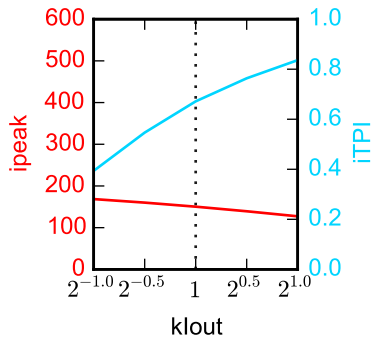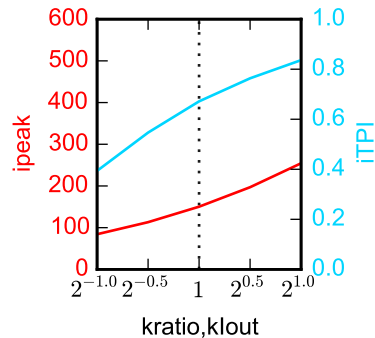

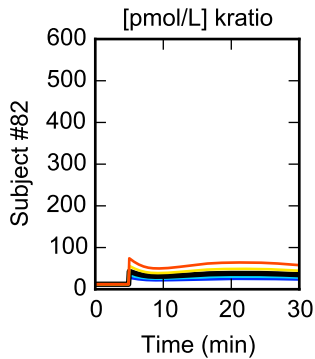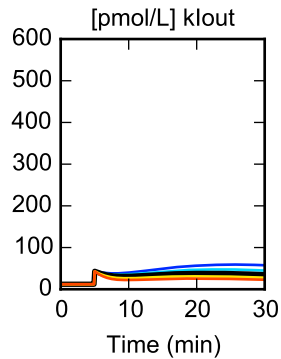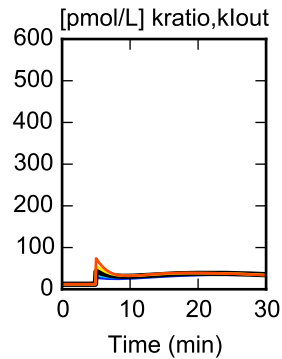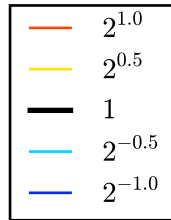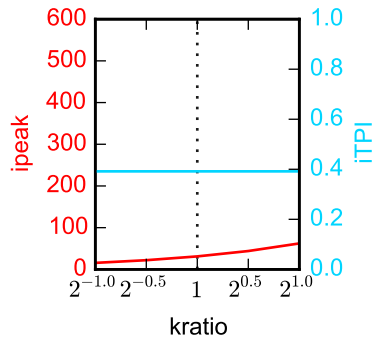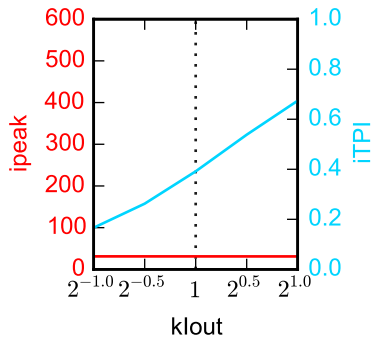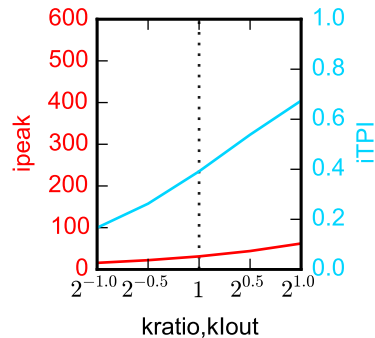

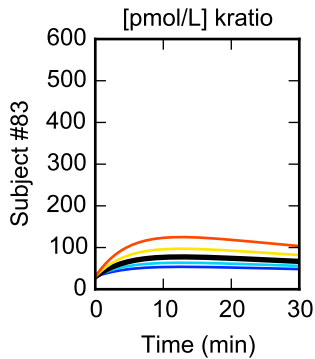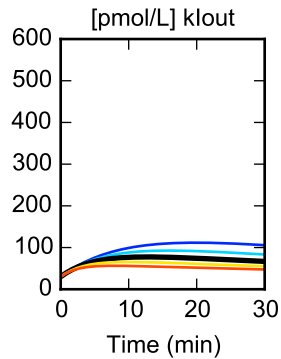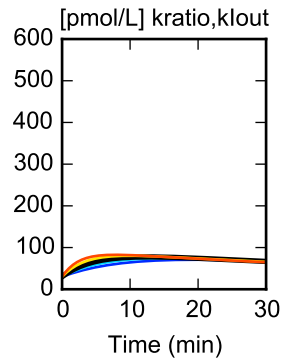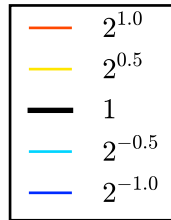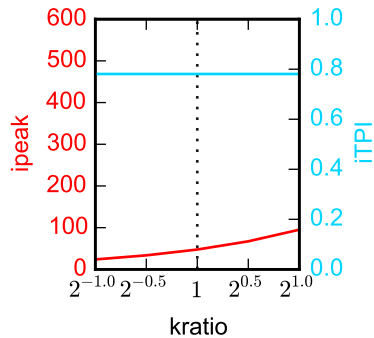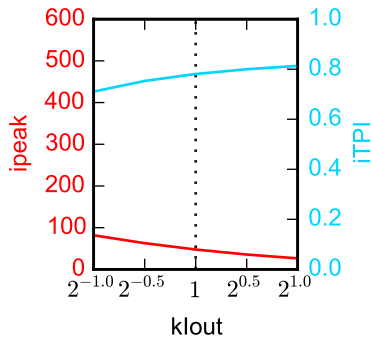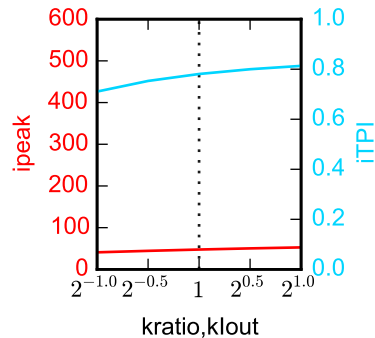

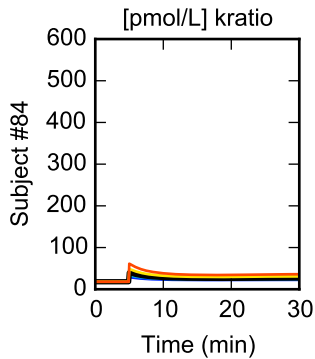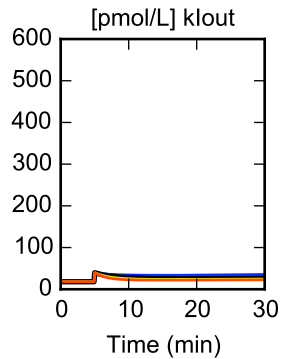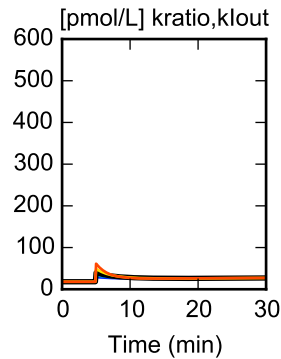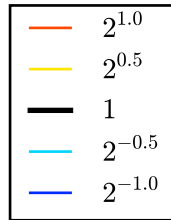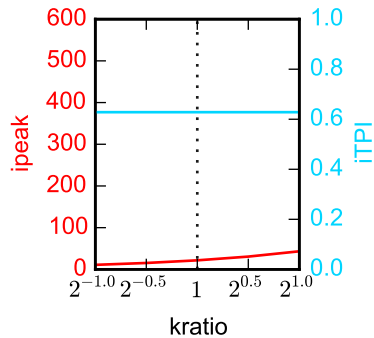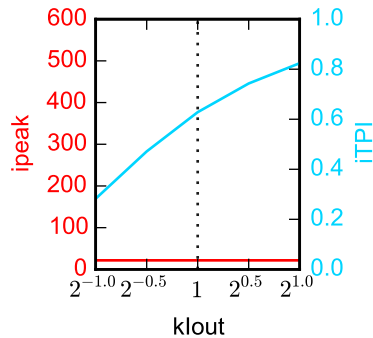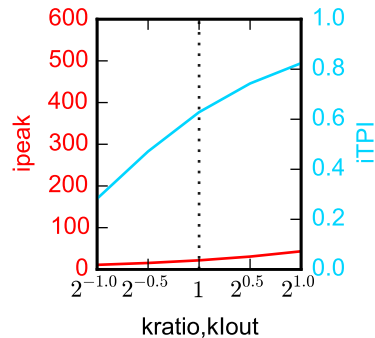

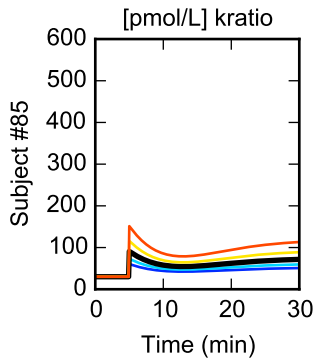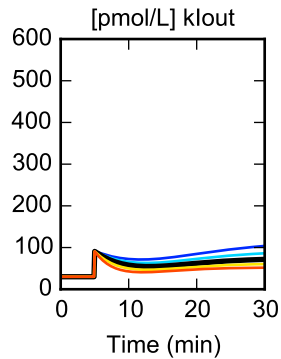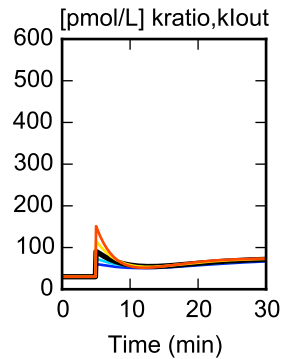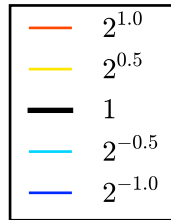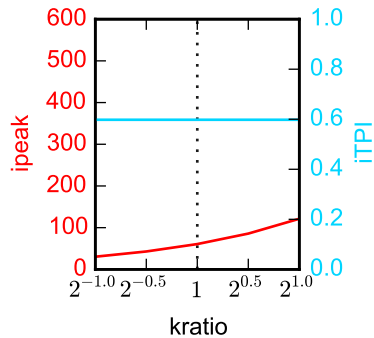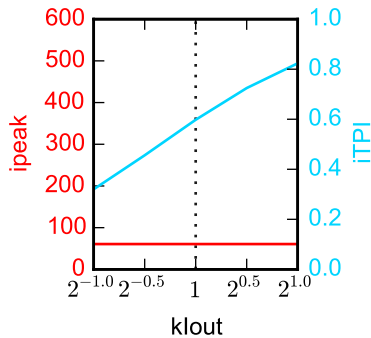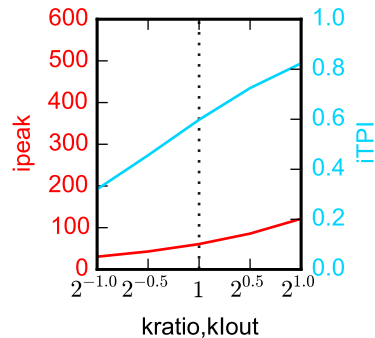

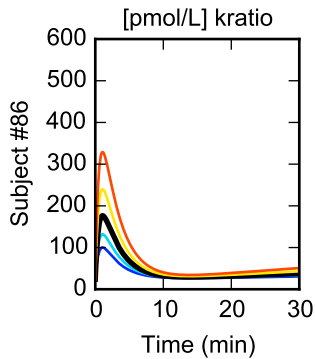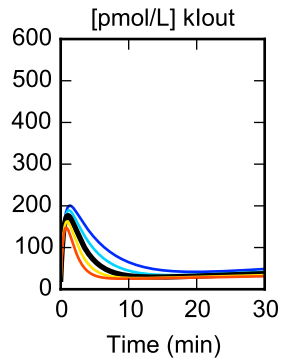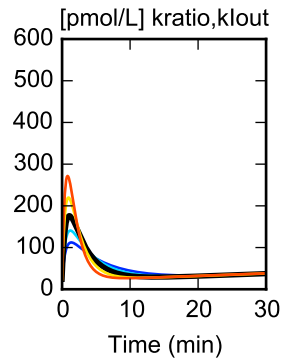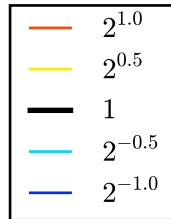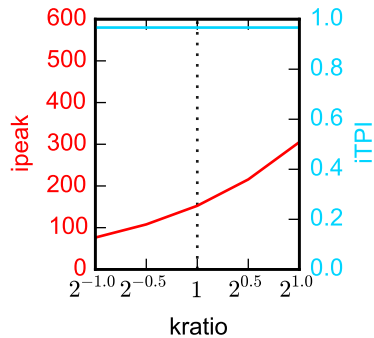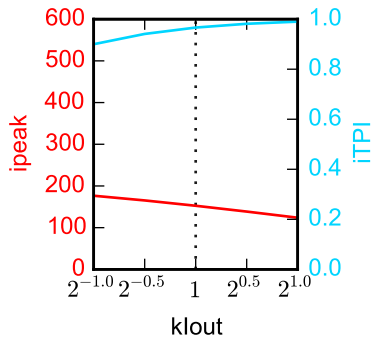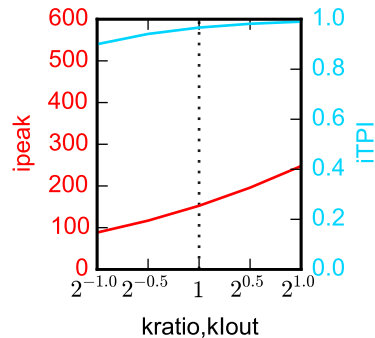

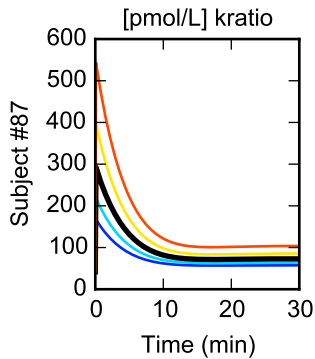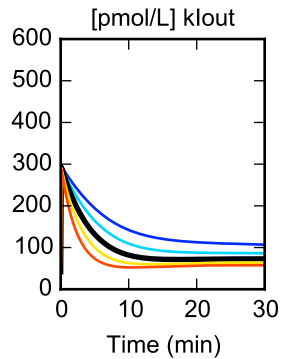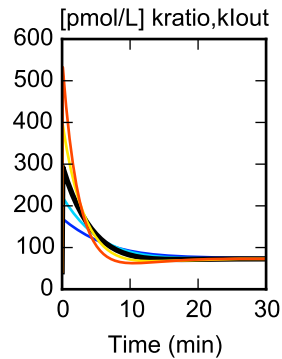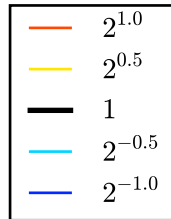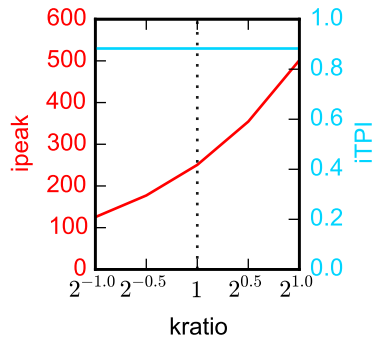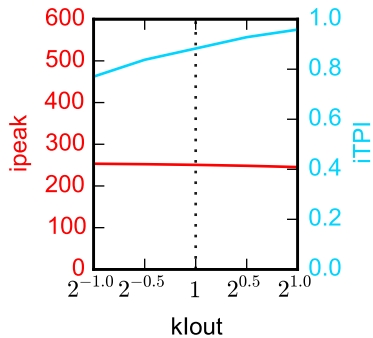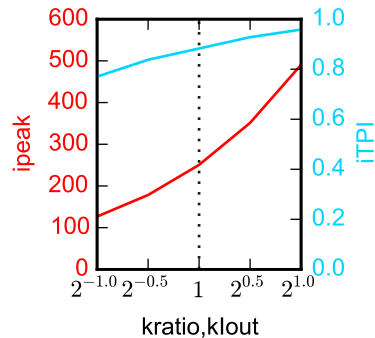

Subject #88

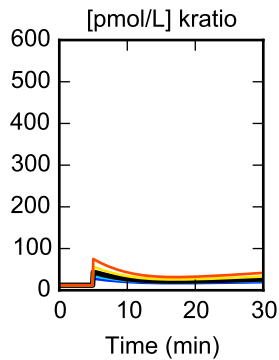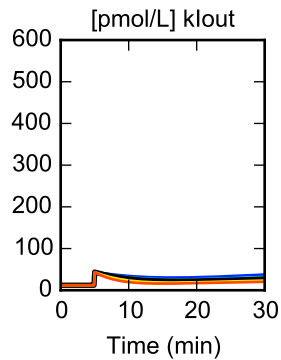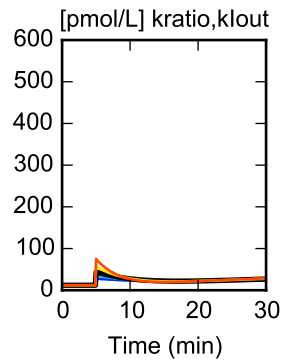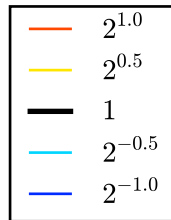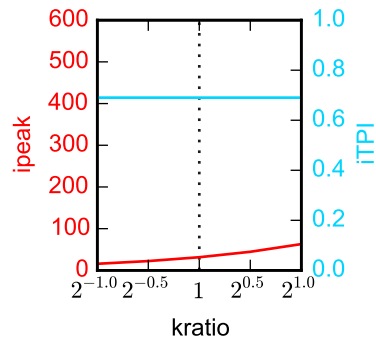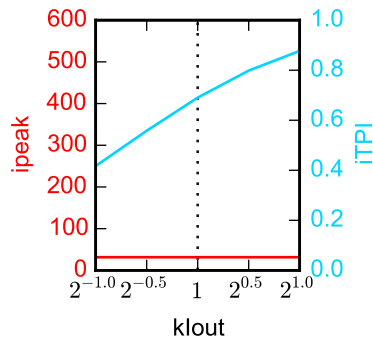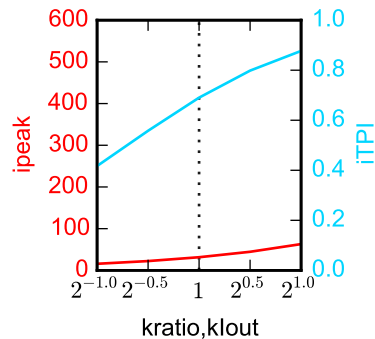

Subject #89

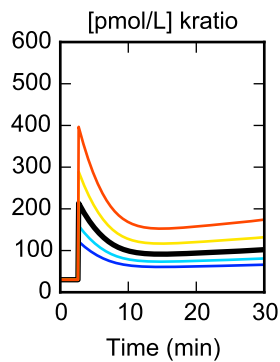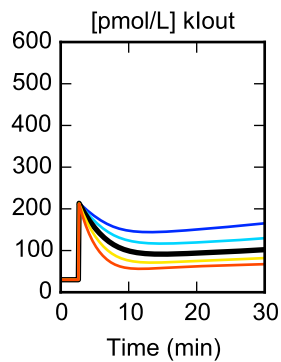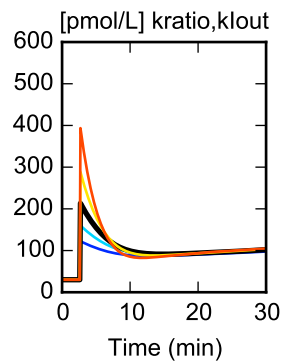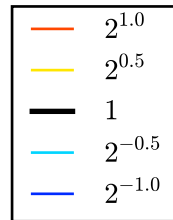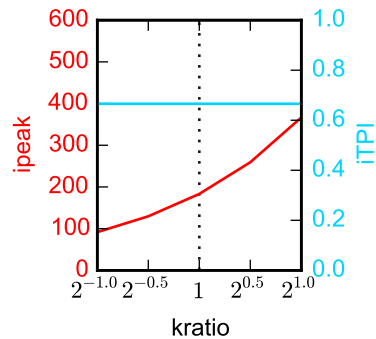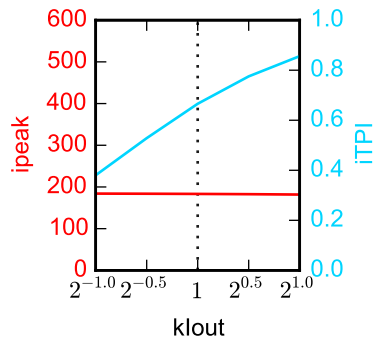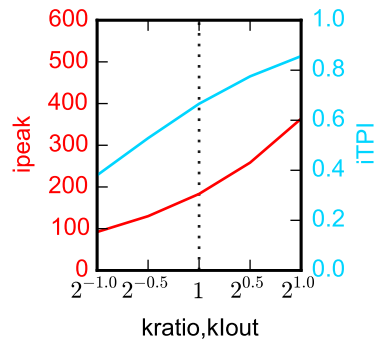

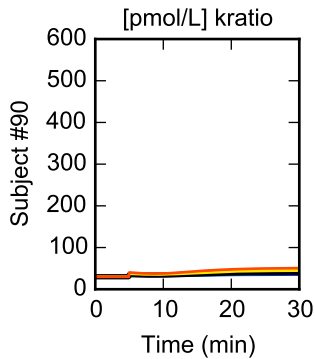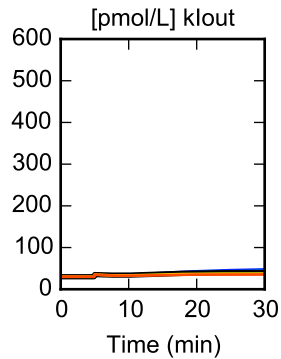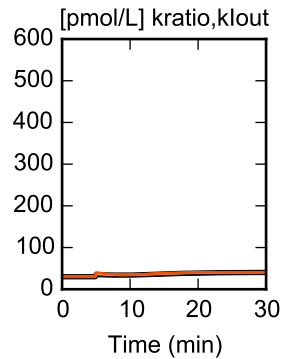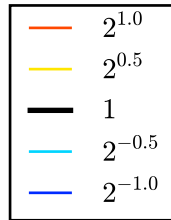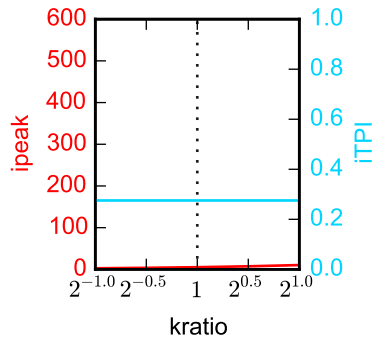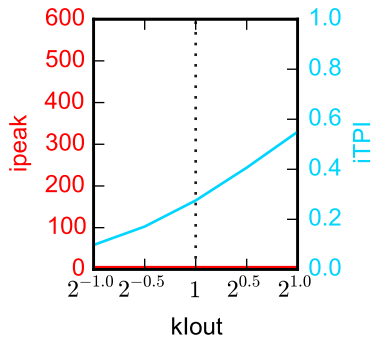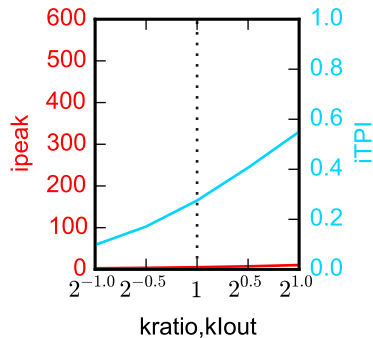

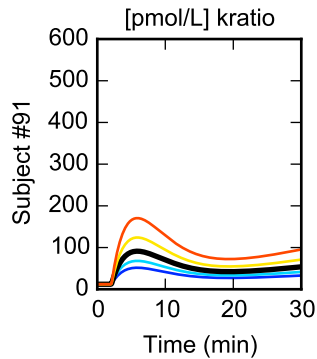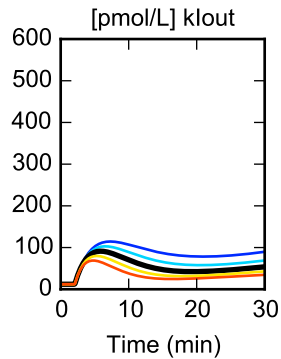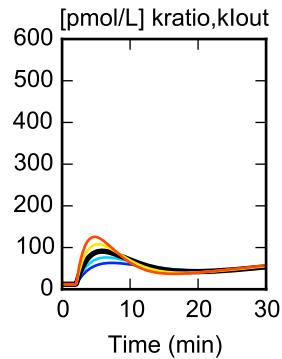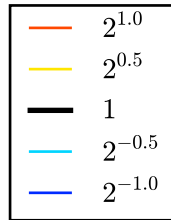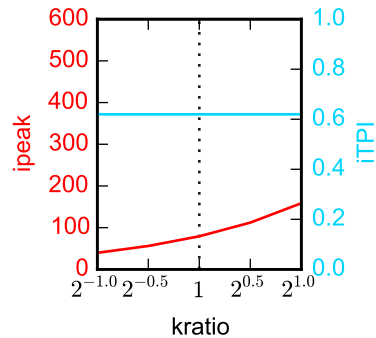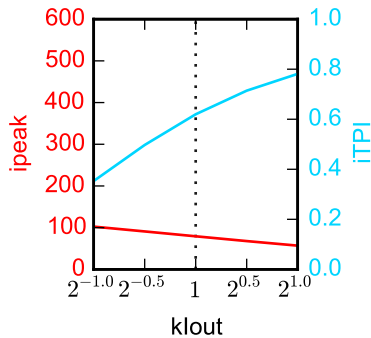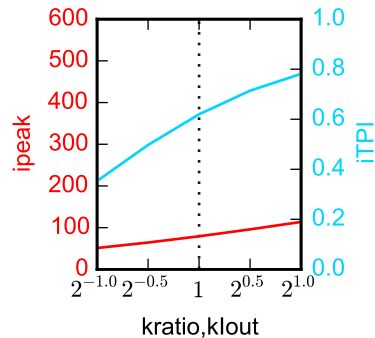

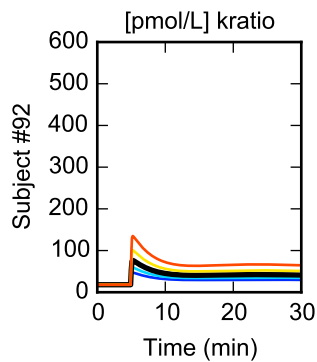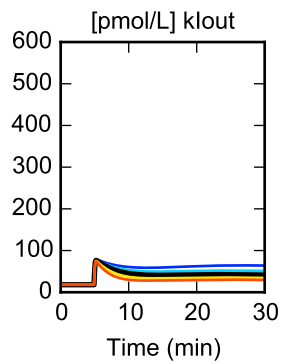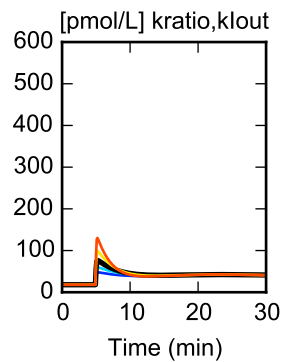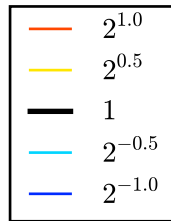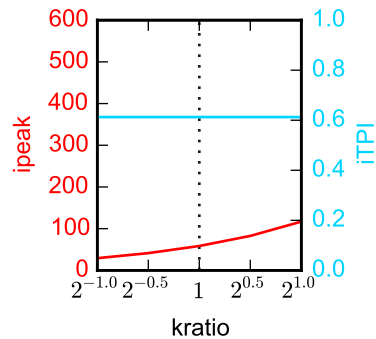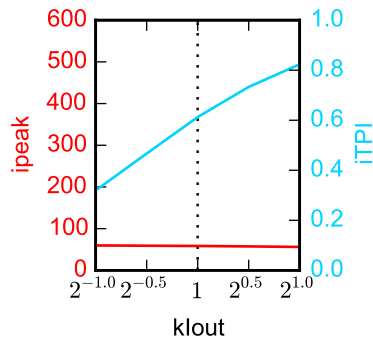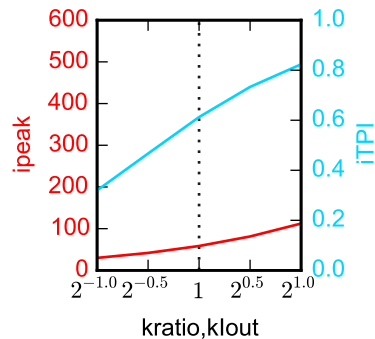

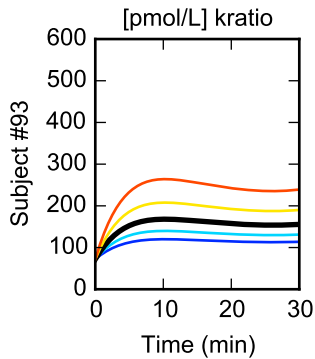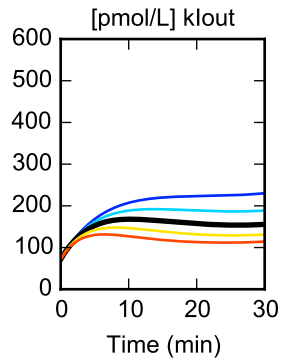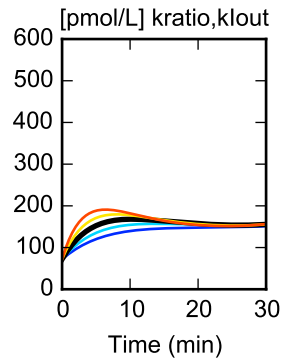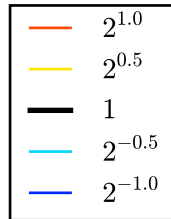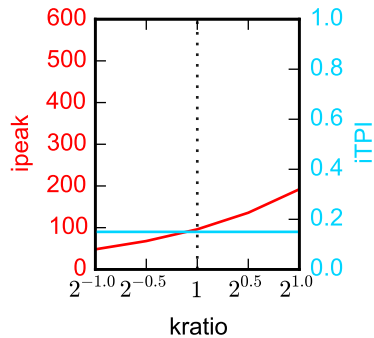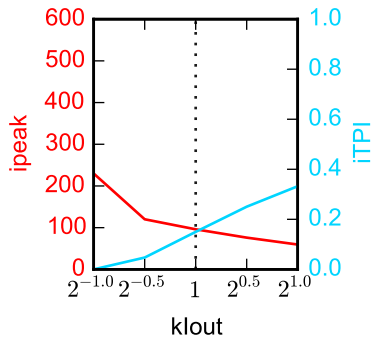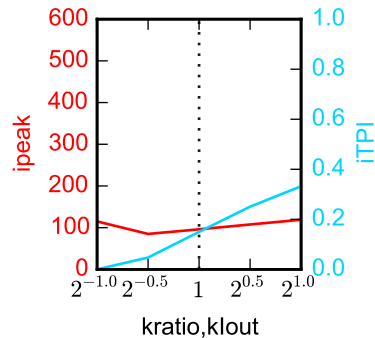

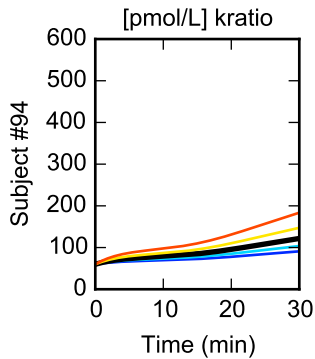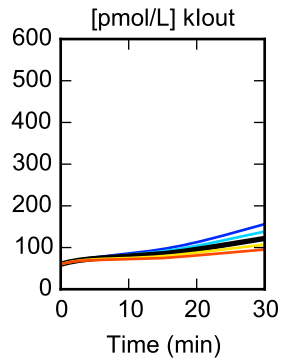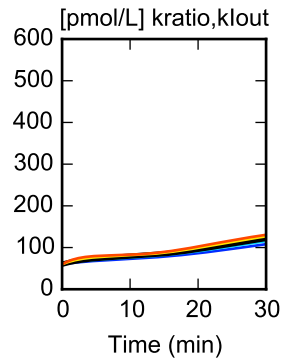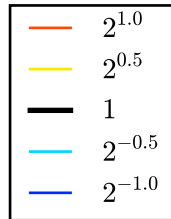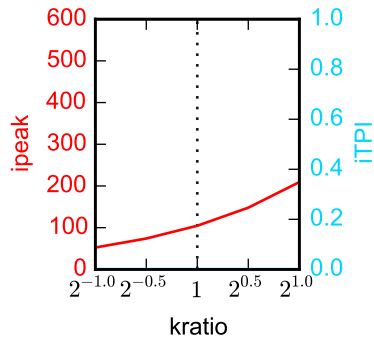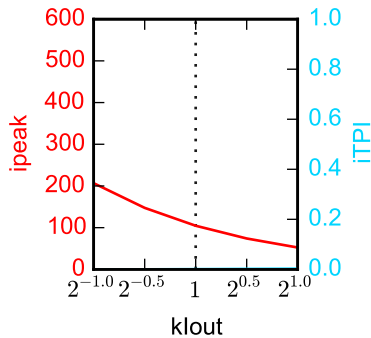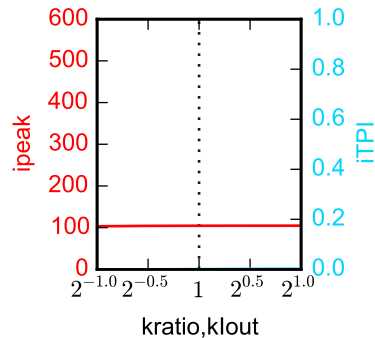

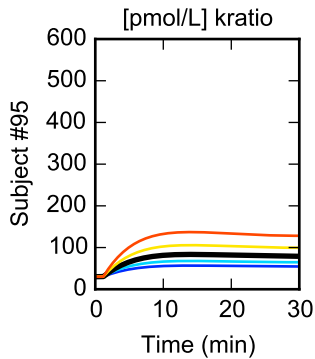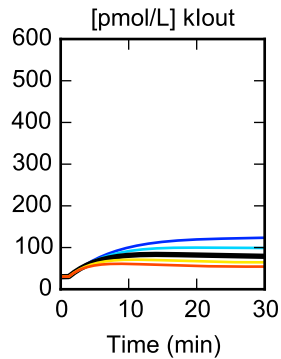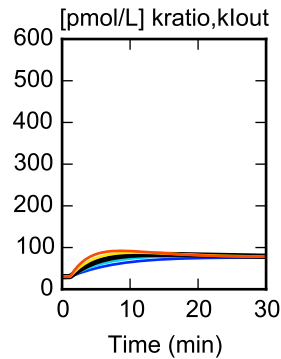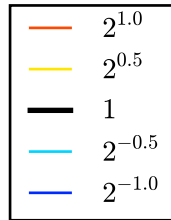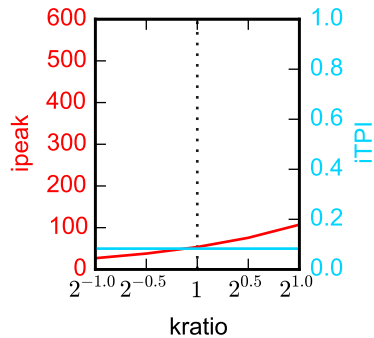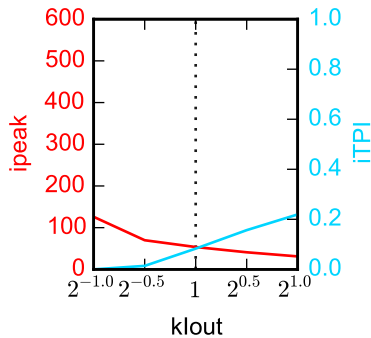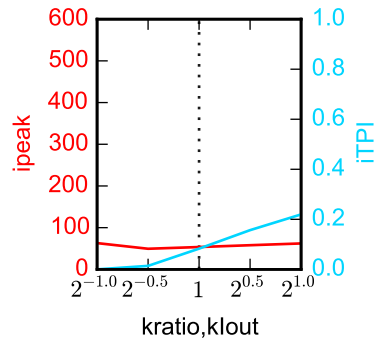

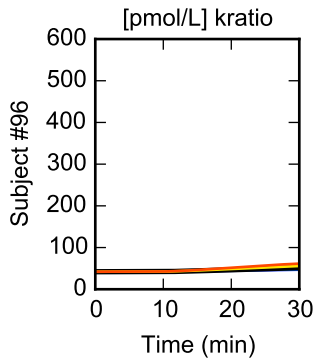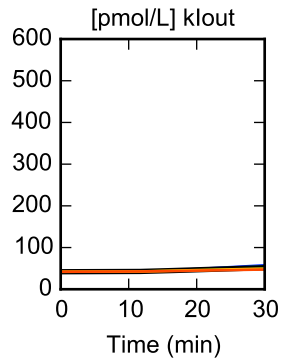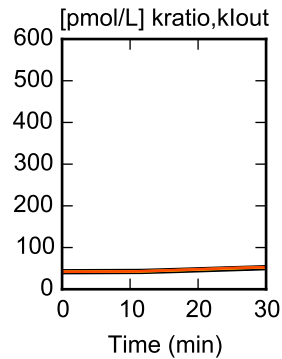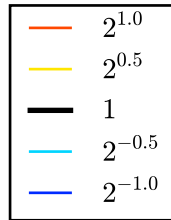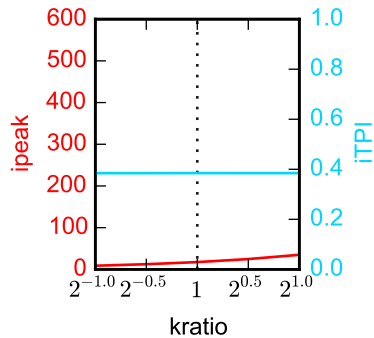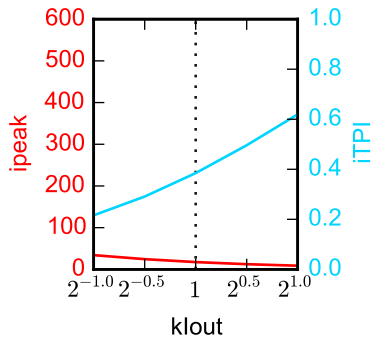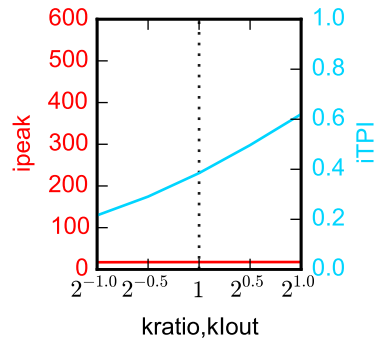

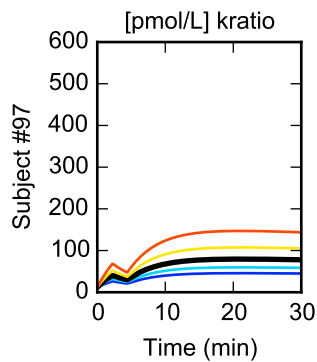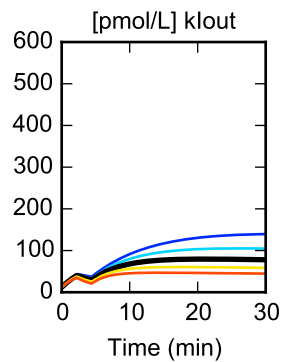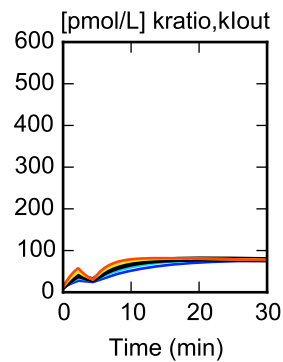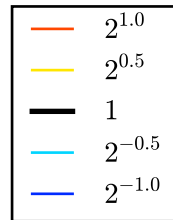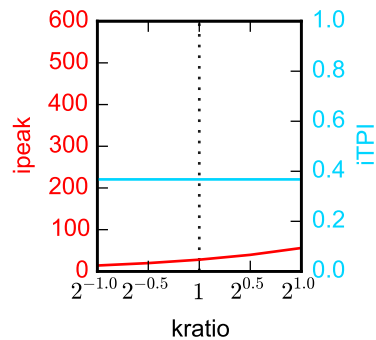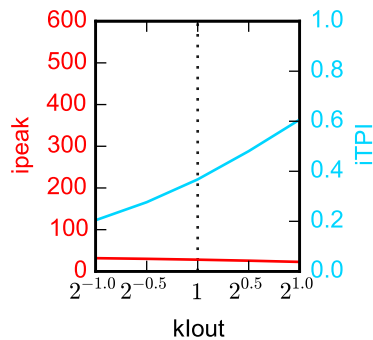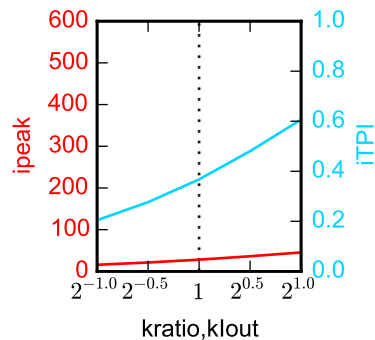

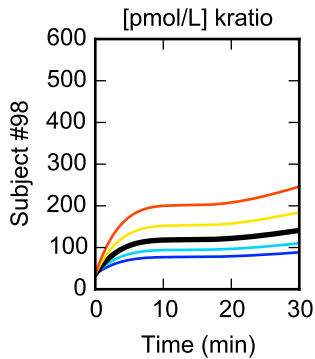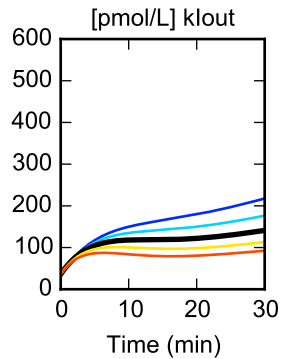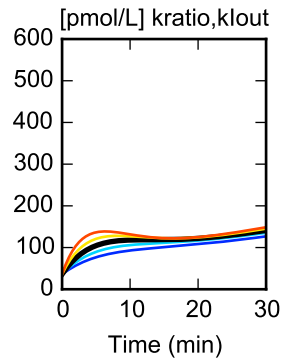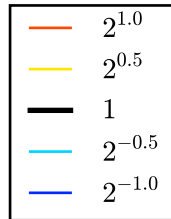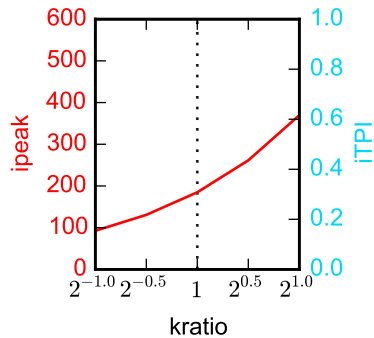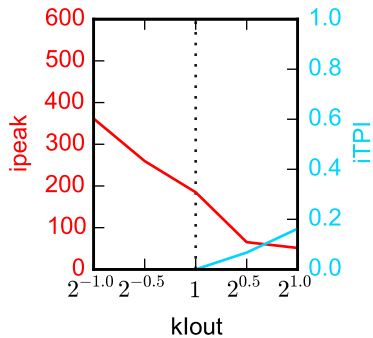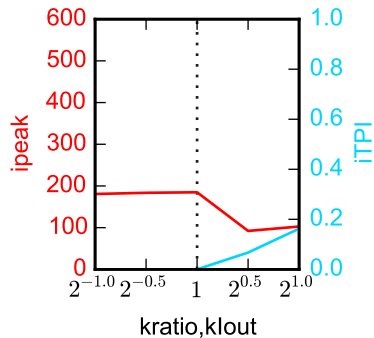

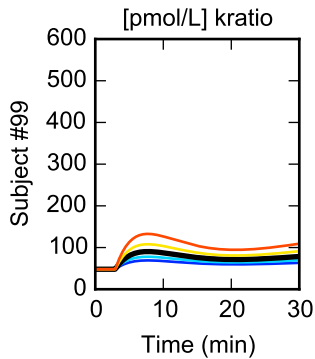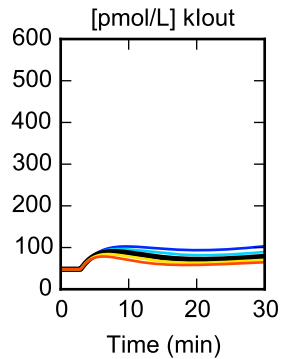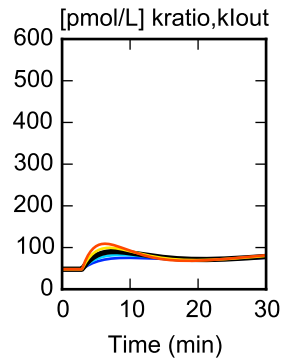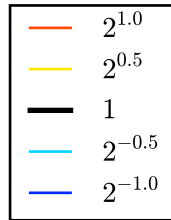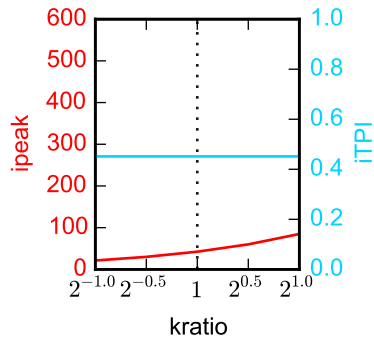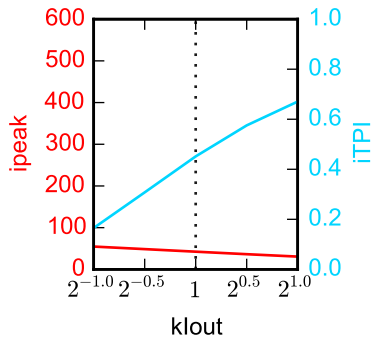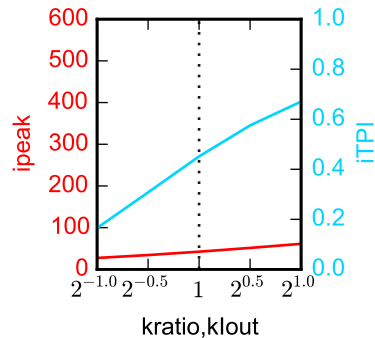

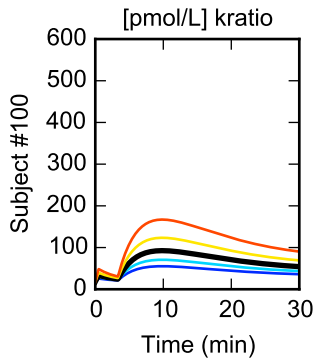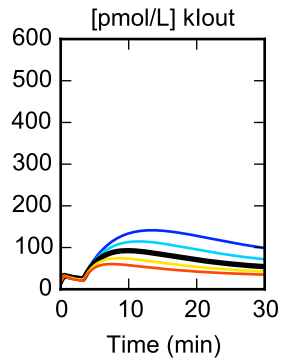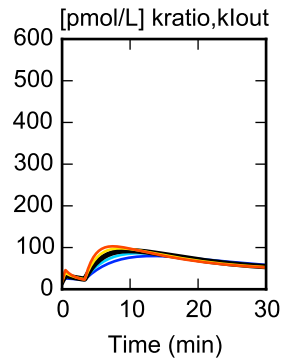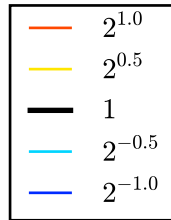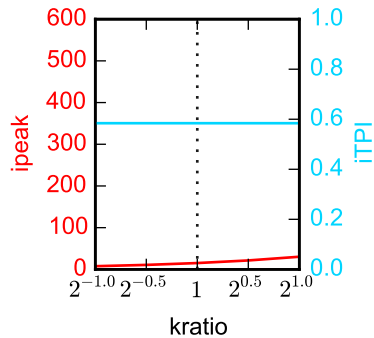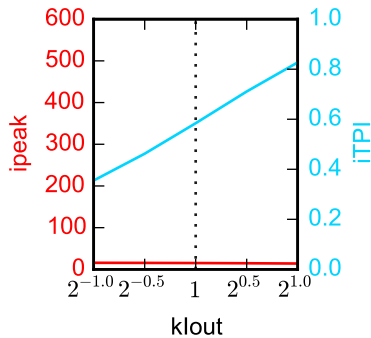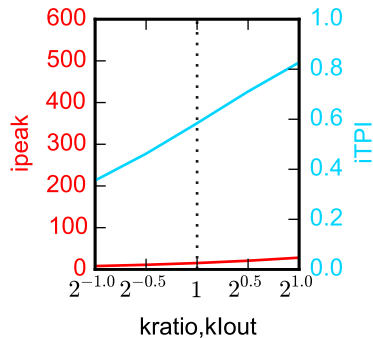

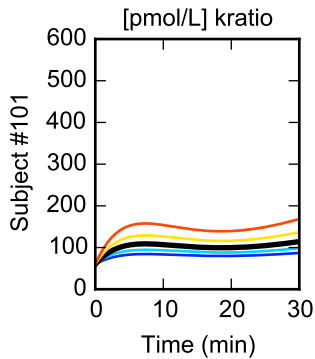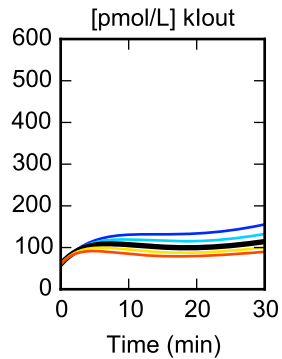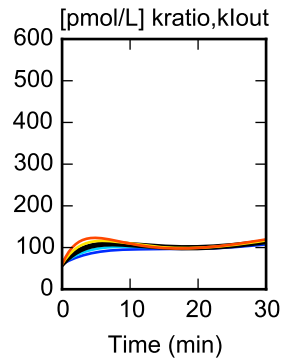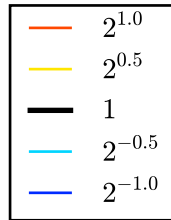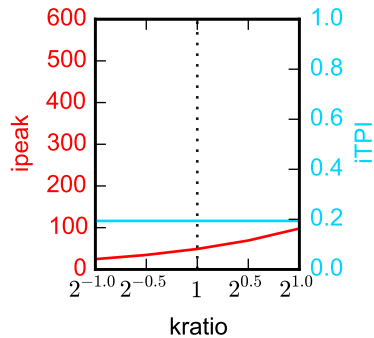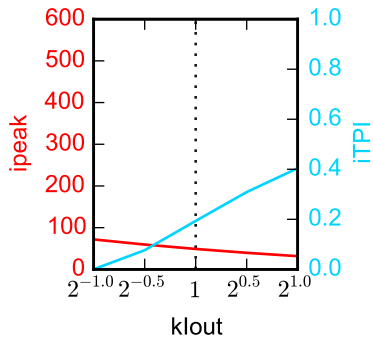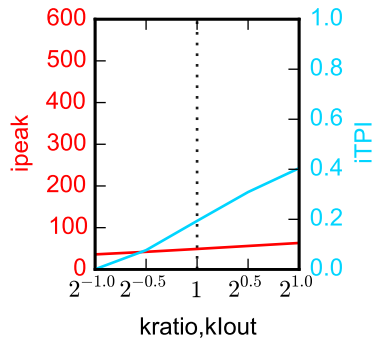

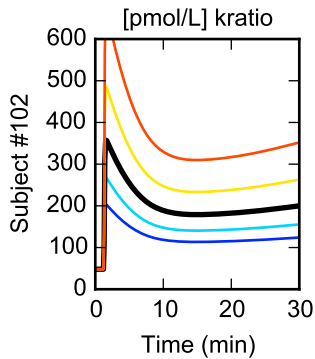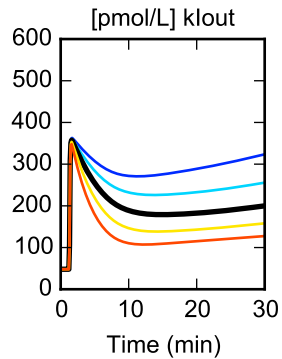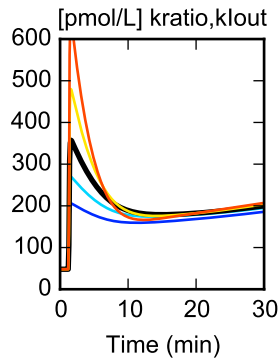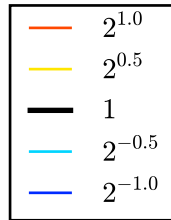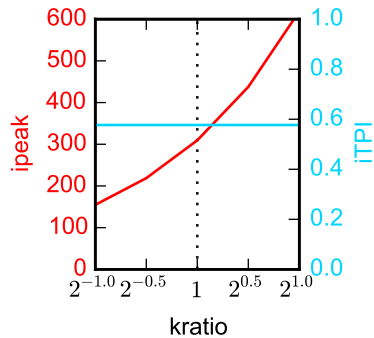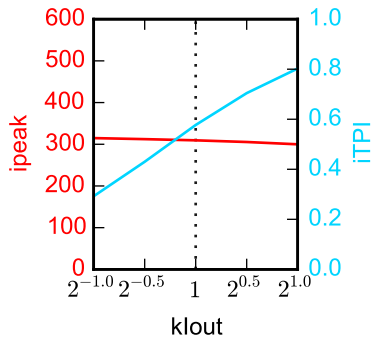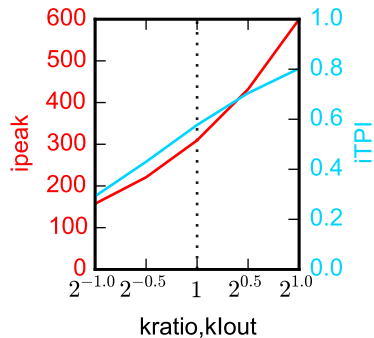

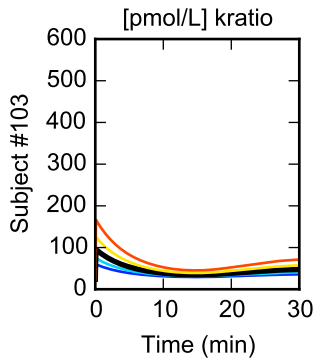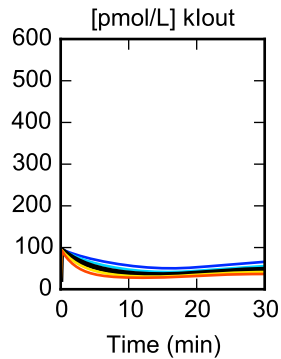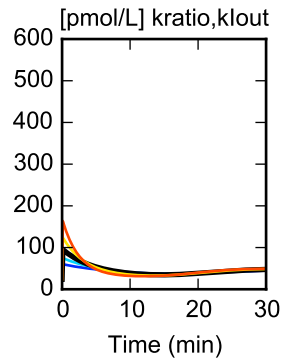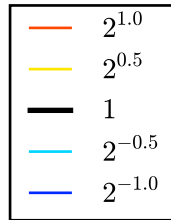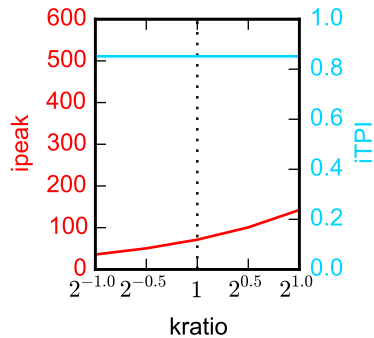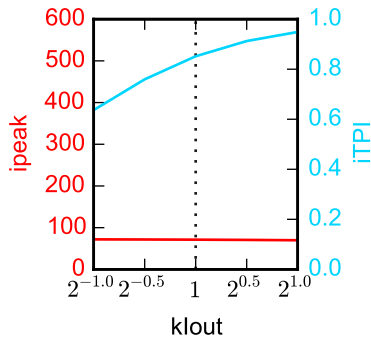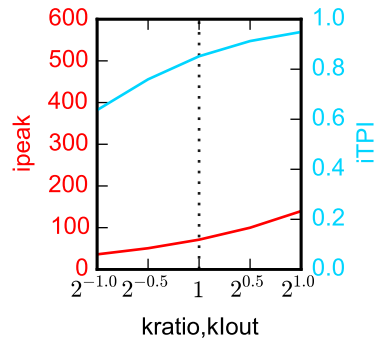

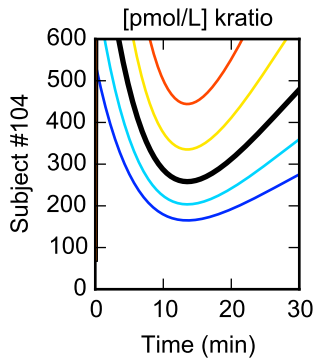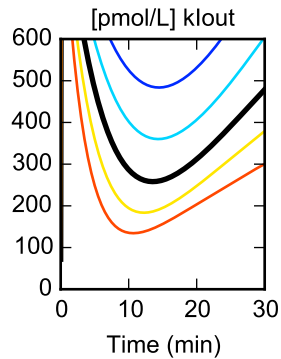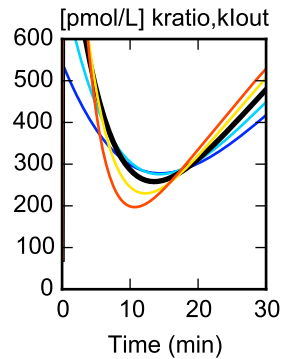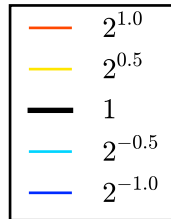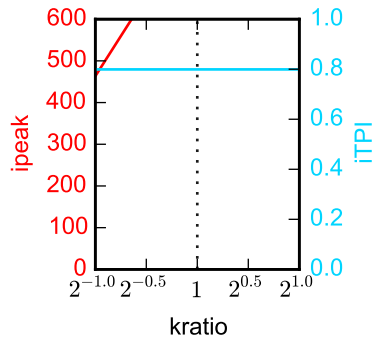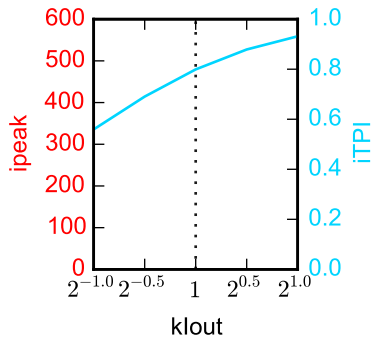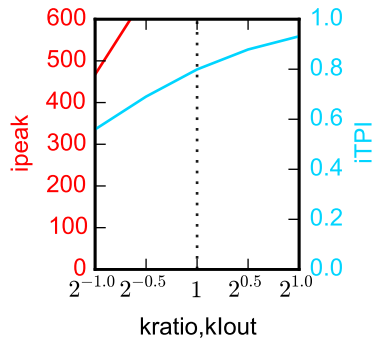

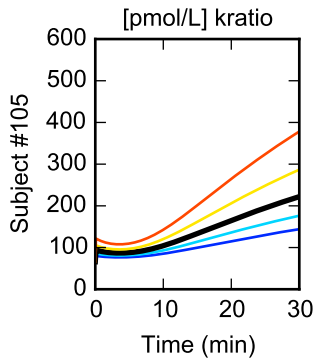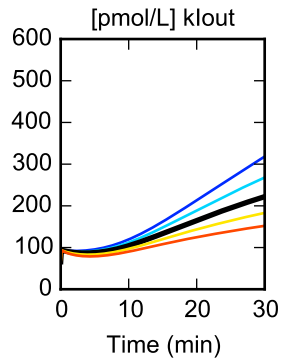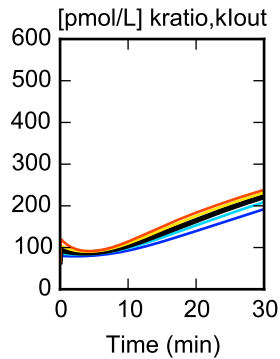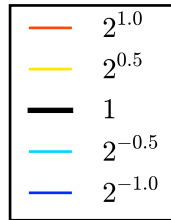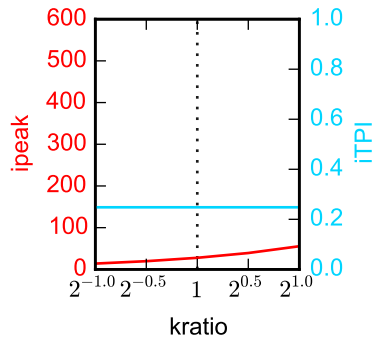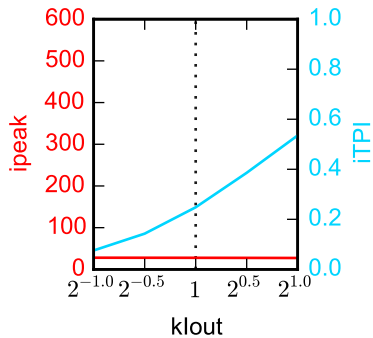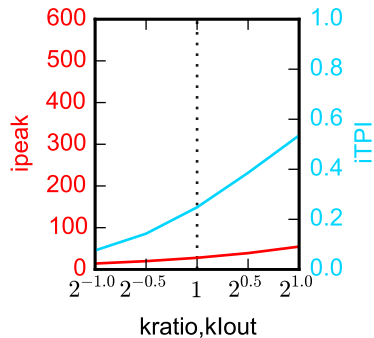

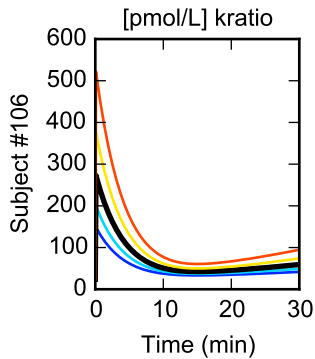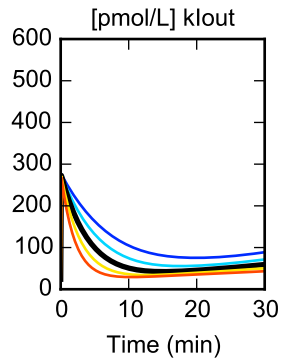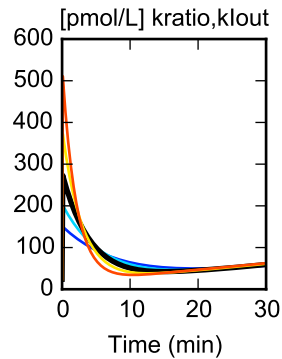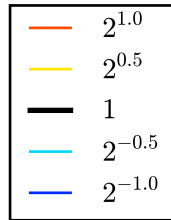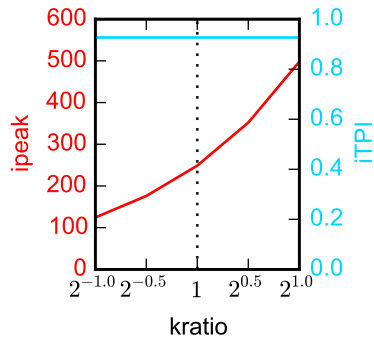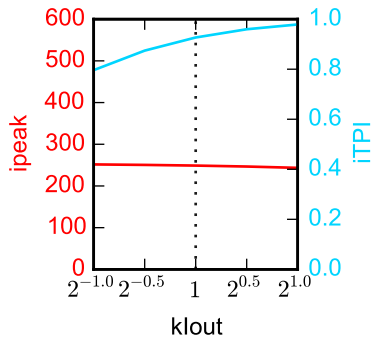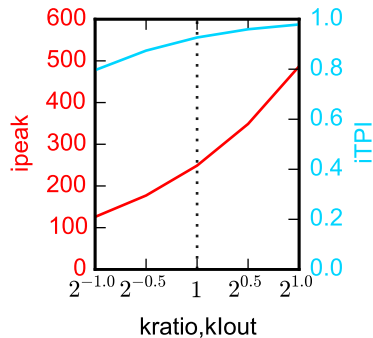

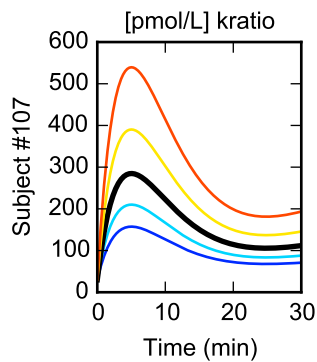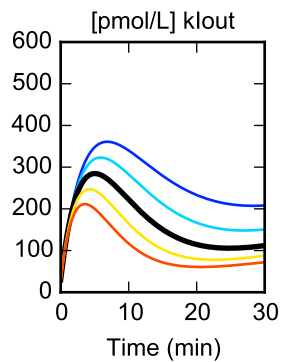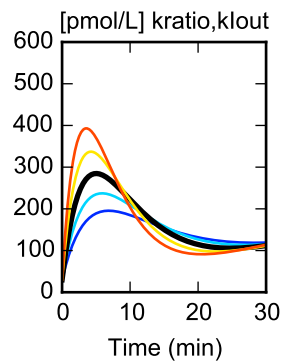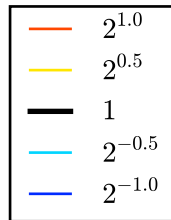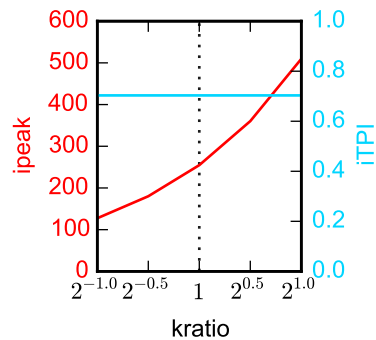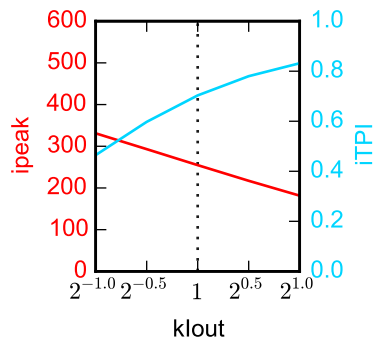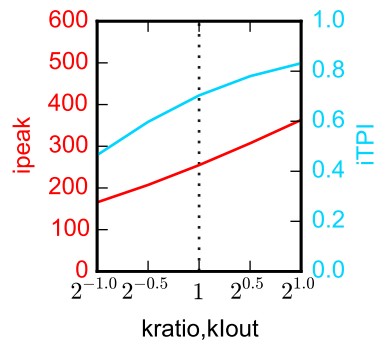

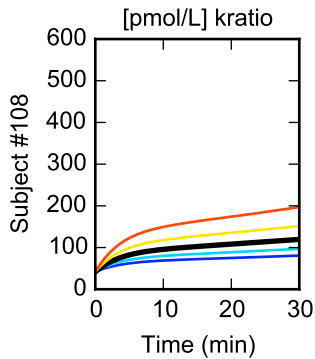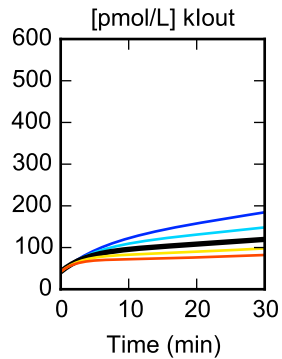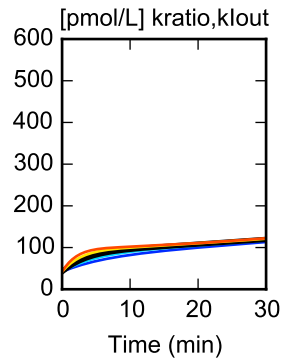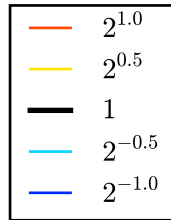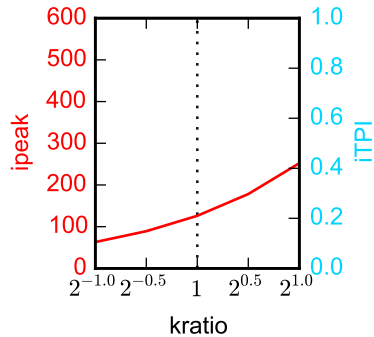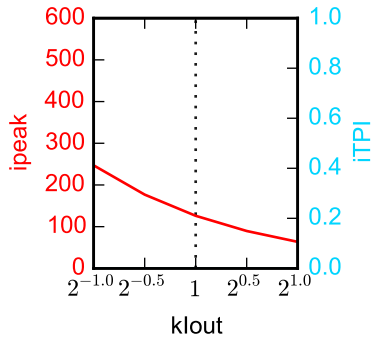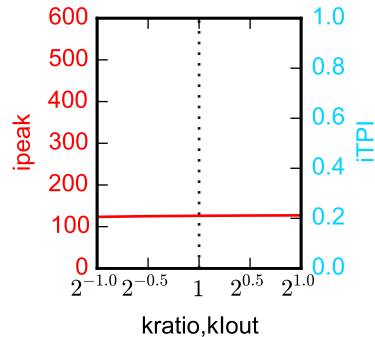

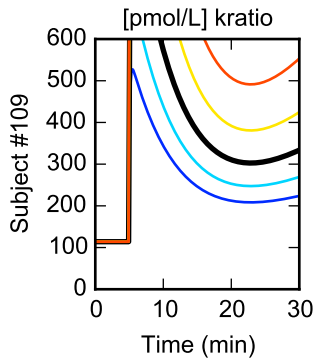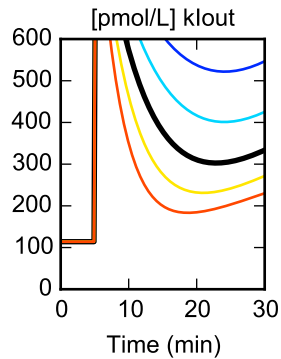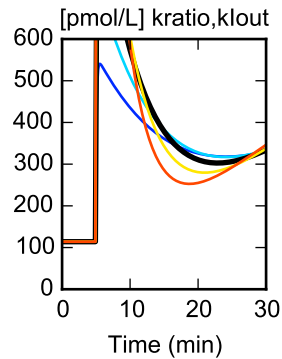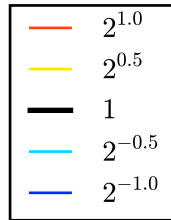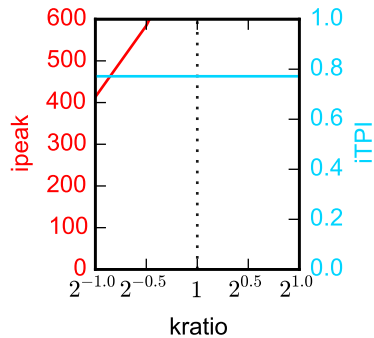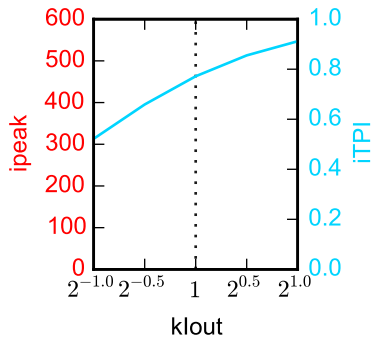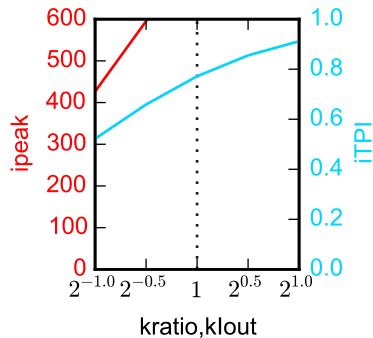

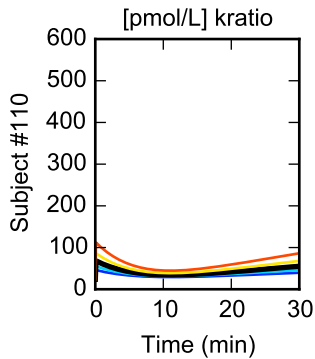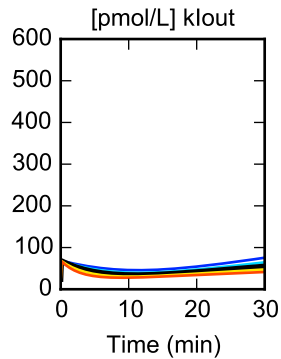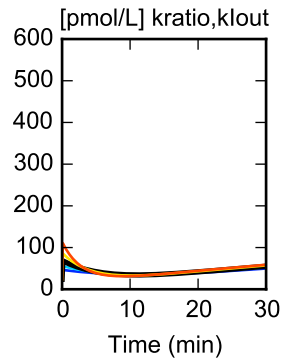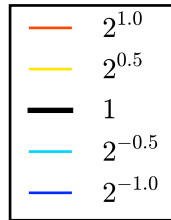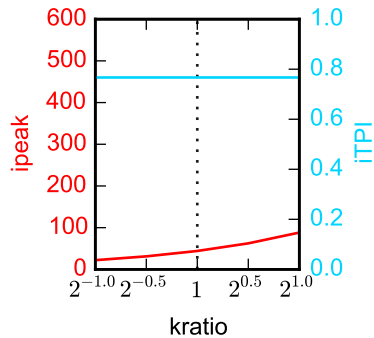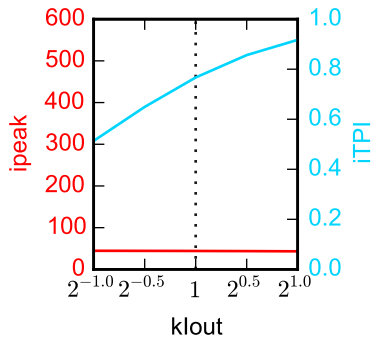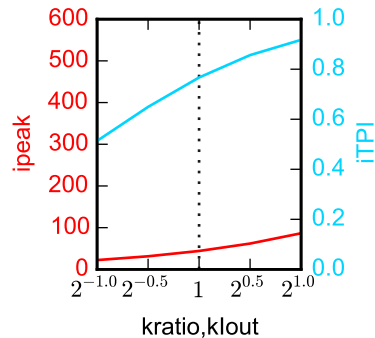

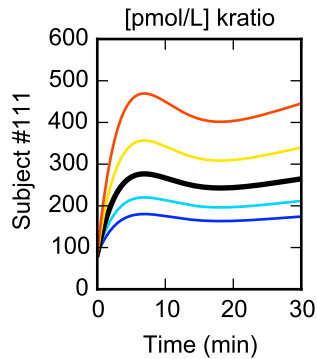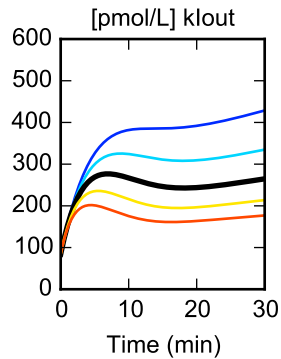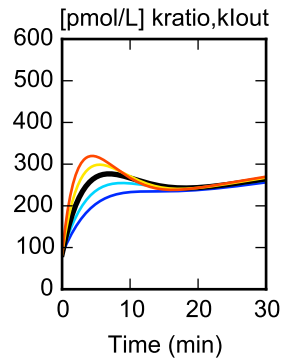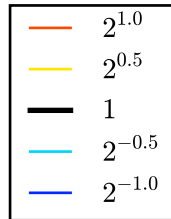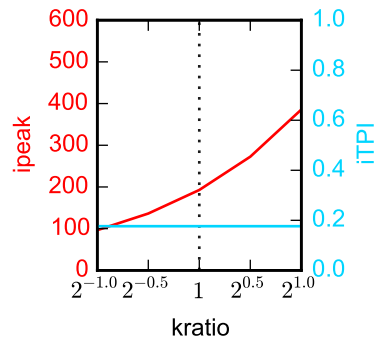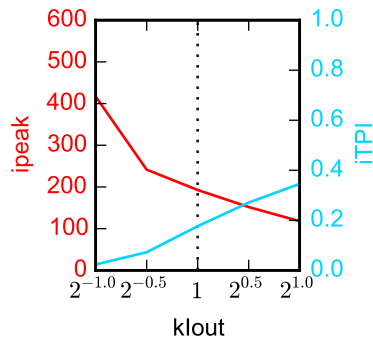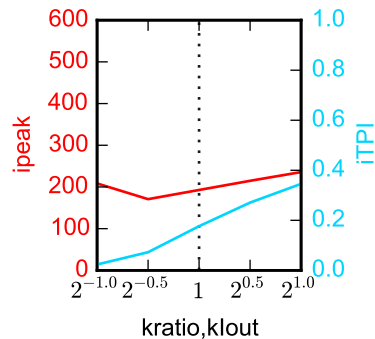

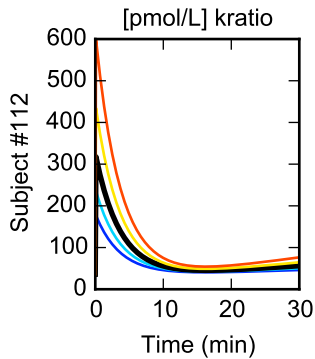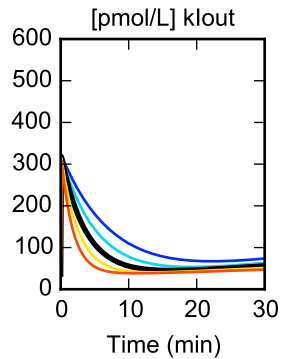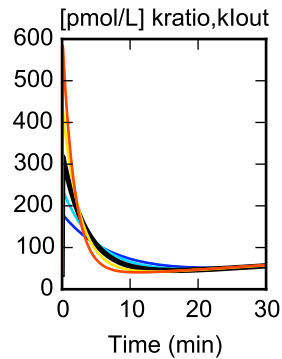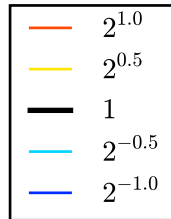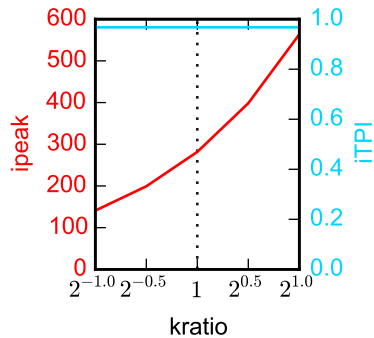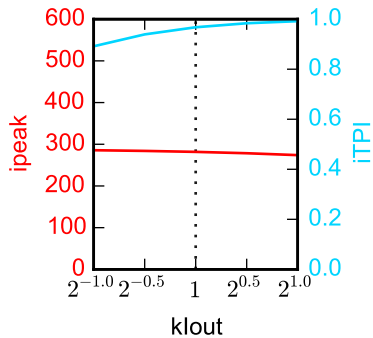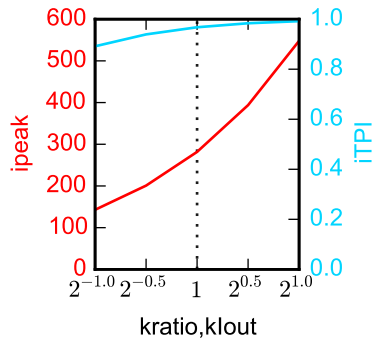

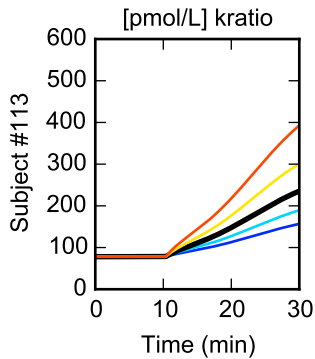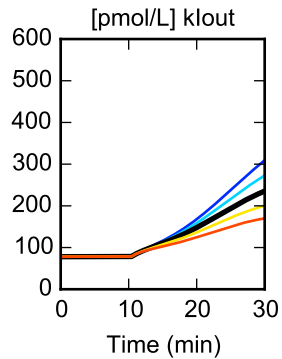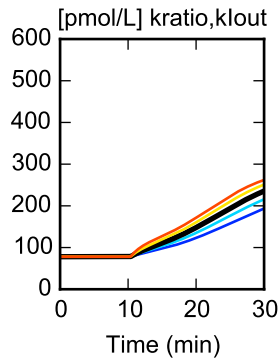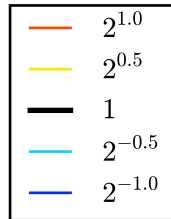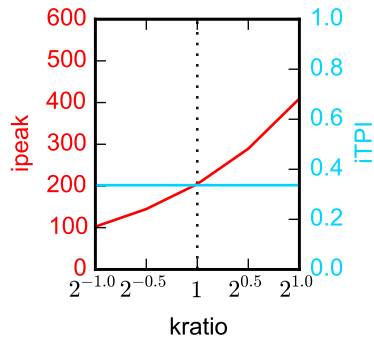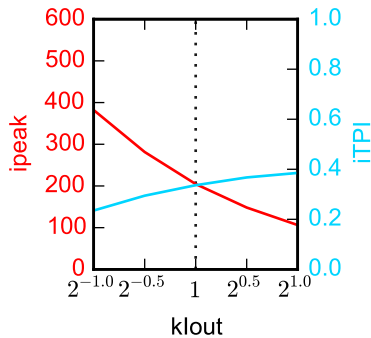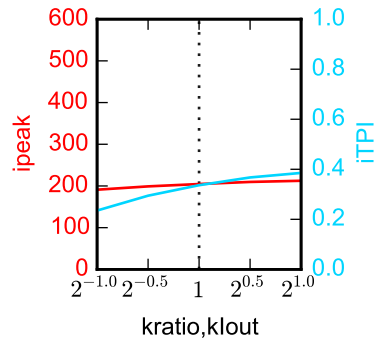

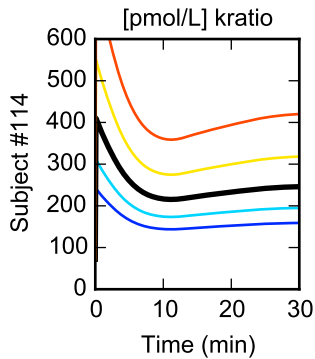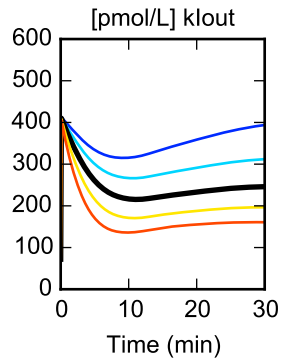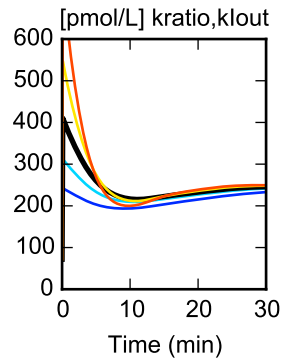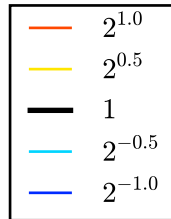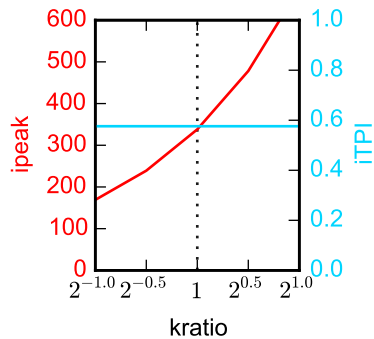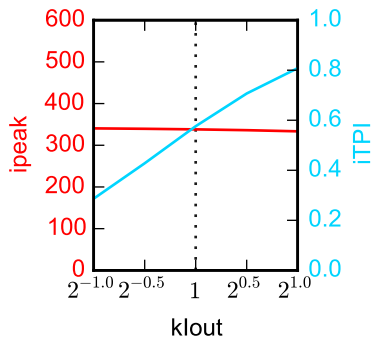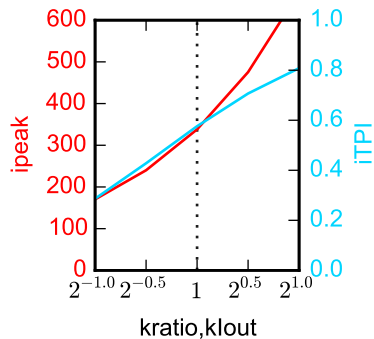

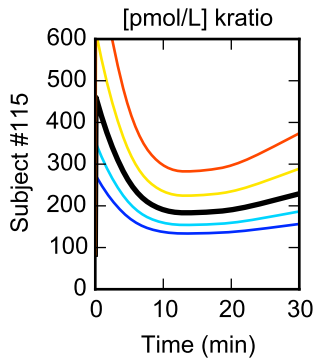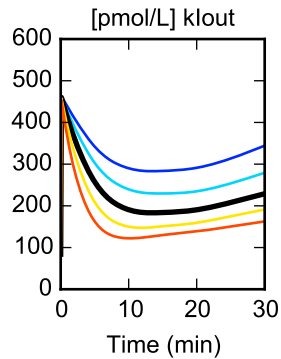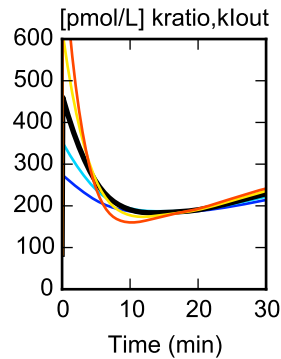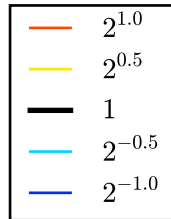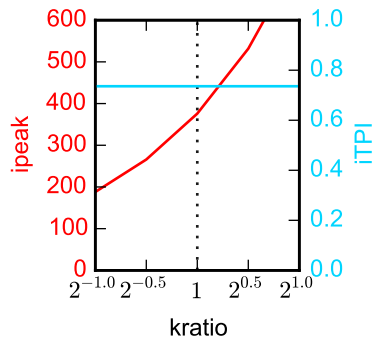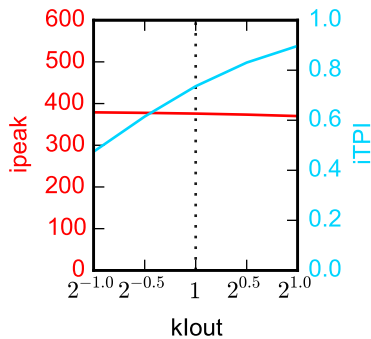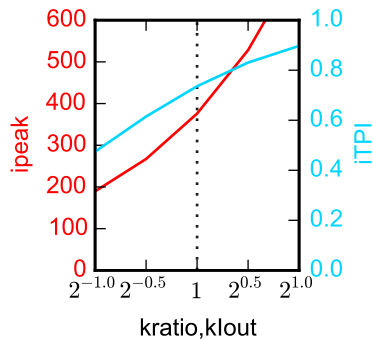

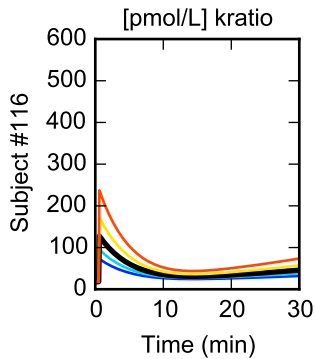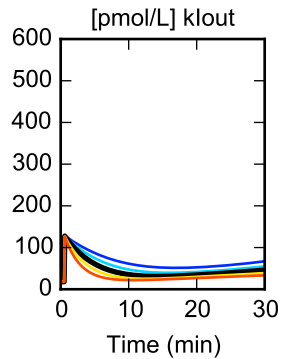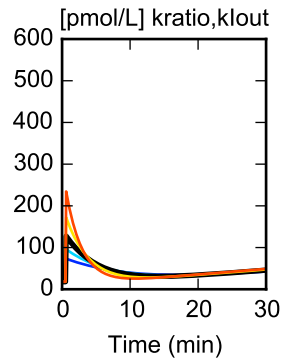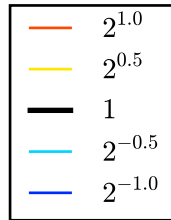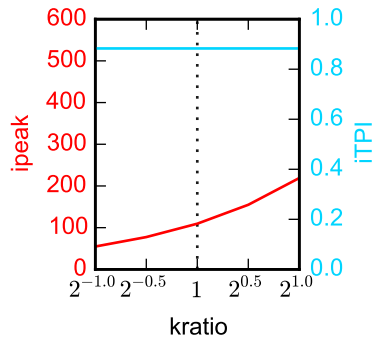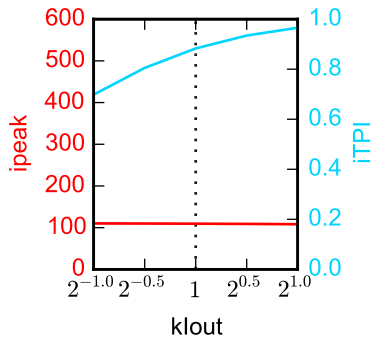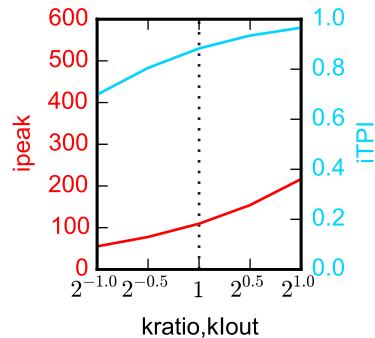

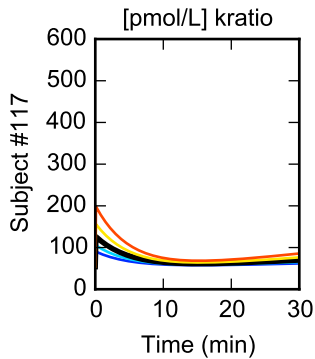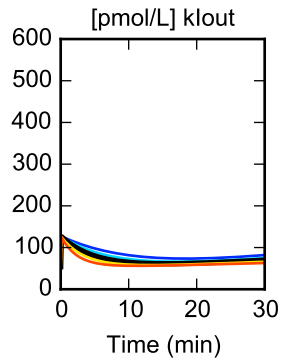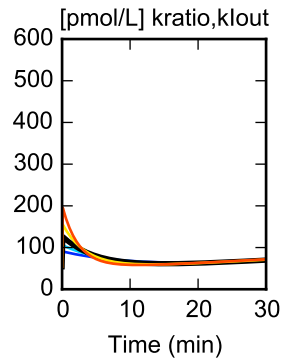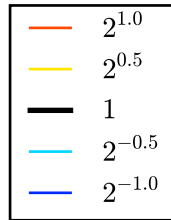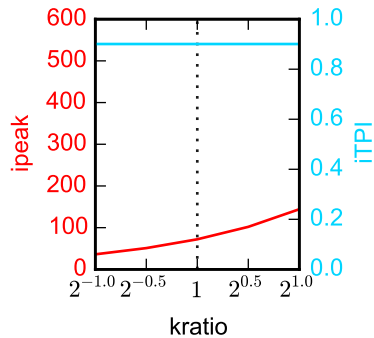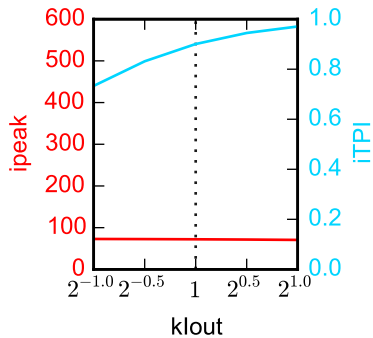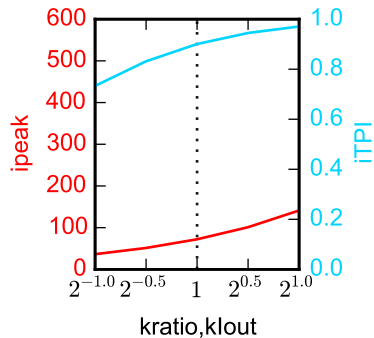

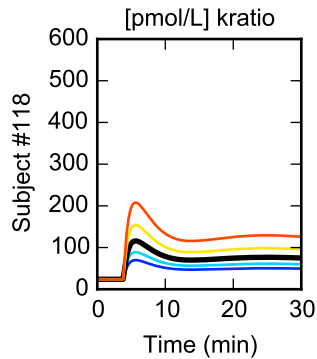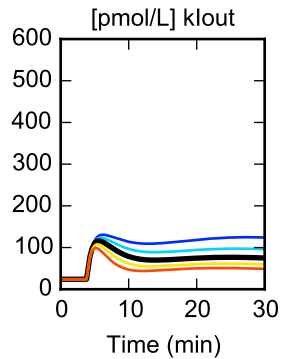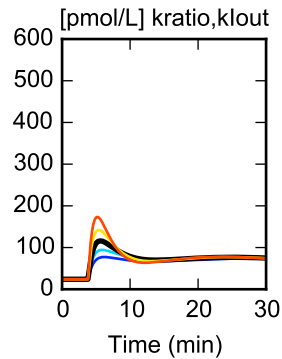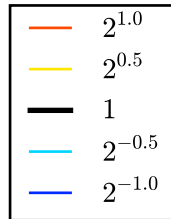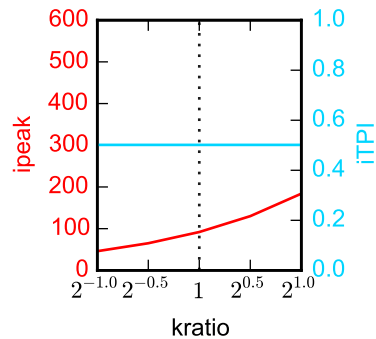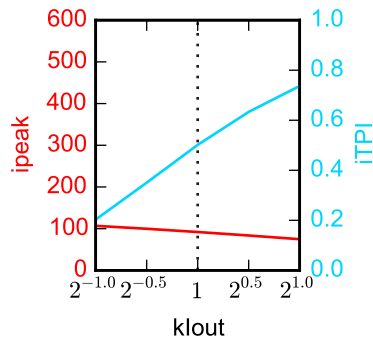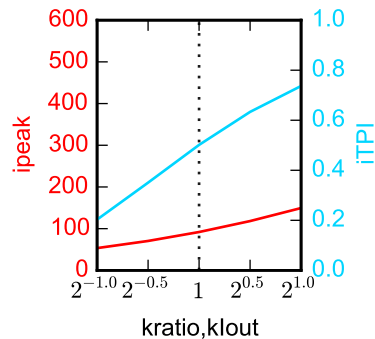

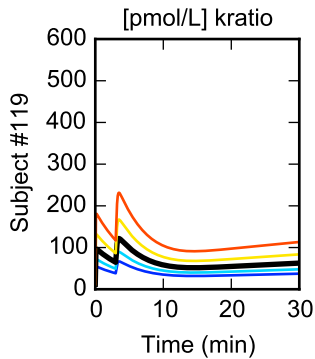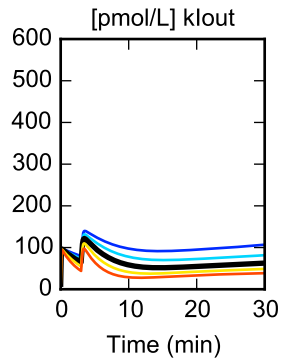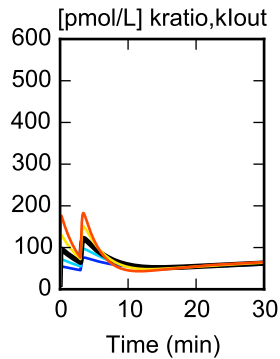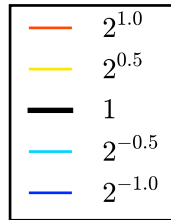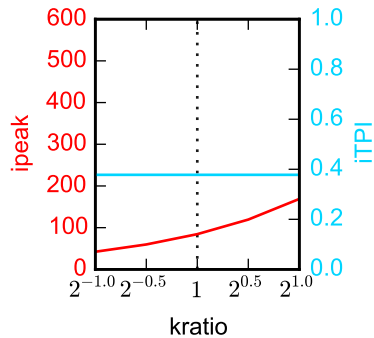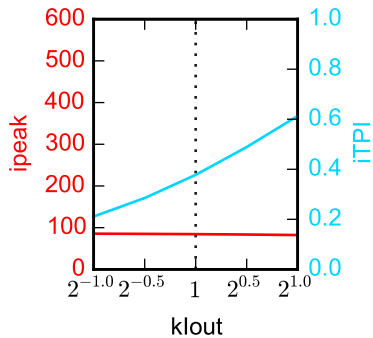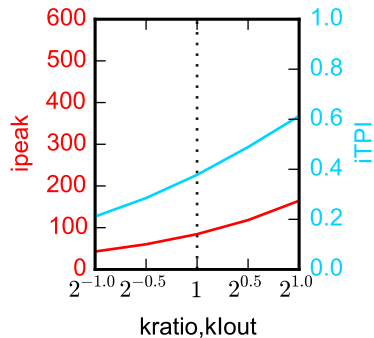

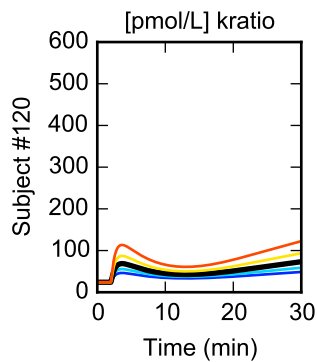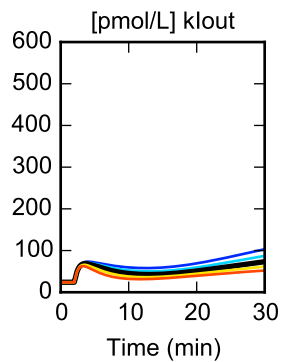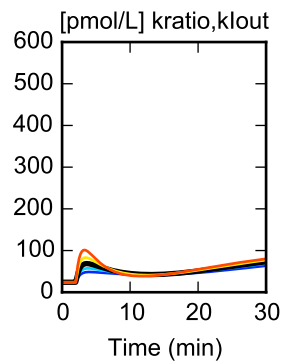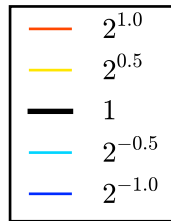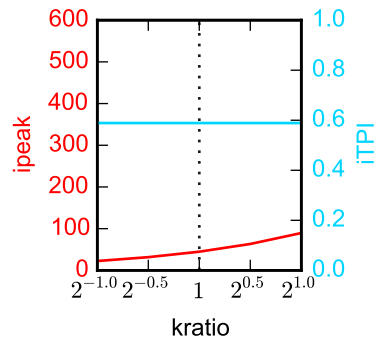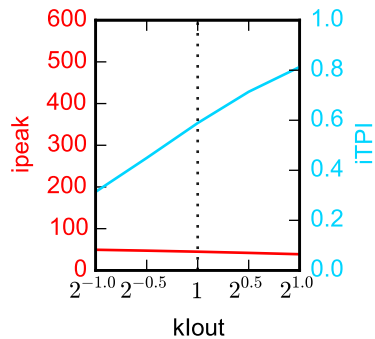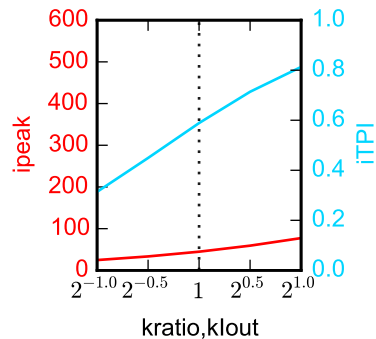

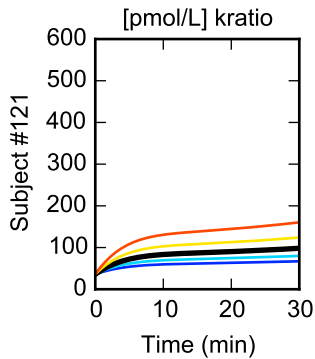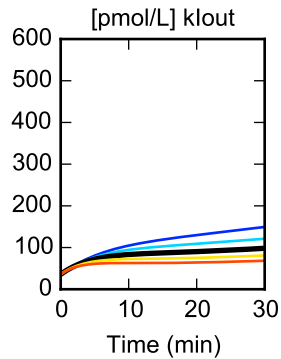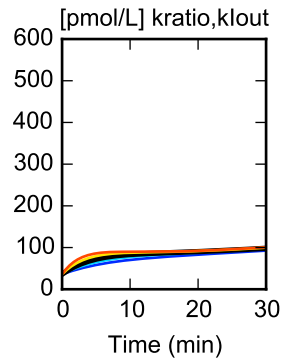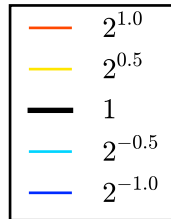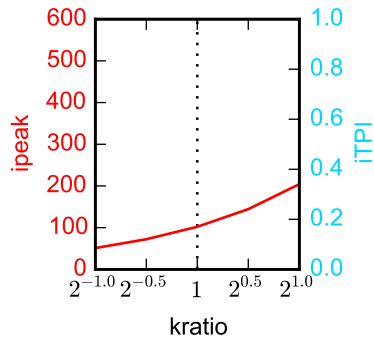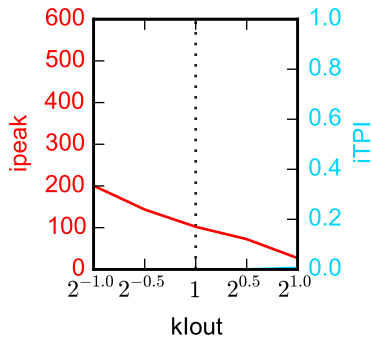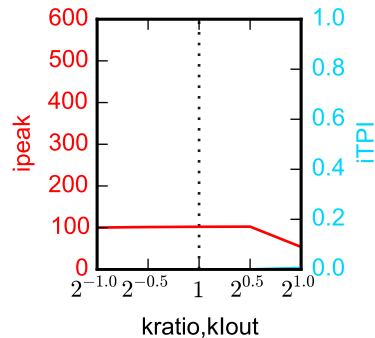

Supplement: Supplementary file 2 — Supplementary Figure S6 [file 41540_2018_51_MOESM2_ESM.pdf]
